# Identification and Biological Characterization of the Pyrazolo[3,4-*d*]pyrimidine Derivative SI388 Active as Src Inhibitor

**DOI:** 10.3390/ph16070958

**Published:** 2023-07-04

**Authors:** Claudia Contadini, Claudia Cirotti, Anna Carbone, Mehrdad Norouzi, Annarita Cianciusi, Emmanuele Crespan, Cecilia Perini, Giovanni Maga, Daniela Barilà, Francesca Musumeci, Silvia Schenone

**Affiliations:** 1 Laboratory of Cell Signaling, IRCCS-Fondazione Santa Lucia, 00179 Rome, Italy; claudiacontadini@gmail.com (C.C.); claudiacirotti89@gmail.com (C.C.);; 2 Department of Biology, University of Rome “Tor Vergata”, 00133 Rome, Italy; 3 Department of Pharmacy, University of Genoa, Viale Benedetto XV, 3, 16132 Genoa, Italyannar.cianciusi@gmail.com (A.C.); schenone@difar.unige.it (S.S.); 4 Institute of Molecular Genetics (IGM), IGM-CNR, Via Abbiategrasso 207, 27100 Pavia, Italygiovanni.maga@igm.cnr.it (G.M.)

**Keywords:** Src kinase, glioblastoma, radiotherapy resistance, pyrazolo[3,4-*d*]pyrimidines

## Abstract

Src is a non-receptor tyrosine kinase (TK) whose involvement in cancer, including glioblastoma (GBM), has been extensively demonstrated. In this context, we started from our *in-house* library of pyrazolo[3,4-*d*]pyrimidines that are active as Src and/or Bcr-Abl TK inhibitors and performed a lead optimization study to discover a new generation derivative that is suitable for Src kinase targeting. We synthesized a library of 19 compounds, **2a-s**. Among these, compound **2a** (SI388) was identified as the most potent Src inhibitor. Based on the cell-free results, we investigated the effect of SI388 in 2D and 3D GBM cellular models. Interestingly, SI388 significantly inhibits Src kinase, and therefore affects cell viability, tumorigenicity and enhances cancer cell sensitivity to ionizing radiation.

## 1. Introduction

Src is a non-receptor tyrosine kinase (TK) belonging to the Src family kinases (SFKs), which comprises different members, including Fyn. Src involvement in cancer has been extensively demonstrated; indeed, Src was the first TK and the first oncogene ever identified [1,2,3]. Several studies have shown Src kinase deregulation in different tumors; interestingly, Src hyperactivation is rarely due to mutations or gene duplication, while it is mainly linked to the aberrant activation of upstream receptor tyrosine kinases (RTKs) including EGFR, PDGFR, and MET. Src constitutive activation results in aberrant cell proliferation, survival, and migration and sustains angiogenesis, metastasis development, and resistance to therapy [4].

We previously developed a wide library of pyrazolo[3,4-*d*]pyrimidines active as Src, Fyn, and/or Bcr-Abl (another cytoplasmic TK) inhibitors. Many derivatives exhibited activity on different in vitro and in vivo tumor models characterized by a deregulation of the above mentioned kinases, such as osteosarcoma, chronic myelogenous leukemia (CML), neuroblastoma (NB), and glioblastoma (GBM) [5,6,7,8,9].

**Figure 1:**
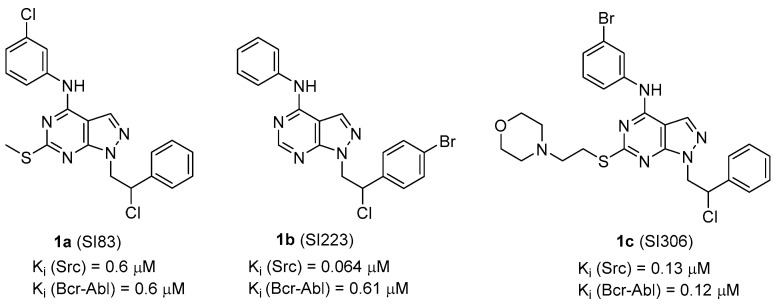
Structure and enzymatic activity of *in-house* 4-anilino-substituted pyrazolo[3,4-*d*]pyrimidines **1a–c**.

In several cases, the most active compounds were decorated with an anilino group at the C4 position. For instance, derivative **1a** (SI83) was active in a xenograft mouse model of osteosarcoma [9], compound **1b** (SI223) reduced more than 50% of the tumor volume in mice inoculated with 32D-T315I CML cells [5], and compound **1c** (SI306) showed activity in in vivo models of NB and GBM [6,7] (Figure 1).

Starting from these data, we decided to synthesize a library of 19 compounds, **2a-s,** to extend the structure–activity relationship (SAR) of this class of inhibitors. Compounds **2a-s** (Table 1) were designed by partially combining the structural features of the lead compounds **1a-c** and evaluated in enzymatic assays against Src, Abl and Fyn, based on the activity of *in-house* pyrazolo[3,4-*d*]pyrimidines that were previously synthesized. Interestingly, this study led to the identification of **2a** (SI388), a quite potent Src inhibitor endowed with increased activity compared the lead compound **1a**.

Based on these cell-free results, we decided to investigate the effect of SI388 using 2D and 3D GBM cellular models in which Src activity has been previously reported to be aberrantly activated [10,11].

We provide evidence of the ability of SI388 to affect cell viability and tumorigenicity via Src inhibition. Furthermore, we demonstrate that SI388 increases cell sensitivity to ionizing radiation.

## 2. Results and Discussion

### 2.1. Chemistry

We designed the new library of compounds **2a-s** (Table 1) to expand our SAR knowledge and identify new molecules endowed with improved activity profiles compared to the previously synthesized derivatives. With this aim, we decorated the pyrazolo[3,4-*d*]pyrimidine scaffold by introducing a few substituents present in our lead compounds **1a-c** and combined them with new chains, which had never introduced before. Herein, we report the C6 substituted compounds.

In detail, we planned to explore the chemical space around the C6 position by synthesizing some C6 thioalkyl derivatives (compounds **2a-g** and **2p**) as analog compounds of **1a** and **1b** and introducing different alkyl chains that had never been investigated before (compounds **2h-o** and **2q-s**). In particular, among the sulfur-containing compounds, we prepared the thiomethyl derivatives **2a** and **2b** to evaluate the effect of moving the chlorine atom from the meta (compound **1a**) to the ortho (**2a**) and para (**2b**) positions. Furthermore, we investigated the effect of the presence of an unsubstituted (**2c, h, j, k, n, p-s**) or a substituted (**2d-g, i, l, m, o**) anilino ring at the C4 position. In particular, we decorated the aromatic ring in C4 with different halogen atoms and, in a few cases, with a hydroxyl group since this polar group was recently shown to increase the enzymatic activity of our in-house 4-amino pyrazolo[3,4-d]pyrimidines [12,13]. Finally, we functionalized unsubstituted anilino derivatives **2p-s** with a halogen atom on the para position of the N1 2-chloro-2-phenylethyl side chain, affording structural analogs of compound **1c**.

**Scheme 1:**
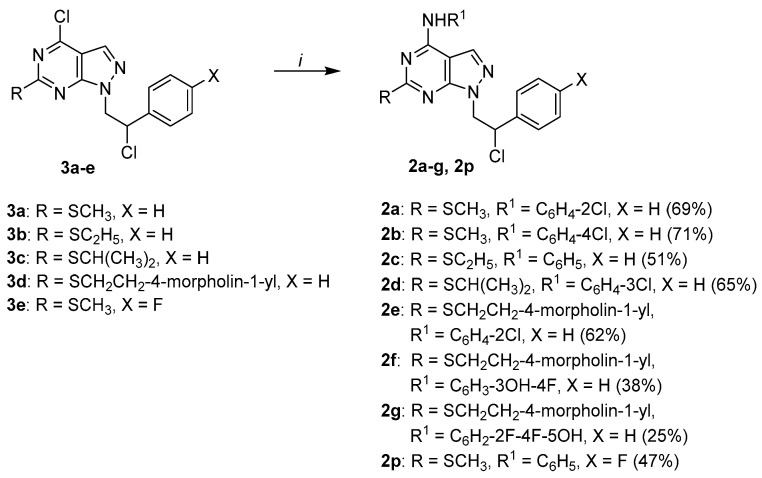
Synthesis of compounds **2a-g** and **2p**. ^a^ **Reagents and conditions:** (i) appropriate aniline, abs EtOH, reflux, 3–5 h, 25–71%.

**Scheme 2:**
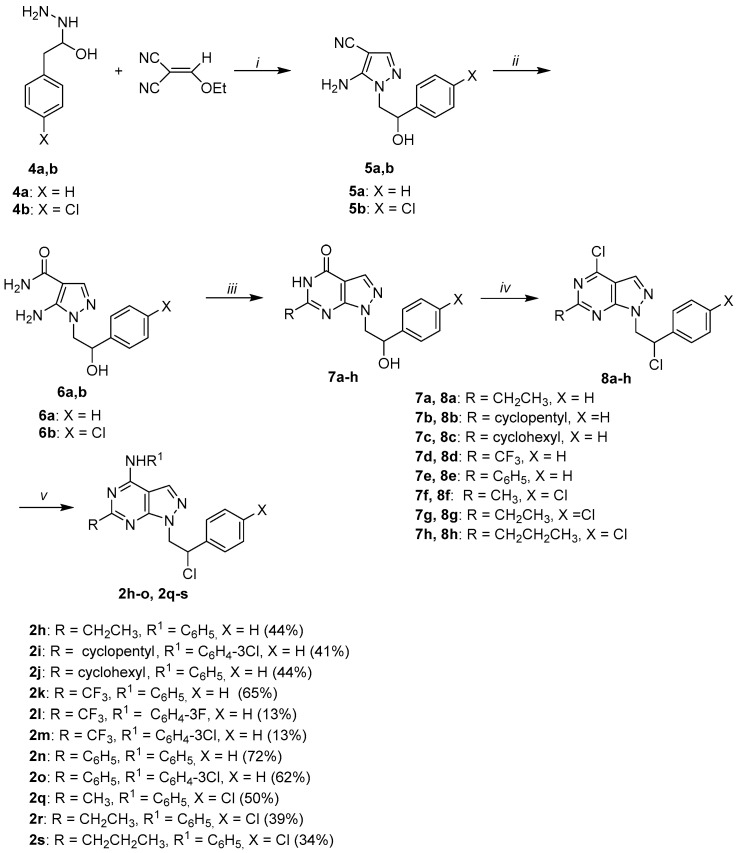
Synthesis of compounds **2h-o** and **2q-s**. ^b^ **Reagents and conditions:** (i) abs EtOH, reflux, 6 h, 51.71%; (ii) 2M NaOH, abs EtOH, reflux, 2 h, 58,66%; (iii) appropriate alkyl ester, EtONa, abs EtOH, reflux, 6–12 h, 44–80%; (iv) POCl_3_/DMF, CHCl_3_, reflux, 6–12 h, 81–98%; (v) appropriate aniline, abs EtOH, reflux, 5 h (to obtain compounds **2h-j, 2n,o,** and **2q-s**, 34–72%), or appropriate aniline, abs EtOH, MW, 80 °C, P_atm_, 2–10 min (to obtain compounds **2k-m**, 13–65%).

The synthesis of compounds **2a-s** was performed following a multistep route reported in Scheme 1 and Scheme 2.

For the preparation of C6-thioalkyl derivatives **2a-g** and **2p**, we exploited a general chemical route, already reported by us for the synthesis of analog compounds [8,9]. In the last step, we reacted the 4-chloro pyrazolo[3,4-*d*]pyrimidines **3a-e** [8,9,14,15] with the suitable aniline in the presence of absolute ethanol at reflux to afford the corresponding amino derivatives via a nucleophilic substitution (Scheme 1).

We followed a different route for synthesizing C6-alkyl derivatives (Scheme 2). The opportune 2-hydrazino-1-phenylethanol **4a,b** [9] was reacted with ethoxymethylene-malononitrile at reflux for 6 h affording the 5-amino-1-(2-hydroxy-2-phenylethyl)-1*H*-pyrazole-4-carbonitrile intermediates **5a,b**. Hydrolysis of the cyano groups of **5a,b** to the corresponding carboxamides afforded compounds **6a,b**. Cyclization of **6a,b** by treatment with the appropriated alkyl esters and sodium ethylate in ethanol at reflux gave the 1-(2-hydroxy-2-phenylethyl)-6-alkyl-1,5-dihydro-4*H*-pyrazolo[3,4-*d*]pyrimidin-4-ones **7a-h**. Treatment of **7a-h** with the Vilsmeier complex (POCl_3_/DMF) in CHCl_3_ at reflux led to the dichloro derivatives **8a-h**. Finally, these intermediates were reacted with the appropriate aniline, in accordance with the conditions described in Scheme 1, to obtain the desired compounds **2h-j, 2n,o,** and **2q-s**. Otherwise, compounds **2k-m**, bearing a trifluoromethyl group at the C6 position, were obtained by MW-assisted synthesis at 80 °C for 2–10 min in an open vessel.

### 2.2. Enzymatic Activity

Pyrazolo[3,4-*d*]pyrimidines **2a-s** were evaluated in enzymatic assays against Src, Bcr-Abl, and Fyn, using compound **1a** as the reference. The percentage of inhibition was calculated at different concentrations (100, 10, and 1 μM) (Table S1, in the Supporting Information), and the K_i_ was determined for the most promising derivatives (Table 2).

Interestingly, compound **2a**, possessing K_i_ values of 0.423 and 0.419 µM for Src and Abl, respectively, emerged as a valuable dual Src/Bcr-Abl inhibitor [9,16]. On the other hand, the corresponding para substituted derivative **2b** was inactive.

Unfortunately, other derivatives also generally showed low activity in cell-free assays. The introduction of an alkyl chain at C6 (compounds **2h-o** and **2q-s**) did not afford potent SFK/Bcr-Abl inhibitors. This SAR could be explained by the fact that alkyl groups cannot form polar interactions with the target and stabilize the complex. Furthermore, it is worth pointing out that the polar thioethylmorpholinyl chain (compounds **2e-g**) also did not lead to effective Src/Fyn/Bcr-Abl inhibitors. This result is quite unexpected since other sets of 6-thioethylmorpholinyl pyrazolo[3,4-*d*]pyrimidines recently published by us [12,13] showed an activity profile towards Src and/or Bcr-Abl in the nanomolar range. In this case, we can hypothesize that the activity loss is due to the different substituents on the anilino ring. A careful comparison of previous and current data allowed us to shed light on this behavior and expand our SAR knowledge. Many of the best in-house thioethylmorpholinyl derivatives that are active as Src inhibitors bear a 2-chloro-5-hydroxy anilino group at C4 [12]. Modeling studies highlighted that the OH was able to form a hydrogen bond with a residue of glutamate of the catalytic pocket of Src, enhancing the affinity between the kinase and the inhibitor [7]. The current study further confirmed these in silico results: compound **2e** was inactive towards all of the tested kinases, while its hydroxylated analog compound possessed a K_i_ value of 190 nM towards Src [12]. Furthermore, data reported in Table 1 show that introducing a fluorine atom in the ortho or ortho/para positions of the anilino ring (compounds **2f** and **2g**) led to inactive compounds. We already reported that a 2-fluoro-3-hydroxy anilino derivative was less active than the corresponding chloro analog compound [12]; in the study, we also demonstrated that moving the fluorine to the para position or the presence of two fluorine atoms did not lead to positive effects on the activity.

Overall, this study allowed us to identify a low micromolar Src/Bcr-Abl inhibitor (**2a**). Because in-house C6 substituted pyrazolo-pyrimidines generally showed activity in tumors characterized by Src overexpression, we decided to investigate the activity of **2a** in GBM cellular models.

### 2.3. Cell Biology

#### 2.3.1. SI388 Inhibits Src Kinase Activity in GBM Cellular Models

To test the ability of compound **2a** (SI388) to inhibit Src activity in vitro, we took advantage of glioblastoma cellular models. Glioblastoma, indeed, was the first cancer type to be systematically studied by The Cancer Genome Atlas Research Network (TCGA)[17], who identified the RTK/Ras/PI3K pathway as having the most frequently deregulated signaling [4,18], with Src being a common node downstream of different RTKs [4,10].

**Figure 2:**
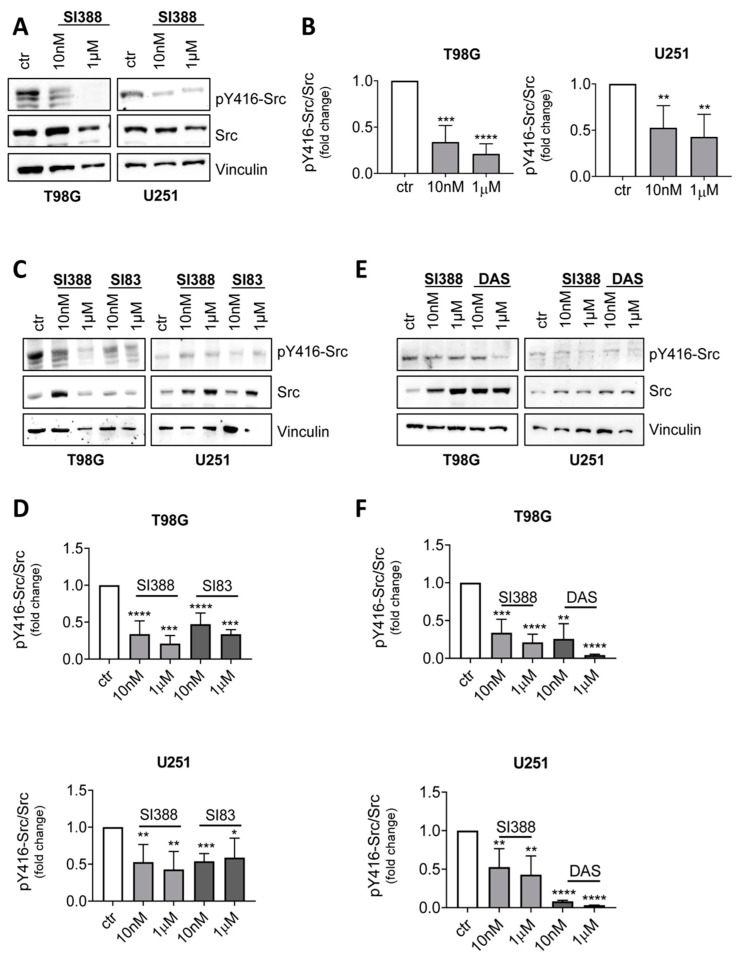
**SI388 inhibits Src activity in T98G and U251 cell lines.** (**A**,**B**) Western blot analysis (**A**) and relative quantification (**B**) of pY416-Src in T98G and U251 cell lines treated with SI388 10 nM and 1 µM for 24 h. Vinculin was used as loading control. (**C**,**D**) Western blot analysis (**C**) and relative quantification (**D**) of pY416-Src in T98G and U251 cell lines treated with SI388 or SI83 at two different concentrations for 24 h. Vinculin was used as loading control. (**E**,**F**) Western blot analysis (**E**) and relative quantification (**F**) of pY416-Src in T98G and U251 cell lines treated with SI388 or dasatinib (DAS) at two different concentrations for 24 h. Vinculin was used as loading control. Histograms summarize quantitative data of the mean ± S.E.M. of four independent experiments. Statistical analyses: unpaired Student’s *t*-test: * *p* < 0.05; ** *p* < 0.01; *** *p* < 0.001; **** *p* < 0.0001.

U251 and T98G glioblastoma cell lines were treated with two ascending drug concentrations (10 nM and 1 µM) or DMSO for 24 h and Src activity was revealed by immunoblotting with a specific anti-phosho Tyr416-Src (anti-pY416Src) antibody. As shown in Figure 2 (panels A and B), we observed a reduction in Src phosphorylation on Tyr416 (Y416) after treatment with **2a** in a dose-dependent manner. Similar results were also obtained upon treatment with **1a** (SI83) (Figure 2, panels C and D), its analogue compound, and dasatinib (DAS) (Figure 2, panels E and F), a well-known commercially available Src inhibitor.

#### 2.3.2. SI388 Reduces Cell Growth and Viability in GBM Cell Lines

**Figure 3:**
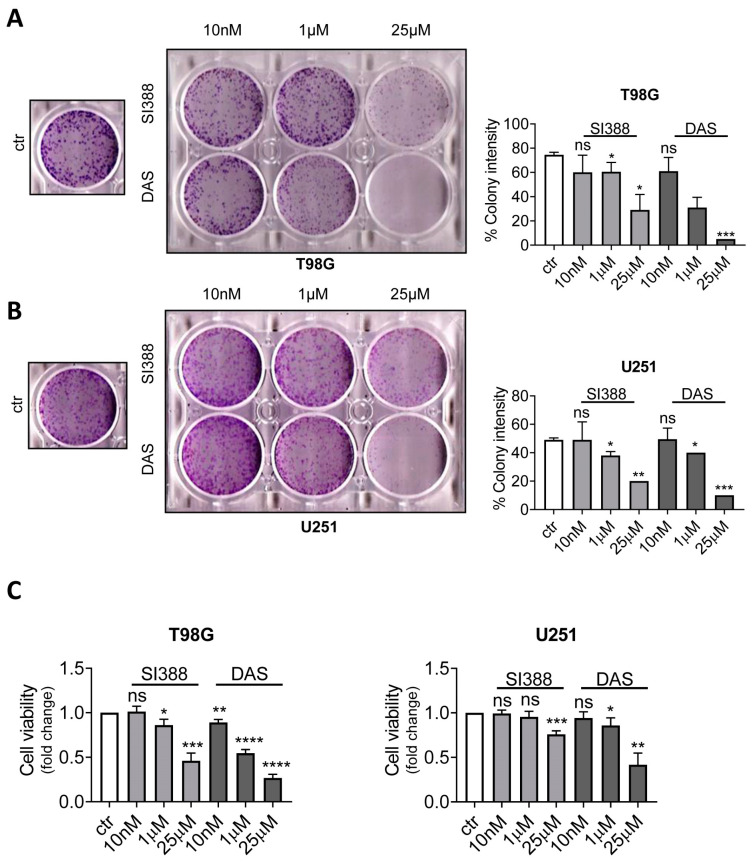
**SI388 inhibits cell proliferation and viability.** (**A**,**B**) Representative image (left) and relative quantification (right) of clonogenic assays performed on T98G and U251 cell lines treated with ascending concentrations of SI388 or dasatinib (DAS) for 24 h. DMSO was used as control. (**C**) Histogram representing the percentage of cell viability evaluated by MTS assay on T98G and U251 cell lines treated as in A for 72 h. Histograms summarize quantitative data of the mean ± S.E.M of three independent experiments. Statistical analyses: unpaired Student’s *t*-test: * *p* < 0.05; ** *p* < 0.01; *** *p* < 0.001; **** *p* < 0.0001; ns, not significant.

To further characterize the ability of SI388 to inhibit tumor growth, we performed clonogenic assays using T98G and U251 cell lines. Cells were starved and treated for 24 h with increasing concentration (10 nM, 1 µM, and 25 µM) of SI388 or DAS. The ability of single cells to proliferate and produce colonies was evaluated after 15 days, both in control conditions (treated with DMSO) and after treatment with different Src inhibitors. As shown in Figure 3 (panels A and B), both SI388 and DAS impaired cell growth and proliferation in a dose-dependent manner in both cell lines.

Next, the ability of SI388 to inhibit cell viability was evaluated using an MTS assay. As shown in Figure 3C, SI388 significantly impaired cell viability after 72 h of drug treatment in a dose-dependent manner both is T98G and in U251 cells.

#### 2.3.3. SI388 Inhibits Src Kinase Activity in GBM Patient-Derived Cancer Stem Cells and Reduces Their Ability to form Neurospheres

Recently, gene expression subtyping highlighted that the mesenchymal GBM subtype shows higher Src pathway activation, resulting in enhanced sensitivity to dasatinib treatment compared to other GBM subtypes [19].

For this reason, we next evaluated the ability of SI388 to affect cell growth in a patient-derived mesenchymal GBM cancer stem cells, named GBMSC83, cultured in non-adherent conditions to form neurospheres.

**Figure 4:**
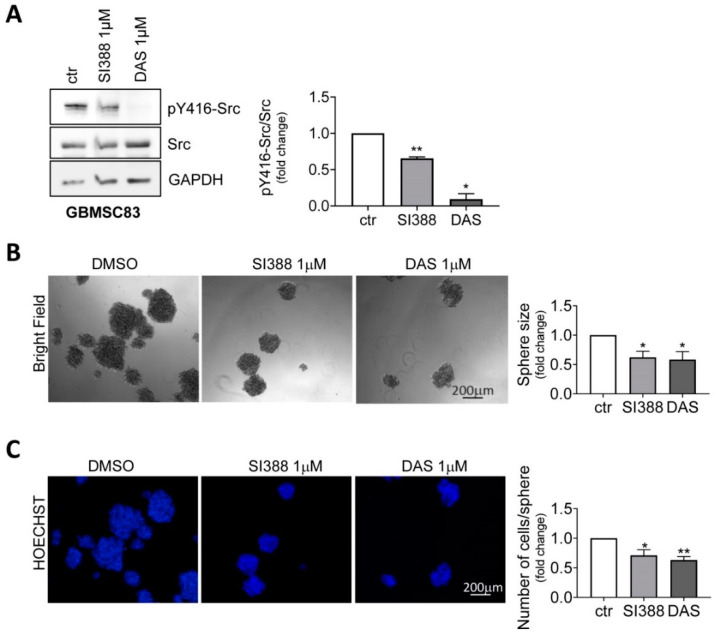
**SI388 inhibits Src activity in GBM stem cells and affects neurosphere size**. (**A**) Western blot analysis (left) and relative quantification (right) of pY416-Src in GBMSC83 cells treated with SI388 (1 µM) or dasatinib (DAS 1 µM) for 24 h. GAPDH was used as loading control. (**B**,**C**) Neurosphere assay analysis on GBMSC83 cells treated with SI388 1 µM or dasatinib (DAS) 1 µM for 72 h. (**B**) Brightfield images and histogram of mean neurosphere diameter (reported as fold change to control). (**C**) Hoechst staining and histogram of the number of cells per sphere (fold change to control). Histograms summarize quantitative data of the mean ± S.E.M. of three independent experiments. Statistical analyses: paired Student’s *t*-test: * *p* < 0.05; ** *p* < 0.01.

Firstly, we observed a significant reduction in phosphorylation of Src on Tyr416 upon a 1 µM SI388 treatment in this system; although, the inhibitory effect of SI388 is weaker compared to a 1 µM DAS treatment (Figure 4A). We then demonstrated that both treatments significantly decreased the growth of spheres, resulting in an overall reduced diameter of the spheres compared to the control condition (Figure 4B) and a reduced average number of cells per sphere (Figure 4C).

#### 2.3.4. SI388 Increases Cell Sensitivity to Ionizing Radiation (IR) Treatment

To further strengthen our findings, we next investigated if SI388 treatment could increase cell sensitivity to radiotherapy of GBM cells.

**Figure 5:**
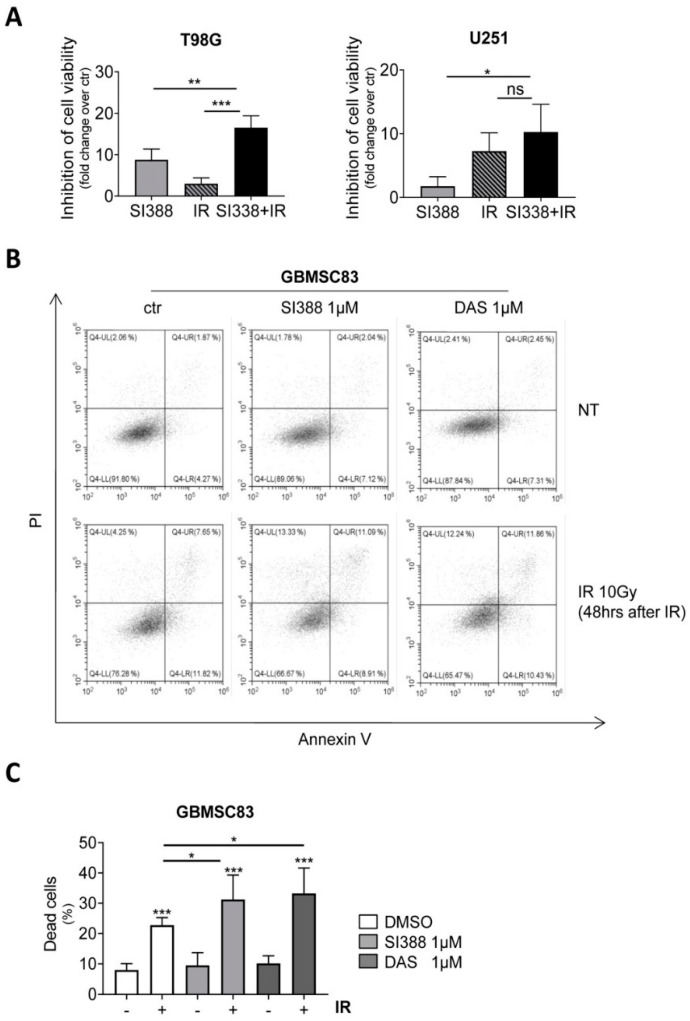
**SI388 increases cell sensitivity to IR**. (**A**) Histogram representing the percentage of the inhibition of cell viability evaluated by MTS assays after 48 h from IR (10Gy) on T98G and U251 cell lines with or without SI388 (1 µM). (**B**,**C**) Cytofluorimetric analysis of AnnexinV-PI (**B**) and relative histogram (**C**) showing percentage of dead GBMSC83 cells after 48 h from IR 10 Gy with or without SI388 (1 µM) or dasatinib (DAS, 1 µM). Histograms summarize quantitative data of the mean ± S.E.M. of four independent experiments. Statistical analyses: unpaired Student’s *t*-test: * *p* < 0.05; ** *p* < 0.01; *** *p* < 0.001; ns, not significant.

To this aim, T98G and U251 cells were pre-incubated or not with SI388 for 24 h and then irradiated (10 Gy). MTS assays revealed that SI388 pre-treatment increases the sensitivity to IR in both GBM cell lines; although, this result was only statistically significant in T98G cells (Figure 5A).

GBMSC83 cells were pretreated with 1 µM SI388 or 1 µM DAS, or DMSO as a control, for 24 h and then irradiated (10 Gy). After 48 h from IR, cytofluorimetric analysis of AnnexinV-PI staining was performed. Interestingly, SI388, as well as dasatinib, sensitized cells to IR and significantly increased the percentage of cell death compared to the control samples (Figure 5, panels B and C).

## 3. Materials and Methods

### 3.1. Chemistry

All commercially available chemicals were used as purchased. DCM was dried over calcium hydride. Anhydrous reactions were run under a positive pressure of dry N_2_ or argon. TLC was carried out using Merck TLC plates silica gel 60 F254. Chromatographic purifications were performed on columns packed with Merk 60 silica gel, 23–400 mesh, for flash technique. ^1^H NMR and ^13^C NMR spectra were recorded on a Brucker Avance DPX400 (at 400 MHz for ^1^H and 100 MHz for ^13^C) or using a Varian Gemini 200 (200 MHz for for ^1^H) in DMSO-d_6_, CDCl_3_, or acetone-d6 as solvents as indicated. Chemical shifts (δ) were expressed in parts per million (ppm) relative to tetramethylsilane (TMS), which was used as the internal standard. Data are shown as follows: chemical shift, multiplicity (s = singlet, d = doublet, t = triplet, q = quartet, quint = quintet, sx = sextet, dd = doublet of doublets, tt = triple of triplets, bs = broad singlet), coupling constant (*J*) in Hertz (Hz), and integration. Elemental analysis for C, H, N, and S was determined using Thermo Scientific Flash 2000 and results were within ±0.4% of the theoretical value. All target compounds possessed a purity of ≥95%, which was verified by elemental analysis. Microwave irradiation experiments were conducted using a CEM Discover synthesis unit (CEM Corp., Matthews, NC, USA). The machine consists of a continuous focused microwave power delivery system with operator selectable power output from 0 to 300 W. The temperature of the contents of the vessels was monitored using a calibrate infrared temperature control mounted under the reaction vessel. All of the experiments were performed using a stirring option whereby the contents of the vessel are stirred by means of a rotating magnetic plate located below the floor of the microwave cavity and a Teflon-coated magnetic stir bar in the vessel.

Synthesis of the intermediates **3a** [9], **3b** [15], **3c** [14], **3d** [8], **3e** and **4a,b** [9] has already been reported by us.

#### 3.1.1. General Procedure for the Synthesis of Final Compounds **2a-e**, **2h-j** and **2n-s**

A solution of the appropriate 4-chloro derivatives **3a-e, 8a-c, or 8e-h** (1 mmol) and the appropriate aniline (2 mmol) in absolute ethanol was refluxed for 5 h. After cooling, the solvent was evaporated under reduced pressure, and the crude was treated with water (20 mL), and then extracted with CH_2_Cl_2_ (20 mL). The organic phase was washed with water (20 mL), dried (MgSO_4_), and concentrated under reduced pressure. The obtained oil was crystallized by adding a mixture of diethyl ether/petroleum ether (bp 40–60 °C) (1:1) and standing in a refrigerator to afford a white solid, which was recrystallized from absolute ethanol.

1-(2-Chloro-2-phenylethyl)-*N*-(2-chlorophenyl)-6-(methylthio)-1*H*-pyrazolo[3,4-*d*]pyrimidin-4-amine **2a** (SI388). Yield: 69% (white solid). Mp: 169–171 °C. ^1^H NMR (400 MHz, DMSO-d_6_): δ 10.02 (s, 1H), 7.67 (br s, 1H), 7.58–7.56 (m, 2H), 7.49–7.47 (m, 2H), 7.41–7.30 (m, 5H), 5.64–5.60 (m, 1H), 4.89–4.83 and 4.76–4.71 (2m, 2H), 2.40 (s, 3H). ^13^C NMR (101 MHz, DMSO-d_6_): δ 169.67, 155.07, 153.98, 137.98, 134.71, 132.21, 131.67, 129.87, 129.09, 128.79, 127.79, 127.44, 126.41, 125.01, 98.57, 60.10, 53.78, 14.37. IR (KBr disc) cm^−1^: 3156 (NH). Elem. anal. calcd. for C_20_H_17_N_5_Cl_2_S: C 55.82, H 3.98, N 16.27, S 7.45; found: C 55.78, H 4.11, N 16.45, S 7.07.

1-(2-Chloro-2-phenylethyl)-*N*-(4-chlorophenyl)-6-(methylthio)-1*H*-pyrazolo[3,4-*d*]pyrimidin-4-amine **2b**. Yield: 71% (white solid). Analytical and spectroscopic data are in accordance with those previously reported by us [20].

1-(2-Chloro-2-phenylethyl)-6-(ethylthio)-*N*-phenyl-1*H*-pyrazolo[3,4-*d*]pyrimidin-4-amine **2c**. Yield: 51% (white solid). Mp: 128–131 °C. ^1^H NMR (400 MHz, CDCl_3_): δ 9.37 (br s, 1H), 7.49–7.40, 7.38–7.35 and 7.34–7.30 (3m, 11 H), 6.94 (br s, 1H), 5.43–5.40 (m, 1H), 4.87–4.81 and 4.71–4.44 (2m, 2H), 3.28–3.19 (q, 2H, *J* = 8.0 Hz), 1.46 (t, 3H, *J* = 8.0 Hz). ^13^C NMR (101 MHz, CDCl_3_): δ 153.98, 137.68, 136.15, 134.31, 130.01, 129.69, 129.37, 129.27, 128.90, 128.86, 127.48, 127.43, 127.31, 60.08, 53.83, 25.65, 14.25. IR (KBr disc) cm^−1^: 3357 (NH). Elem. anal. calcd. for C_21_H_20_N_5_ClS: C 61.53, H 4.92, N 17.08, S 7.82; found: C 61.31, H 4.66, N 17.23, S 8.02.

1-(2-Chloro-2-phenylethyl)-*N*-(3-chlorophenyl)-6-(isopropylthio)-1*H*-pyrazolo[3,4-*d*]pyrimidin-4-amine **2d**. Yield: 65% (white solid). Analytical and spectroscopic data are in accordance with those previously reported by us[20].

1-(2-Chloro-2-phenylethyl)-*N*-(2-chlorophenyl)-6-((2-morpholinoethyl)thio)-1*H*-pyrazolo[3,4-*d*]pyrimidin-4-amine **2e**. Yield: 62% (white solid). Mp: 117–118 °C. ^1^H NMR (400 MHz, CDCl_3_): δ 8.04 (br s, 1H), 7.52–7.46 (m, 3H), 7.37–7.32 (m, 6 H), 5.50–5.46 (m, 1H), 5.28–5.15 (m, 1H), 4.76–4.71 (m, 2H), 3.87 (br s, 4H), 3.37 (br s, 2H), 2.77 (br s, 6H). ^13^C NMR (101 MHz, CDCl_3_): δ 162.52, 154.67, 154.45, 137.71, 137.52, 137.35, 135.78, 134.10, 133.84, 133.37, 129.41, 129.02, 127.43, 126.54, 110.80, 66.20, 60.87, 57.28, 54.24, 53.15, 31.46. IR (KBr disc) cm^−1^: 3061 (NH). Elem. anal. calcd. for C_25_H_26_N_6_OSCl_2_: C 56.71, H 4.95, N 15.87, S 6.56; found: C 56.41, H 4.71, N 15.87, S 6.87.

1-(2-Chloro-2-phenylethyl)-6-ethyl-*N*-phenyl-1*H*-pyrazolo[3,4-*d*]pyrimidin-4-amine **2h**. Yield: 44% (white solid). Mp: 174–176 °C. ^1^H NMR (400 MHz, DMSO-d_6_): δ 9.92 (s, 1H), 8.17 (br s, 1H), 7.85 (d, 2H, *J* = 7.6 Hz), 7.47 (dd, 2H, *J* = 8.0 Hz, *J* = 1.6 Hz), 7.35–7.30 (m, 5H), 7.05 (t, 1H, *J* = 7.2 Hz), 5.67–5.63 (m, 1H), 4.96–4.90 and 4.79–4.72 (2m, 2H), 2.75 (q, 2H, *J* = 7.6 Hz), 1.27 (t, 3H, *J* = 7.6 Hz). ^13^C NMR (400 MHz, DMSO-d_6_): δ 169.29, 155.14, 154.56, 139.92, 138.51, 132.72, 129.47, 129.23, 129.18, 128.12, 123.66, 121.42, 99.70, 61.04, 52.96, 32.72, 13.13. IR (KBr disc) cm^−1^: 3155 (NH). Elem. anal. calcd. for C_21_H_20_N_5_Cl: C 66.75, H 5.33, N 18.53; found: C 66.92, H 5.41, N 18.64.

1-(2-Chloro-2-phenylethyl)-*N*-(3-chlorophenyl)-6-cyclopentyl-1*H*-pyrazolo[3,4-*d*]pyrimidin-4-amine **2i**. Yield: 41% (white solid). Mp: 189–190 °C. ^1^H (400 MHz, DMSO-d_6_): δ 10.08 (s, 1H,), 8.33 (t, 1H, *J* = 2.0 Hz), 7.63 (dd, 1H, *J* = 8.4 Hz, *J* = 1.2 Hz), 7.46 (dd, 2H, *J* = 8.0 Hz, *J* = 1.6 Hz), 7.37–7.28 (m, 4H), 7.07 (dd, 1H, *J* = 7.2 Hz, *J* = 1.2 Hz), 5.66–5.63 (m, 1H), 4.95–4.90 and 4.82–4.77 (2m 2H), 3.21 (quint, 1H, *J* = 8.0 Hz), 2.02–1.96, 1.93–1.82, 1.79–1.78 and 1.65–1.62 (4m, 8H). ^13^C NMR (101 MHz, DMSO-d_6_): δ 171.91, 154.96, 154.25, 141.57, 138.45, 133.51, 132.61, 130.77, 129.46, 129.16, 128.10, 122.87, 120.44, 118.92, 99.94, 60.97, 52.96, 48.56, 33.06, 32.91, 26.39. IR (KBr disc) cm^−1^: 3068 (NH). Elem. anal. calcd. For C_24_H_23_N_5_Cl_2_: C 63.72, H 5.12, N 15.48; found: C 63.48, H 5.34, N 15.61.

1-(2-Chloro-2-phenylethyl)-6-cyclohexyl-N-phenyl-1*H*-pyrazolo[3,4-*d*]pyrimidin-4-amine **2j**. Yield: 44% (white solid). Mp: 134–136 °C. ^1^H NMR (400 MHz, CDCl_3_): δ 7.46–7.45 (m, 4H), 7.40–7.36 (m, 4H), 7.32–7.26 (m, 3H), 7.03 (br s, 1H), 5.48–5.45 (m, 1H), 4.92–4.87 and 4.82–4.77 (2m, 2H), 2.79 (tt, 1H, *J* = 12.0 Hz, *J* = 3.6 Hz), 2.04–2.01 (m, 2H), 1.89–1.86 (m, 2H), 1.77–1.65 (m, 3H), 1.47–1.31 (m, 3H). ^13^C NMR (101 MHz, CDCl_3_): δ 205.15, 154.56, 137.78, 133.40, 129.88, 129.59, 129.10, 128.77, 128.73, 127.49, 125.28, 98.09, 60.04, 53.66, 47.08, 46.23, 39.33, 31.53, 26.10, 25.97. IR (KBr disc) cm^−1^: 3361 (NH). Elem. anal. calcd. For C_25_H_26_N_5_Cl: C 69.51, H 16.21, N 6.07; found: C 69.72, H 15.82, N 15.87.

1-(2-Chloro-2-phenylethyl)-*N*,6-diphenyl-1*H*-pyrazolo[3,4-*d*]pyrimidin-4-amine **2n**. Yield: 72% (white solid). Mp: 130–140 °C (dec). ^1^H NMR (400 MHz, CDCl_3_): δ 8.52–8.50 (m, 2H), 7.54–7.43 (m, 10H), 7.38–7.26 (m, 4H), 5.55–5.52 (m, 1H), 5.04–4.98 and 4.89–4.84 (2m 2H). ^13^C NMR (101 MHz, CDCl_3_): δ 170.71, 170.09, 155.20, 145.56, 139.95, 135.55, 129.47, 129.01, 128.87, 128.85, 128.00, 127.53, 126.57, 126.37, 121.84, 117.02, 114.39, 59.58, 53.41. IR (KBr disc) cm^−1^: 3347 (NH). Elem. anal. calcd. For C_25_H_20_N_5_Cl: C 70.50, H 4.73, N 16.44; found: C 70.63, H 4.89, N 16.43.

1-(2-Chloro-2-phenylethyl)-*N*-(3-chlorophenyl)-6-phenyl-1*H*-pyrazolo[3,4-*d*]pyrimidin-4-amine **2o**. Yield: 62% (white solid). Mp: 82–84 °C. ^1^H NMR (400 MHz, DMSO-d_6_): δ 10.26 (s, 1H), 8.43–8.40 (m, 2H), 8.31 (s, 1H), 8.25 (t, 1H, *J* = 2.0 Hz), 7.77–7.74 (m, 1H), 7.55–7.50 (m, 5H), 7.43 (t, 1H, *J* = 8.0 Hz), 7.35–7.25 (m, 3H), 7.16–7.14 (m, 1H), 5.73–5.70 (m, 1H), 5.09–5.06 and 4.9489–4.88 (2m 2H). ^13^C NMR (101 MHz, DMSO-d_6_): δ 161.19, 155.27, 154.29, 141.27, 138.51, 138.26, 133.55, 132.94, 131.27, 130.99, 129.51, 129.19, 129.08, 128.69, 128.19, 123.30, 120.88, 119.46, 100.40, 61.09, 53.14. IR (KBr disc) cm^−1^: 3291 (NH). Elem. anal. calcd. For C_25_H_19_N_5_Cl_2_: C 65.22, H 4.16, N 15.21; found: C 65.36, H 4.42, N 14.97.

1-(2-Chloro-2-(4-fluorophenyl)ethyl)-6-(methylthio)-*N*-phenyl-1*H*-pyrazolo[3,4-*d*]pyrimidin-4-amine **2p**. Yield: 47% (white solid). Mp: 227–230 °C. ^1^H NMR (400 MHz, DMSO-d_6_): δ 10.27 (s, 1H), 8.26 (br s, 1H), 7.78 (d, 2H, *J* = 8.0 Hz), 7.54–7.51 (m, 2H), 7.34 (t, 2H, *J* = 7.6 Hz), 7.17–7.13 (m, 2H), 7.09–7.06 (m, 1H) 5.67–5.63 (m, 1H), 4.90–4.84 and 4.77–4.72 (2m, 2H), 2.50 (s, 3H). ^13^C NMR (101 MHz, DMSO-d_6_): δ NMR (101 MHz), δ 169.17, 163.88, 161.44, 158.93, 154.61, 139.35, 134.88, 134.85, 133.55, 130.49, 130.41, 129.21, 116.14, 115.92, 99.03, 60.06, 53.06, 14.26. IR (KBr disc) cm^−1^: 3093 (NH). Elem. anal. calcd. For: C 58.04, H 4.14, N 16.92, S 7.75; found: C 58.09, H 4.07, N 15.75, S 8.33.

1-(2-Chloro-2-(4-chlorophenyl)ethyl)-6-methyl-*N*-phenyl-1*H*-pyrazolo[3,4-*d*]pyrimidin-4-amine **2q**. Yield: 50% (white solid). Mp: 202–203 °C. ^1^H NMR (400 MHz, DMSO-d_6_): δ 9.92 (s, 1H), 8.14 (br s, 1H), 7.82 (d, 2H, *J* = 8.0 Hz), 7.52–7.49 (m, 2H), 7.41–7.33 (m, 4H), 7.06 (t, 2H, *J* = 7.6 Hz), 5.67–5.64 (m, 1H), 4.95–4.89 and 4.76–4.71 (2m, 2H), 2.48 (s, 3H). ^13^C NMR (101 MHz, DMSO-d_6_): δ 165.25, 155.19, 154.52, 139.81, 137.61, 134.02, 132.80, 130.15, 129.26, 129.18, 123.87, 121.54, 99.48, 60.02, 52.80, 26.62. IR (KBr disc) cm^−1^: 3153 (NH). Elem. anal. calcd. For C_20_H_17_N_5_Cl_2_: C 60.31, H 4.30, N 17.58; found: C 60.51, H 4.60, N 17.40.

1-(2-Chloro-2-(4-chlorophenyl)ethyl)-6-ethyl-*N*-phenyl-1*H*-pyrazolo[3,4-*d*]pyrimidin-4-amine **2r**. Yield: 39% (white solid). Mp: 193–195 °C. ^1^H NMR (400 MHz, CDCl_3_): δ 7.68 (s, 1H), 7.54–7.45 (m, 4H), 7.42–7.40 (m, 2H), 7.41–7.38 (m, 2H), 7.35–7.30 (m, 5H), 5.38–5.34 (m, 1H), 4.90–4.84 and 4.76–4.69 (2m, 2H), 2.99 (q, 2H, *J* = 7.6 Hz), 1.45 (t, 3H, *J* = 7.6 Hz). ^13^C NMR (101 MHz, CDCl_3_): δ 155.56, 155.19, 137.42, 136.51, 134.86, 132.94, 129.76, 128.93, 128.92, 127.16, 125.25, 98.28, 59.10, 53.39, 40.77, 21.76, 14.01. IR (KBr disc) cm^−1^: 3148 (NH). Elem. anal. calcd. For C_21_H_19_N_5_Cl_2_: C 61.17, H 4.64, N 16.99; found: C 61.48, H 4.77, N 16.65.

1-(2-Chloro-2-(4-chlorophenyl)ethyl)-*N*-phenyl-6-propyl-1*H*-pyrazolo[3,4-*d*]pyrimidin-4-amine **2s**. Yield: 34% (white solid). Mp: 154–156 °C. ^1^H NMR (400 MHz, CDCl_3_): δ 7.68 (br s, 1H), 7.54–7.51 (m, 4H), 7.42–7.40 (m, 2H), 7.34–7.27 (m, 4H), 5.39–5.35 (m, 1H), 4.89–4.81 and 4.74–4.69 (2m, 2H), 2.90 (t, 2H, *J* = 7.6 Hz), 1.94 (sx, 2H, *J* = 7.6 Hz), 1.05 (t, 2H, *J* = 7.6 Hz). ^13^C NMR (101 MHz, CDCl_3_): δ 136.35, 135.95, 135.24, 133.55, 130.35, 129.60, 129.13, 128.95, 128.89, 128.82, 126.40, 58.98, 53.67, 40.90, 38.13, 21.47, 21.05, 13.77. IR (KBr disc) cm^−1^: 3161 (NH). Elem. anal. calcd. For C_22_H_21_N_5_Cl_2_: C 61.98, H 4.96, N 16.43; found: C 62.14, H 5.04, N 16.49.

#### 3.1.2. General Procedure for the Synthesis of Final Compounds **2f,g**

The appropriate 3-aminophenol derivative (5 mmol) was added to a solution of 4-chloro-1-(2-chloro-2-phenylethyl)-6-[(2-morpholinoethyl)thio]-1H-pyrazolo[3,4-d]pyrimidine **3d** (1 mmol, 438 mg) in absolute ethanol (10 mL), and the mixture was refluxed for 3−5 h. After cooling to room temperature, the solvent was evaporated under reduced pressure and the crude was solved in ethyl acetate (10 mL), washed with 0.1 N HCl solution (2 × 10 mL), 1 N NaOH solution (10 mL) and brine (2 × 10 mL), dried (MgSO4), filtered, and concentrated under reduced pressure to give a brown oil which crystallized by adding a 1:1 mixture of diethyl ether/petroleum ether (bp 40−60 °C) affording yellow solid.

5-({1-(2-Chloro-2-phenylethyl)-6-[(2-morpholinoethyl)thio]-1H-pyrazolo[3,4-*d*]pyrimidin-4-yl)amino)-2-fluorophenol hydrochloride **2f**. Yield: 38% (yellow solid). Mp: 238–239 °C. ^1^H NMR (200 MHz, DMSO-d_6_): δ 10.23 (s, 1H), 10.18 (br s, 1H), 7.63–7.61, 7.41–7.39, and 7.19–7.15 (3m, 9H), 5.68–5.62 (m, 1H), 5.21–5.07 and 4.83–4.78 (2m, 2H), 3.89–3.18 (m, 12H). ^13^C NMR (50 MHz, DMSO-d_6_): δ 167.84, 156.93, 152.04, 151.75, 150.39, 138.70, 138.23, 134.50, 129.39, 128.86, 127.54, 117.51, 113.09, 107.78, 106.14, 66.74, 61.80, 54.23, 52.66, 45.49, 31.87. IR (KBr disc) cm^−1^: 3300–3100 (OH and NH). (NH). Elem. anal. calcd. For C_25_H_27_N_6_O_2_SCl_2_F: C 53.10, H 4.81, N 14.26, S 5.67; found: C 53.25, H 4.77, N 14.49, S 5.27.

5-((1-(2-Chloro-2-phenylethyl)-6-((2-morpholinoethyl)thio)-1*H*-pyrazolo[3,4-*d*]pyrimidin-4-yl)amino)-2,4-difluorophenol **2g**. Yield: 25% (yellow solid). Mp: 146–149 °C. ^1^H NMR (400 MHz, CDCl_3_): δ 8.33 (br s, 1H), 7.89 (br s, 1H), 7.47–7.46 (m, 2H), 7.28–7.27 (m, 4H), 6.98–6.96 (m, 1H), 6.98–6.92 (m, 1H), 5.56–5.52 (m, 1H), 4.99–4.92 and 4.753–4.69 (2m, 2H), 3.92–3.91 (br s, 4H), 3.42 (br s, 2H), 3.98 (br s, 2H), 3.73 (br s, 4H). ^13^C NMR (101 MHz, CDCl_3_) δ 168.21, 157.49, 151.95, 151.03, 148.20, 146.02, 138.23, 134.50, 132.47, 129.39, 128.86, 127.54, 109.07, 106.92, 105.42, 66.82, 61.80, 54.23, 53.16, 51.43, 31.44. IR (KBr disc) cm^−1^: 3420–3300 (OH and NH). Elem. anal. calcd. For C_25_H_25_N_6_O_2_SClF_2_: C 54.89, H 4.61, N 15.36, S 5.86; found: C 55.00, H 4.70, N 15.47, S 5.67.

#### 3.1.3. General Procedure for the Synthesis of Final Compounds **2k-m**

The opportune amine (2.1 mmol) was added to a solution of 4-chloro-1-(2-chloro-2-phenylethyl)-6-(trifluoromethyl)-1*H*-pyrazolo[3,4-*d*]pyrimidine 8d (0.25 g, 0.69 mmol) in absolute ethanol (4 mL). The reaction mixture was irradiated in the microwave for 2–10 min at 80 °C in an open vessel. After cooling, the solvent was evaporated under reduced pressure, and the crude was treated with water (5 mL), then extracted with CH_2_Cl_2_ (5 mL); the organic phase was washed with water (5 mL), dried (MgSO_4_), and concentrated under reduced pressure. The obtained oil was crystallized by adding a mixture of CH_2_Cl_2_/n-hexane (1:4) and standing in a refrigerator to afford the pure product as a white solid.

1-(2-Chloro-2-phenylethyl)-*N*-phenyl-6-(trifluoromethyl)-1*H*-pyrazolo[3,4-*d*]pyrimidin-4-amine **2k**. Yield: 65% (white solid). Mp: 139–141 °C. ^1^H NMR (400 MHz, CDCl_3_): δ 7.53–7.38 (m, 8H), 7.33–7.29 (m, 3H), 5.46–5.42 (m, 1H), 5.00–4.94 and 4.84–4.79 (2m, 2H). ^13^C NMR (101 MHz, CDCl_3_): 155.95, 149.62, 141.42, 137.40, 133.90, 130.18, 129.90, 129.29, 128.87, 127.46, 123.84, 123.18, 110.63, 59.97, 54.15.. IR (KBr disc) cm^−1^: 3235 (NH). Elem. anal. calcd. For C_20_H_15_N_5_ClF_3_: C 57.49, H 3.92, N 16.76; found: C 57.44, H 4.15, N 16.43.

1-(2-Chloro-2-phenylethyl)-*N*-(3-fluorophenyl)-6-(trifluoromethyl)-1*H*-pyrazolo[3,4-*d*]pyrimidin-4-amine **2l**. Yield: 13% (white solid). Mp: 164–166 °C. ^1^H NMR (400 MHz, CDCl_3_): δ 8.63 (br s 1H), 7.51 (br s, 1H), 7.42–7.26 (m, 8H), 6.96 (t, 1H, J = 7.6 Hz), 5.51–5.48 (m, 1H), 5.02–4.97 and 4.87–4.82 (2m, 2H). ^13^C NMR (101 MHz, CDCl_3_): δ NMR (101 MHz, ) δ 164.34, 161.88, 154.08, 139.30, 138.04, 137.59, 136.35, 135.48, 132.94, 132.69, 130.66, 130.57, 129.18, 128.82, 127.49, 127.48 60.07, 54.06. IR (KBr disc) cm^−1^: 3281 (NH). Elem. anal. calcd. For C_20_H_14_N_5_ClF_4_: C 55.12, H 3.24, N 16.07; found: C 55.36, H 3.34, N 15.89.

*N*-(3-Chlorophenyl)-1-(2-chloro-2-phenylethyl)-6-trifluoromethyl-1*H*-pyrazolo[3,4-*d*]pyrimidin-4-amine **2m**. Yield: 13% (white solid). Mp: 88–90 °C. ^1^H NMR (400 MHz, CDCl_3_): δ 8.18 (br s, 1H), 7.58 (br s, 1H), 7.43–7.39 (m, 5H), 7.34–7.28 (m, 4H), 5.49–5.45 (m, 1H), 5.03–4.97 and 4.87–4.82 (2m, 2H). NMR (101 MHz, CDCl_3_): δ 147.88, 145.37, 137.41, 135.50, 134.72, 132.79, 130.89, 130.67, 129.48, 129.28, 128.90, 128.87, 127.91, 127.47, 127.45, 127.43, 59.98, 54.19. IR (KBr disc) cm^−1^: 3308 (NH). Elem. anal. calcd. For C_20_H_14_N_5_Cl_2_F_3_: C 53.11, H 3.12, N 15.49; found: C 53.12, H 3.15, N 15.46.

#### 3.1.4. General Procedure for the Synthesis of Intermediates **5a,b**

Ethoxymethylenemalononitrile (2.44 g, 20 mmol) was added to a solution of the opportune 2-hydrazino-1-phenylethanol **4a,b** (20 mmol) in absolute ethanol (40 mL) and the reaction mixture was refluxed for 6 h. The solvent was concentrated to 50% of the initial volume and cooled. The yellow solid obtained was filtered and recrystallized from absolute ethanol.

5-Amino-1-(2-hydroxy-2-phenylethyl)-1*H*-pyrazole-4-carbonitrile **5a**. Yield: 71% (white solid). Analytical and spectroscopic data are in accordance with those previously reported by us [20].

5-Amino-1-[2-(4-chlorophenyl)-2-hydroxyethyl]-1*H*-pyrazole-4-carbonitrile **5b**. Yield: 51% (white solid). Mp: 218–220 °C. ^1^H NMR (400 MHz, DMSO-d_6_): δ 7.50 (s, 1H), 7.36–7.31 (m, 4H), 6.41 (br s, 2H), 5.72 (d, 1H, *J* = 4.8 Hz), 4.92–4.88 (m, 1H), 4.05–4.00 and 3.95–3.90 (2m, 2H). ^13^C NMR (101 MHz, DMSO-d_6_): δ 152.62, 141.93, 140.63, 132.43, 128.69, 128.54, 115.86, 72.63, 71.01, 54.37. IR (KBr disc) cm^−1^: 3401–3287 (OH, NH_2_), 2217 (CN). Elem. anal. calcd. For C_12_H_11_N_4_ClO: C 54.87, H 4.22, N 21.33; found: C55.01, H 4.23, N 21.42.

#### 3.1.5. General Procedure for the Synthesis of Intermediates **6a,b**

2M NaOH (10 mL) was added to a solution of the opportune 5-amino-1-(2-hydroxy-2-phenylethyl)-1H-pyrazole-4-carboxamide derivative **5a,b** (10 mmol) in absolute ethanol (10 mL), and the mixture was refluxed for 2 h. After cooling, the solid obtained was filtered, washed with water and recrystallized from ethanol.

5-Amino-1-(2-hydroxy-2-phenylethyl)-1*H*-pyrazole-4-carboxamide **6a**. Yield: 66% (white solid). Analytical and spectroscopic data are in accordance with those previously reported by us [20].

5-Amino-1-[2-(4-chloro-phenyl)-2-hydroxyethyl]-1*H*-pyrazole-4-carboxamide **6b**. Yield: 58% (white solid). Mp: 215–216 °C. ^1^H NMR (400 MHz, DMSO-d_6_): δ 7.59 (s, 1H), 7.36–7.31 (m, 4H), 6.05 (br s, 2H), 5.79 (br s, 1H), 4.93–4.90 (m, 1H), 4.00–3.89 (m, 2H). IR (KBr disc) cm^−1^: 3450–3200 (OH + NH), 1622 (CO). ^13^C NMR (101 MHz, DMSO-d_6_): δ 166.67, 150.51, 142.20, 137.67, 132.31, 128.62, 128.53, 97.27, 71.31, 54.16. Elem. anal. calcd. For C_12_H_13_N_4_O_2_Cl: C 51.34, H 4.67, N 19.96; found: C 51.41, H 4.87, N 19.98.

#### 3.1.6. General Procedure for the Synthesis of 6-Substituted 1-(2-Hydroxy-2-Phenylethyl)-1,5-Dihydro-4*H*-Pyrazolo[3,4-*d*]Pyrimidin-4-one Derivatives **7a-h**

A solution of sodium ethoxide prepared from sodium (1.38 g, 60 mmol) and absolute ethanol (30 mL) and the appropriate alkyl ester (60 mmol) were added to a solution of the appropriate 5-amino-1-(2-hydroxy-2-phenylethyl)-1H-pyrazole-4-carboxamide 6a,b (10 mmol) in absolute ethanol (90 mL). The mixture was refluxed for 6–12 h; after cooling, ice water was added, and the solution was acidified with 3% acetic acid. The obtained precipitate was filtered, washed with water, and recrystallized from absolute ethanol to afford desired compounds 7a-h.

6-Ethyl-1-(2-hydroxy-2-phenylethyl)-1,5-dihydro-4*H*-pyrazolo[3,4-*d*]pyrimidin-4-one **7a**. Alkyl ester used for the synthesis: methyl propionate. Yield: 65% (white solid). Mp: 221–222 °C. ^1^H NMR (400 MHz, DMSO-d_6_): δ 11.90 (br s, 1H), 7.94 (s, 1H), 7.26–7.21 (m, 4H), 7.20–7.16 (m, 1H), 5.59 (br s, 1H), 5.05 (br s, 1H), 4.42–4.37 and 4.28–4.23 (2m, 2H), 2.54 (q, 2H, *J* = 7.6 Hz), 1.14 (t, 3H, *J* = 7.6 Hz). ^13^C NMR (101 MHz, DMSO-d_6_): δ 161.85, 158.59, 153.39, 143.01, 134.53, 128.55, 127.89, 126.54, 104.27, 71.66, 54.48, 28.00, 11.87. IR (KBr disc) cm^−1^: 3410 (NH), 3350–2990 (OH), 1661 (CO). Elem. anal. calcd. for C_15_H_16_N_4_O_2_: C 63.37, H 5.67, N 19.71; found: C 63.15, H 5.91, N 19.59.

6-Cyclopentyl-1-(2-hydroxy-2-phenylethyl)-1,5-dihydro-4*H*-pyrazolo[3,4-*d*]pyrimidin-4-one **7b**. Alkyl ester used for the synthesis: methyl cyclopentancarboxylate. Yield: 44% (white solid). Mp: 227–228 °C. ^1^H NMR (200 MHz, CDCl_3_): δ 10.95 (br s, 1H), 7.99 (s, 1H), 7.34–7.32, 7.29–7.25 and 7.22–7.17 (3m, 5H), 5.16–5.14 (m, 1H), 4.61–4.56 and 4.50–4.44 (2m, 2H), 4.33 (br s, 1H), 3.04 (t, 1H, *J* = 7.0 Hz), 2.06–2.04 (m, 2H), 1.88–1.1.79 (m, 4H), 1.71–1.66 (m, 2H). IR (KBr disc) cm^−1^: 3300–2950 (NH + OH), 1698 (CO). Elem. anal. calcd. for C_18_H_20_N_4_O_2_: C 66.65, H 6.21, N 17.27; found: C 66.84, H 6.29, N 17.31.

6-Cyclohexyl-1-(2-hydroxy-2-phenylethyl)-1,5-dihydro-4*H*-pyrazolo[3,4-*d*]pyrimidin-4-one **7c**. Alkyl ester used for the synthesis: methyl cyclohexane carboxylate. Yield: 68% (white solid). Mp: 216–218 °C. ^1^H NMR (400 MHz, DMSO-d_6_): δ 7.92 (s, 1H), 7.24–7.16 (m, 5H), 5.64 (br s, 1H), 5.07–5.03 (m, 1H), 4.42–4.36 and 4.31–4.26 (2m, 2H), 2.55–2.50 (m, 1H), 1.80–1.62 (m, 4H), 1.66–1.62 (m, 1H), 1.52–1.39 (m, 2H), and 1.29–1.13 (m, 3H). ^13^C NMR (101 MHz, DMSO-d_6_): δ 164.31, 158.78, 153.31, 142.97, 134.46, 128.50, 127.86, 126.54, 104.35, 71.68, 54.43, 42.83, 30.73, 30.69, 25.87, 25.81. IR (KBr disc) cm^−1^: 3330–3000 (NH + OH), 1701 (CO). Elem. anal. calcd. for C_19_H_22_N_4_O_2_: C 67.44, H 6.55, N 16.56; found C 67.58, H 8.82, N 16.44.

1-(2-Hydroxy-2-phenylethyl)-6-(trifluoromethyl)-1,5-dihydro-4*H*-pyrazolo[3,4-*d*]pyrimidin-4-one **7d**. Alkyl ester used for the synthesis: methyl trifluoroacetate. Yield: 58% (white solid). Mp: 212–214 °C. ^1^H NMR (200 MHz, DMSO-d_6_): δ 13.58 (br s, 1H), 8.23 (s, 1H), 7.38–7.19 (m, 5H), 5.71 (d, 1*H*, *J* = 6.1 Hz), 5.13–5.06 (m, 1H), 4.58–4.27 (m, 2H). IR (KBr disc) cm^−1^: 3450–2798 (OH + NH), 1704 (CO). Elem. anal. calcd. for C_14_H_11_N_4_O_2_F_3_: C 51.86, H 3.42, N 17.58; found: C 51.78, H 3.77, N 17.34.

1-(2-Hydroxy-2-phenylethyl)-6-phenyl-1,5-dihydro-4*H*-pyrazolo[3,4-*d*]pyrimidin-4-one **7e**. Alkyl ester used for the synthesis: methyl benzoate. Yield: 53% (white solid). Mp: 252–254 °C. ^1^H NMR (200 MHz, DMSO-d_6_): δ 12.36 (br s, 1H), 8.12–8.11, 7.60–7.58 and 7.34–7.31 (3m, 11H), 5.61 (d, 1H, *J* = 4.7 Hz), 5.18–5.06 (m, 1H), 4.61–4.28 (m, 2H). IR (KBr disc) cm^−1^: 3445–2977 (OH + NH), 1695 (CO). Elem. anal. calcd. for C_19_H_16_N_4_O_2_: C 68.66, H 4.85, N 16.86; found: C 68.38, H 5.09, N 17.02.

1-[2-(4-Chlorophenyl)-2-hydroxyethyl]-6-methyl-1,5-dihydro-4*H*-pyrazolo[3,4-*d*]pyrimidin-4-one **7f**. Alkyl ester used for the synthesis: ethyl acetate. Yield: 72% (white solid). Mp: 264–266 °C. ^1^H NMR (200 MHz, DMSO-d_6_): δ 8.00 (s, 1H), 7.39–7.27 (m, 4H), 5.75 (d, 1H, *J* = 4.8 Hz), 5.17–5.04 (m, 1H), 4.43–4.39 and 4.30–4.19 (2m, 2H), 2.33 (s, 3H). IR (KBr disc) cm^−1^: 3230–2950 (NH + OH), 1677 (CO). Elem. anal. calcd. for C_14_H_13_N_4_ClO_2_: C 55.18, H 4.30, N 18.39; found: C 55.26, H 4.59, N 18.26.

1-[2-(4-Chlorophenyl)-2-hydroxyethyl]-6-ethyl-1,5-dihydro-4*H*-pyrazolo[3,4-*d*]pyrimidin-4-one **7g**. Alkyl ester used for the synthesis: methyl propionate. Yield: 71% (white solid). Mp: 243–245 °C. ^1^H NMR (200 MHz, DMSO-d_6_): δ 12.10 (br s, 1H), 8.14 (s, 1H), 7.48–7.35 (m, 4H), 5.39 (br s, 1H), 5.23–5.19 (m, 1H), 4.64–4.41 (m, 2H), 2.71 (q, 2H, *J* = 7.8 Hz), 1.31 (t, 3H, *J* = 7.8 Hz). IR (KBr disc) cm^−1^: 3400–2900 (OH + NH), 1693 (CO). Elem. anal. calcd. for C_15_H_15_N_4_ClO_2_: C 56.52, H 4.74, N 17.58; found: C 56.78, H 4.94; N 17.60.

1-[2-(4-Chlorophenyl)-2-hydroxyethyl]-6-propyl-1,5-dihydro-4*H*-pyrazolo[3,4-*d*]pyrimidin-4-one **7h**. Alkyl ester used for the synthesis: methyl butirate. Yield: 80% (white solid). Mp: 257–259 °C. ^1^H NMR (200 MHz, DMSO-d_6_): δ 8.00 (s, 1H), 7.34–7.18 (m, 4H), 5.80 (d, 1H, *J* = 4.6 Hz), 5.17–5.03 (m, 1H), 4.43–4.21 (m, 2H), 1.65 (sx, 2H, *J* = 7.4 Hz), 0.88 (t, 3H, *J* = 7.4 Hz). IR (KBr disc) cm^−1^: 3440–2900 (NH + OH), 1694 (CO). Elem. anal. calcd. for C_16_H_17_N_4_O_2_Cl: C 57.75, H 5.15, N 16.84; found: C 58.07, H 5.48; N 16.98.

#### 3.1.7. General Procedure for the Synthesis of 6-Alkyl or 6-Phenyl 4-Chloro-1-(2-Chloro-2-Phenylethyl)-1*H*-Pyrazolo[3,4-*d*]pyrimidines **8a-h**

The Vilsmeier complex, previously prepared from POCl_3_ (4.60 g, 30 mmol) and anhydrous DMF (2.20 g, 30 mmol), was added to a suspension of the appropriate intermediate **7a-h** (1 mmol) in CHCl_3_ (10 mL). The mixture was refluxed for 6–12 h. The solution was washed with water (2 × 20 mL), dried (MgSO_4_), filtered, and concentrated under reduced pressure. The crude oil was purified by column chromatography (Florisil, 100–200 mesh), using diethyl ether as eluant, to afford compounds **8a-c, f-h** as yellow or colorless oils. Compounds **8d,e** crystallized standing in a refrigerator by adding a 1:1 mixture of diethyl ether/petroleum ether (bp 40– 60 °C) (1:1) as white solids.

4-Chloro-1-(2-chloro-2-phenylethyl)-6-ethyl-1*H*-pyrazolo[3,4-*d*]pyrimidine **8a**. Yield: 85% (colorless oil). ^1^H NMR (400 MHz, CDCl_3_): δ 11.29 (br s, 1H), 8.05 (s, 1H), 7.40–7.37 (m, 2H), 7.32–7.25 (m, 3H), 5.49–5.46 (m, 1H), 4.92–4.86 and 4.78–4.72 (2m, 2H), 2.74 (q, 2H, *J* = 7.2 Hz), 1.35 (t, 3H, *J* = 7.2 Hz). ^13^C NMR (101 MHz, CDCl_3_): 161.18, 160.03, 153.79, 137.86, 135.47, 129.04, 128.75, 127.47, 103.99, 60.19, 54.01, 28.56, 11.29. Elem. anal. calcd. for C_15_H_14_N_4_Cl_2_: C 56.09, H 4.39, N 17.44; found: C 56.18, H 4.51, N 17.63.

4-Chloro-1-(2-chloro-2-phenylethyl)-6-cyclopentyl-1*H*-pyrazolo[3,4-*d*]pyrimidine **8b**.Yield: 95% (yellow oil). ^1^H NMR (200 MHz, CDCl_3_): δ 8.04 (s, 1H), 7.39–7.24 (m, 5H), 5.28–5.21 (m, 1H), 4.67–4.59 and 4.54–4.43 (2m, 2H), 3.12–3.02 (m, 1H), 1.87–1.43 (m, 8H). Elem. anal. calcd. for C_18_H_18_N_4_Cl_2_: C 58.84, H 5.02, N 15.51; found: C 60.00, H 4.99, N 15.21.

4-Chloro-1-(2-chloro-2-phenylethyl)-6-cyclohexyl-1*H*-pyrazolo[3,4-]pyrimidine 8c. Yield: 98% (yellow oil). ^1^H NMR (200 MHz, DMSO-d_6_): δ 8.45 (s, 1H), 7.48–7.42 and 7.35–7.33 (2m, 5H), 5.78–5.67 (m, 1H), 5.15–4.96 (m, 2H), 2.88 (t, 1H, *J* = 7.8 Hz), 2.00–1.94 and 1.94–1.33 (2m, 10H). Elem. anal. calcd. for C_19_H_20_N_4_Cl_2_: C 60.81, H 5.37, N 14.93; found: C 60.96, H 5.44, N 15.03.

4-Chloro-1-(2-chloro-2-phenylethyl)-6-(trifluoromethyl)-1*H*-pyrazolo[3,4-*d*]pyrimidine **8d**. Yield: 84% (white solid). ^1^H NMR (200 MHz, DMSO-d_6_): δ 8.08 (s, 1H), 7.52–7.50 and 7.40–7.35 (2m, 5H), 5.77–5.65 (m, 1H), 5.23–4.98 (m, 2H). Mp: 85–87 °C. Elem. anal. calcd. for C_14_H_9_N_4_Cl_2_F_3_: C 46.56, H 2.51, N 15.51; found: 46.78, H 2.80, N 15.20.

4-Chloro-1-(2-chloro-2-phenylethyl)-6-phenyl-1*H*-pyrazolo[3,4-*d*]pyrimidine **8e**. Yield: 81% (white solid). Mp: 114–117 °C. ^1^H NMR (200 MHz, CDCl_3_): δ 8.59–8.56 (m, 2H), 8.16–8.14 (m, 1H), 7.59–7.51 (m, 5H), 7.48–7.31 (m, 3H), 5.63–5.59 (m, 1H), 5.21–4.98 (m, 2H). Elem. anal. calcd. for C_19_H_14_N_4_Cl_2_: C 61.80, H 3.82, N 15.17; found: C 61.74, H 4.03, N 14.83.

4-Chloro-1-[2-chloro-2-(4-chlorophenyl)ethyl]-6-methyl-1*H*-pyrazolo[3,4-*d*]pyrimidine **8f**. Yield: 94% (colorless oil). ^1^H NMR (200 MHz, CDCl_3_): δ 7.97 (s, 1H), 7.38–7.29 (m, 4H), 5.58–5.50 (m, 1H), 5.18–5.00 and 4.95–4.79 (2m, 2H), 2.70 (s, 3H). Elem. anal. calcd. for C_14_H_11_N_4_Cl_3_: C 49.22, H 3.25, N 16.40; found: C 49.13, H 2.98, N 16.31.

4-Chloro-1-[2-chloro-2-(4-chloro-phenyl)-ethyl]-6-ethyl-1*H*-pyrazolo[3,4-*d*]pyrimidine **8g**. Yield: 81% (yellow oil, that slowly crystallized). Mp: 63–65 °C. ^1^H NMR (200 MHz, DMSO-d_6_): δ 8.46 (s, 1H), 7.55–7.51 and 7.46–7.39 (2m, 4H), 5.78–5.66 (m, 1H), 5.17–4.92 (m, 2H), 2.96 (q, 2H, *J* = 7.6 Hz), 1.31 (t, 3H, *J* = 7.6 Hz). Elem. anal. calcd. for C_15_H_13_N_4_Cl_3_: C 50.66, H 3.68, N 15.75; found C 50.47, H 3.50, N 15.60.

4-Chloro-1-[2-chloro-2-(4-chlorophenyl)ethyl]-6-propyl-1*H*-pyrazolo[3,4-*d*]pyrimidine **8h**. Yield: 93% (yellow oil). ^1^H NMR (200 MHz, DMSO-d_6_): δ 7.90 (s, 1H), 7.58–7.39 (m, 4H), 5.44–5.38 (m, 1H), 5.09–5.01 (m, 2H), 2.65 (t, *J* = 7.7 Hz, 2H), 1.80 (sx, 2H, *J* = 7.7 Hz), 0.89 (t, 3H, *J* = 7.7 Hz). Elem. anal. calcd. for C_16_H_15_N_4_Cl_3_: C 51.98, H 4.09, N 15.16; found: C 52.20, H 4.17, N 15.21.

### 3.2. Enzymatic Assay

Src, Fyn, and Abl were purchased from Merck-Millipore. Reactions were performed according to the manufacturer’s instructions with minor modifications. All substrates were used at least at twice the concentration of apparent Km. In detail: Src reactions were performed using 500 μM Src-peptide (KVEKIGEGTYGVVYK), 100 μM ATP, and 0.00087% NP-40; Fyn reactions were performed using 360 μM Src-peptide (KVEKIGEGTYGVVYK), 200 μM ATP, and 0.0026% NP-40; Abl reactions were performed using 50 μM abltide (EAIYAAPFAKKK), 30 μM ATP, and 0.00087% NP-40. All reactions were performed using 10–50 ng of enzyme and 10% DMSO in 10 μL of kinase buffer (8 mM MOPS-NaOH pH7.0, 0.2 mM EDTA, 10 mM MgAc) at 30 °C for 10 min.

To avoid peptide adsorbing to the plastic surface, protein low-binding tubes were used. ADP-Glo kinase assay (Promega) was then used to detect kinase activity according to the manufacturer’s instructions with minor modifications. In detail, reactions were transferred to white 384 well-plates and stopped by adding 10 µL of ADP-Glo reagent (Promega) for 50 min at room temperature. A total of 20 μL of detection reagent (Promega) was then added for 30 min and the reaction read using a GloMax Discover microplate reader (Promega). Data were plotted using GraphPad Prism 5.0. IC_50_ values were obtained according to Equation (1):

v = V/{1 + (I/IC_50_)} (1)

where v is the measured reaction velocity, V is the apparent maximal velocity in the absence of inhibitor, I is the inhibitor concentration, and IC_50_ is the 50% inhibitory dose. Compounds tested were assumed to act as fully ATP-competitive inhibitors. Therefore, K_i_ values were calculated according to Equation (2):

K_i_ = IC_50_/(1+ K_m_/[S]) (2)

where K_i_ is the affinity of the inhibitor to the enzyme, S is the ATP concentration, and K_m_ is the affinity of ATP calculated according to the Michaelis–Menten equation.

### 3.3. Cell Biology

#### 3.3.1. Cell Culture

All glioblastoma cell lines were cultured as previously described [21]. In more detail, T98G (Elabscience) and U251 cells were cultured in DMEM supplemented with 10% fetal bovine serum and routinely tested negative for mycoplasma contamination. GBMSC83 cells, a well-characterized mesenchymal GBM cancer stem cellular model, were cultured in non-adherent conditions as 3D neurospheres in DMEM-F12 supplemented with B27 Supplement (50×), EGF (20 ng/mL), and hβFGF(10 ng/mL) as previously described in [22,23].

#### 3.3.2. Antibodies and Other Reagents

Anti-GAPDH (Santa Cruz 1:5000, D16H11); anti-Vinculin (Cell signaling 1:5000, 13901T); anti-Src (Cell signaling 1:1000, 2108S); anti-pY416-Src (Cell signaling 1:1000, 2101S).

Dasatinib (Sigma Aldrich, DAS, Merck Millipore, Burlington, MA, USA), SI388 and SI83 were used at different concentration (10 nM, 1 µM or 25 µM) for 24 h upon serum deprivation. DMSO (Sigma Aldrich) was used as the control.

#### 3.3.3. Protein Extracts and Immunoblotting Analysis

Cell extracts were prepared in RIPA (50 mM Tris-HCl pH 8.0, 150 mM NaCl, 1% NP40, 1 mM EGTA, 1 mM EDTA, 0.25% sodium deoxycholate) or IP buffer (50 mM Tris–HCl pH 7.5, 250 mM NaCl, 1% NP-40, 5 mM EDTA, 5 mM EGTA) supplemented with protease and phosphatase inhibitors (Roche Diagnostic, Mannheim, Germany). For immunoblotting, 25–30 µg of protein was separated by sodium dodecyl sulfate (SDS) polyacrylamide gel electrophoresis (PAGE), blotted onto nitrocellulose membrane, and detected with specific antibodies.

#### 3.3.4. Clonogenic Survival Assay

For colony formation, control and irradiated (5 Gy) cells were seeded at the concentration of 1000 cells/dish in 6-well culture plates and incubated at 37 °C, 5% CO_2_ for 10–15 days.

Crystal violet solution (10% (*v*/*v*) methanol and 0.5% Crystal Violet) was used to fix and stain colonies, upon 20 min incubation. The stained colonies (>50 cells) were counted under a microscope or using Image “Colonyarea” plugin. Data were expressed as the mean and SD of three independent experiments.

#### 3.3.5. MTS Assay

Cells were plated in 96 multiwells (1000 cells/well) in 100 µL of the complete medium. The following day, after 24 h of starvation, the cells were treated with DMSO, dasatinib or SI388 at different concentrations (10 nM, 1 µM, 25 µM). After 72 h, cells were treated with tetrazolium (Promega) for 1–2 h. The quantity of formazan product is directly proportional to the number of living cells in the culture. The plate is then read with a plate reader measuring the absorbance of 490 nm for the different conditions.

#### 3.3.6. Neurosphere Formation, Cell Counting and Sphere Size Measurement

GBMSC83 cells were seeded at the density of 1 × 10^4^ cells/well in a 96-well ultralow attachment plate, and the following day they were treated with SI388 1 µM or dasatinib 1 µM. After 72 h from treatment, cells were stained with Hoechst 33,342 (Thermo Fisher Scientific) and images were acquired with Fluorescence microscopy (ZEISS) and processed with Fiji version 2.3. Hoechst positive nuclei were counted by particle detection instrument of ComDet Plugin of Fiji. The bright fields were acquired and processed by Cell Profiler version 2.1.1 to measure spheres’ diameter and reported as the fold change from the control condition.

#### 3.3.7. Cell Death Analysis

1 × 10^6^ cells were analyzed for cell death 48 h from ionizing radiation (10 Gy) upon staining with Annexin V-APC-propidium iodide (PI) kit, according to manufacturer instructions (eBioscience™ Annexin V apoptosis detection kits, ThermoFisher Scientific, Waltham, MA, USA). Unstained samples were used as the control. CytoFLEX S (Beckman Coulter, Milan, Italy) instrument was used to quantify the double staining. Quality control was evaluated using CytoFLEX Daily QC Fluorospheres (Beckman Coulter). CytExpert version 2.2 software (Beckman Coulter) was used to analyze FCS files. Dead cells were graphed as fold change to control conditions.

#### 3.3.8. Statistical Analyses

All data were analyzed and presented as mean ± S.E.M. The significance of the differences between populations of data were assessed according to the paired or unpaired Student’s two-tailed *t*-test with a level of significance of at least *p* ≤ 0.05.

## 4. Conclusions

We performed a lead optimization study that afforded the discovery of the compound SI388 (**2a**), endowed with an improved activity compared to the parent compound SI83 (**1a**). This promising cell-free data prompted us to investigate the efficacy of SI388 in 2D and 3D tumor models characterized by Src overexpression. For this purpose, we decided to perform our biological experiments using GBM cellular models, which are known to be characterized by Src hyperactivation.

In this work, we tested SI388 compound on two commercial GBM cell lines (T98G and U251 cells) and on mesenchymal patient-derived cancer stem cells (GBMSC83 cells). Interestingly, SI388 can efficiently target Src and its functionality both in T98G and U251 cell lines as well as in GBMSC83 patient-derived neurospheres. Importantly, SI388 can sensitize patient derived GBM cells to IR treatment, similarly to dasatinib. Overall, this study identifies SI388 as a promising candidate for Src kinase inhibition and as a potential new tool to enhance responses to radiotherapy, which is worthy of further investigation.

## Figures and Tables

**Table 1:**
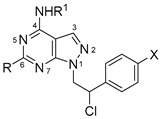
Structure of compounds **2a-s**.

	R	R^1^	X
**2a** (SI388)	SCH_3_	C_6_H_4_-2Cl	H
**2b**	SCH_3_	C_6_H_4_-4Cl	H
**2c**	SCH_2_CH_3_	C_6_H_5_	H
**2d**	SCH(CH_3_)_2_	C_6_H_4_-3Cl	H
**2e**	SCH_2_CH_2_-4-morpholin-1-yl	C_6_H_4_-2Cl	H
**2f**	SCH_2_CH_2_-4-morpholin-1-yl ∙ HCl	C_6_H_3_-3OH-4F	H
**2g**	SCH_2_CH_2_-4-morpholin-1-yl	C_6_H_2_-2F-4F-5OH	H
**2h**	CH_2_CH_3_	C_6_H_5_	H
**2i**	cyclopentyl	C_6_H_4_-3Cl	H
**2j**	cyclohexyl	C_6_H_5_	H
**2k**	CF_3_	C_6_H_5_	H
**2l**	CF_3_	C_6_H_4_-3F	H
**2m**	CF_3_	C_6_H_4_-3Cl	H
**2n**	C_6_H_5_	C_6_H_5_	H
**2o**	C_6_H_5_	C_6_H_4_-3Cl	H
**2p**	SCH_3_	C_6_H_5_	F
**2q**	CH_3_	C_6_H_5_	Cl
**2r**	CH_2_CH_3_	C_6_H_5_	Cl
**2s**	CH_2_CH_2_CH_3_	C_6_H_5_	Cl

**Table 2:** K_i_ towards Src, Fyn and Abl of compound **1a** (SI83) and derivatives **2a-s**.

	Src K_i_ (μM) ^a^	Fyn K_i_ (μM) ^a^	Bcr-Abl K_i_ (μM) ^a^
**1a** (SI83)	0.6	N.D. ^b^	0.6
**2a** (SI388)	0.423 ± 0.093	0.419 ± 0.075	0.45 ± 0.29
**2c**	N.D.	N.D.	N.D.
**2h**	N.D.	10.27 ± 3.13	N.D.
**2k**	N.D.	3.79 ± 1.01	N.D.
**2p**	14.44 ± 4.4	2.68 ± 0.37	N.D.
**2q**	16.49 ± 4	2.07 ± 1.1	N.D.
**2r**	N.D.	1.74 ± 0.71	N.D.
**2s**	N.D.	10.2 ± 2.11	N.D.

^a^ The values were obtained from the mean of at least three experiments. ^b^ N.D. = Not Determined.

## Data Availability

Data is contained within the article and Supplementary Material.

## Supplementary Materials

The following supporting information can be downloaded at: https://www.mdpi.com/article/10.3390/ph16070958/s1. Table S1: Percentage of Src inhibition at 100, 10 and 1 µM for compounds **2a-s**.
