# Supplementary material for: Identification and Biological Characterization of the Pyrazolo[3,4-d]pyrimidine Derivative SI388 Active as Src Inhibitor
Source: Pharmaceuticals (Basel). 2023 Jul 4;16(7):958. doi: 10.3390/ph16070958 (PMC10384936; doi:10.3390/ph16070958)
Supplement: Supplementary file 1 [file pharmaceuticals-16-00958-s001.zip › pharmaceuticals-2445548-supplementary.pdf]

Identification and Biological Characterization of the Pyrazolo[3,4-*d*]pyrimidine Derivative SI388 Active as Src Inhibitor

Claudia Contadini [1,2,†], Claudia Cirotti [1,2,†], Anna Carbone [3], Mehrdad Norouzi [1,2], Annarita Cianciusi [3], Emmanuele Crespan [4], Cecilia Perini [4], Giovanni Maga [4], Daniela Barilà [1,2,*], Francesca Musumeci [3,*] and Silvia Schenone [3]

1 Laboratory of Cell Signaling, IRCCS-Fondazione Santa Lucia, 00179 Rome, Italy; claudiacontadini@gmail.com (C.C.); claudiacirotti89@gmail.com (C.C.)
2 Department of Biology, University of Rome "Tor Vergata", 00133 Rome, Italy
3 Department of Pharmacy, University of Genoa, Viale Benedetto XV, 3, 16132 Genoa, Italy; annar.cianciusi@gmail.com (A.C.); schenone@difar.unige.it (S.S.)
4 Institute of Molecular Genetics (IGM), IGM-CNR, Via Abbiategrasso 207, 27100 Pavia, Italy; giovanni.maga@igm.cnr.it (G.M.)
* Correspondence: francesca.musumeci@unige.it (F.M.); daniela.barila@uniroma2.it (D.B.)
† These authors contributed equally to this work.

Table of contents

- **Table S1.** Percentage of Src inhibition at 100, 10 and 1 μM for compounds **2a-s**.
- Representative ^{1}H and ^{13}C NMR spectra of final compounds **2a-s** and intermediates **5b**, **6b**, **7a-h, 8a, 8c-g**.

Table S1. Percentage of Src inhibition at 100, 10 and 1 μM for compounds **2a-s**.

Cpd	% inhib Src			% inhib Fyn			% inhib Abl		
	100 μM	10 μM	1 μM	100 μM	10 μM	1 μM	100 μM	10 μM	1 μM
**2a**	N.D.	90	N.D.	N.D.	90.7	x	N.D.	72	x
**2b**	N.D.	N.A.	24	N.D.	N.A.	N.A.	N.D.	23	N.A.
**2c**	N.D.	27	16	N.D.	64	33	N.D.	35	18
**2d**	99.5	34	N.D.	26	8	N.D.	N.D.	24	NA
**2e**	N.D.	N.A.	N.A.	N.D.	N.D.	N.D.	N.D.	26,3	NA
**2f**	N.D.	79	N.A.	N.D.	N.D.	N.D.	N.D.	67	3
**2g**	N.D.	93.6	43.3	N.D.	N.D.	N.D.	N.D.	83	33.5
**2h**	98.3	12	N.D.	82	57	N.D.	N.D.	N.A.	34
**2i**	98.8	56	N.D.	19	15	N.D.	N.D.	N.A.	N.A.
**2j**	95.3	5	N.D.	38	13	N.D.	N.D.	N.A.	39
**2k**	99.2	50.3	N.D.	66	47	N.D.	N.D.	39	N.A.
**2l**	N.D.	N.A.	N.A.	N.D.	10	19	N.D.	N.A.	40
**2m**	N.D.	67	N.A.	N.D.	20	2.50	N.D.	18	15
**2n**	N.D.	13	11	N.D.	23	16	N.D.	18	5
**2o**	N.D.	N.A.	N.A.	N.D.	8	N.A.	N.D.	N.A.	N.A.
**2p**	N.D.	55	14	N.D.	60	15	N.D.	12	N.A.
**2q**	N.D.	48	29	N.D.	78	24	N.D.	25	20
**2r**	N.D.	7.5	N.A.	N.D.	75	48	N.D.	N.A.	N.A.

**2s**	N.D.	19	24	N.D.	54	N.A.	N.D.	23	N.A.

N.D. = Not determined

N.A. = Not Active

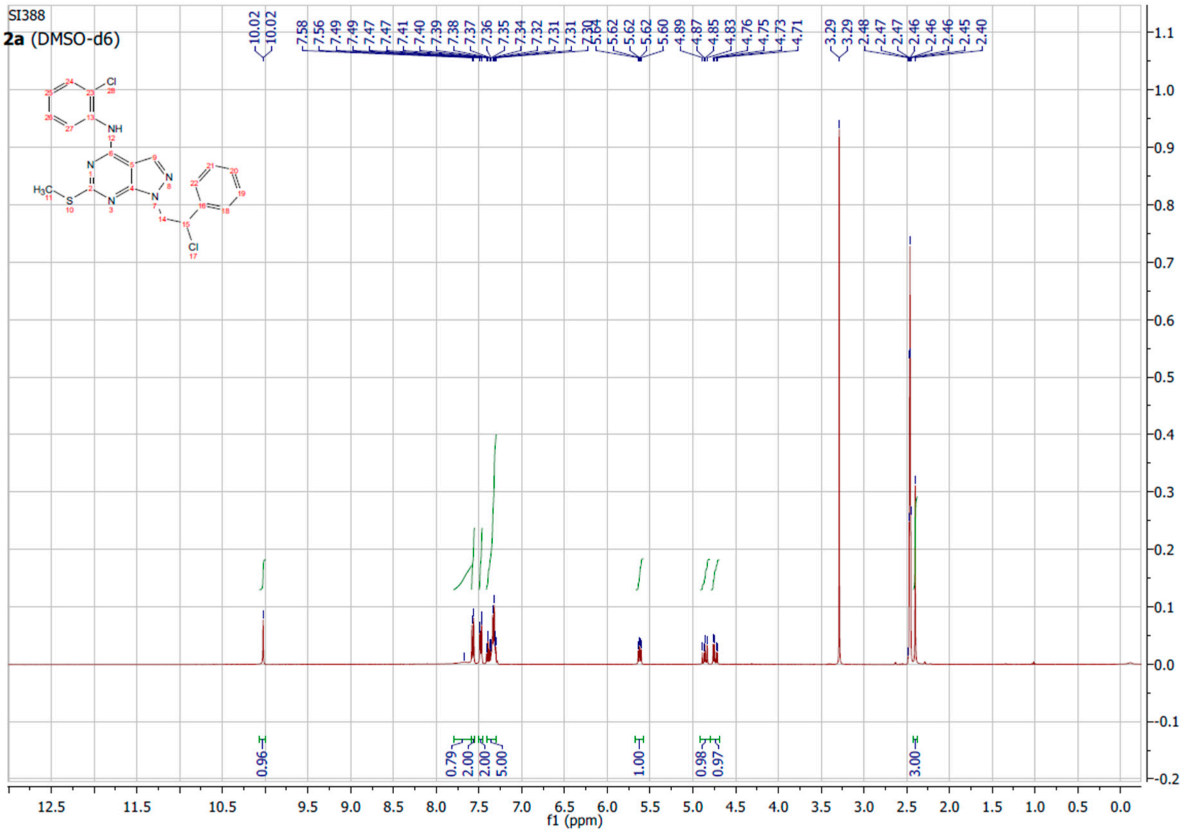

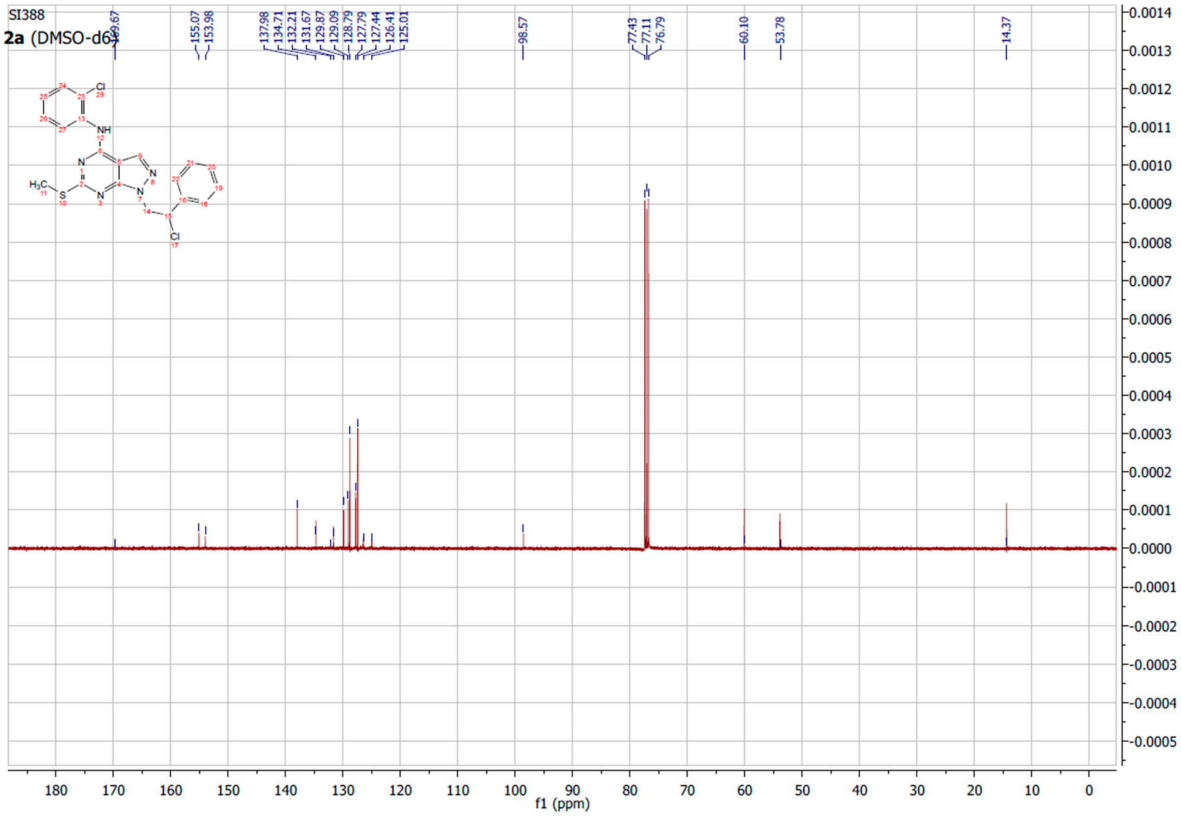

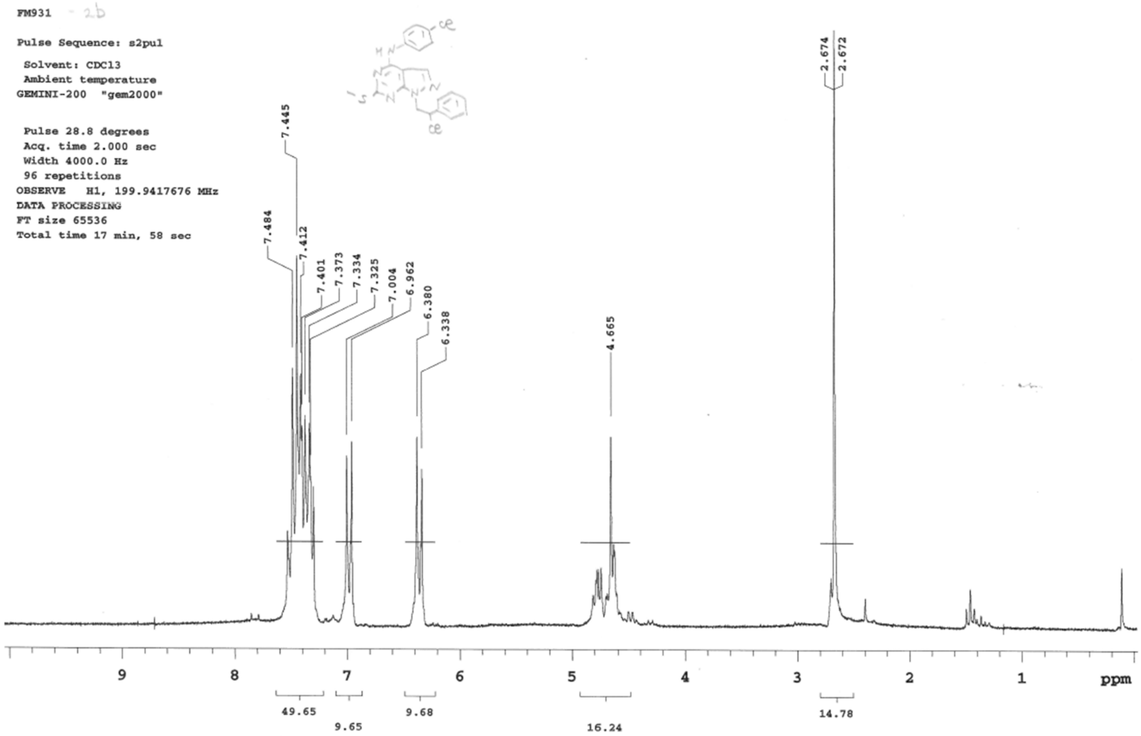
FM931
Pulse Sequence: s2pul
Solvent: CDCl3
Ambient temperature
GEMINI-200 "gem2000"
Pulse 28.8 degrees
Acq. time 2.000 sec
Width 4000.0 Hz
96 repetitions
OBSERVE H1, 199.9417676 MHz
DATA PROCESSING
FT size 65536
Total time 17 min, 58 sec
7.484
7.445
7.412
7.401
7.373
7.334
7.325
7.004
6.962
6.380
6.338
4.665
2.674
2.672
9 8 7 6 5 4 3 2 1 ppm
49.65
9.65
9.68
16.24
14.78

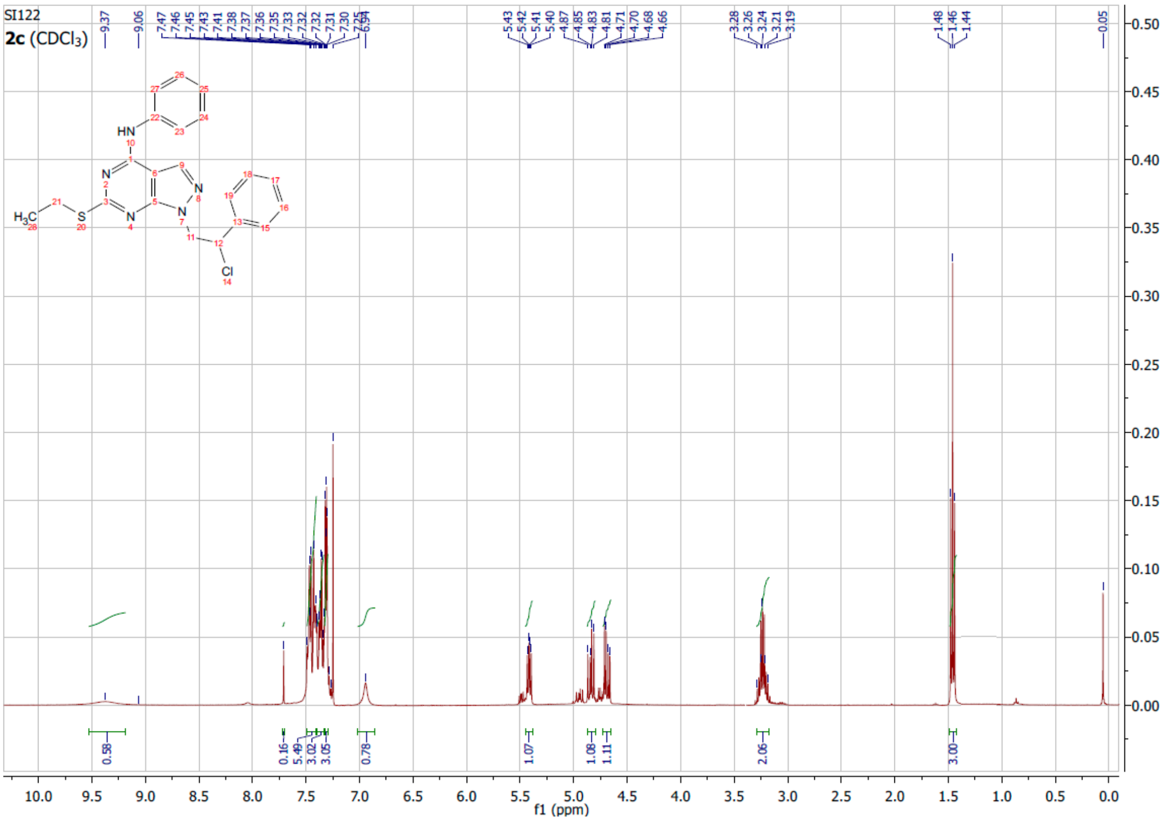
SI122
2c (CDCl3)
9.37
9.06
7.47 7.46 7.45 7.43 7.41 7.38 7.37 7.36 7.35 7.33 7.32 7.32 7.31 7.30 6.95 6.94
5.43 5.42 5.41 5.40 4.87 4.85 4.83 4.81 4.71 4.70 4.68 4.66
3.28 3.26 3.24 3.21 3.19
1.48 1.46 1.44
0.05
HN
N
N
N
N
S
H3C
Cl
0.58
0.16
5.49
3.02
3.05
0.78
1.07
1.08
1.11
2.06
3.00
10.0 9.5 9.0 8.5 8.0 7.5 7.0 6.5 6.0 5.5 5.0 4.5 4.0 3.5 3.0 2.5 2.0 1.5 1.0 0.5 0.0
f1 (ppm)
0.50 0.45 0.40 0.35 0.30 0.25 0.20 0.15 0.10 0.05 0.00

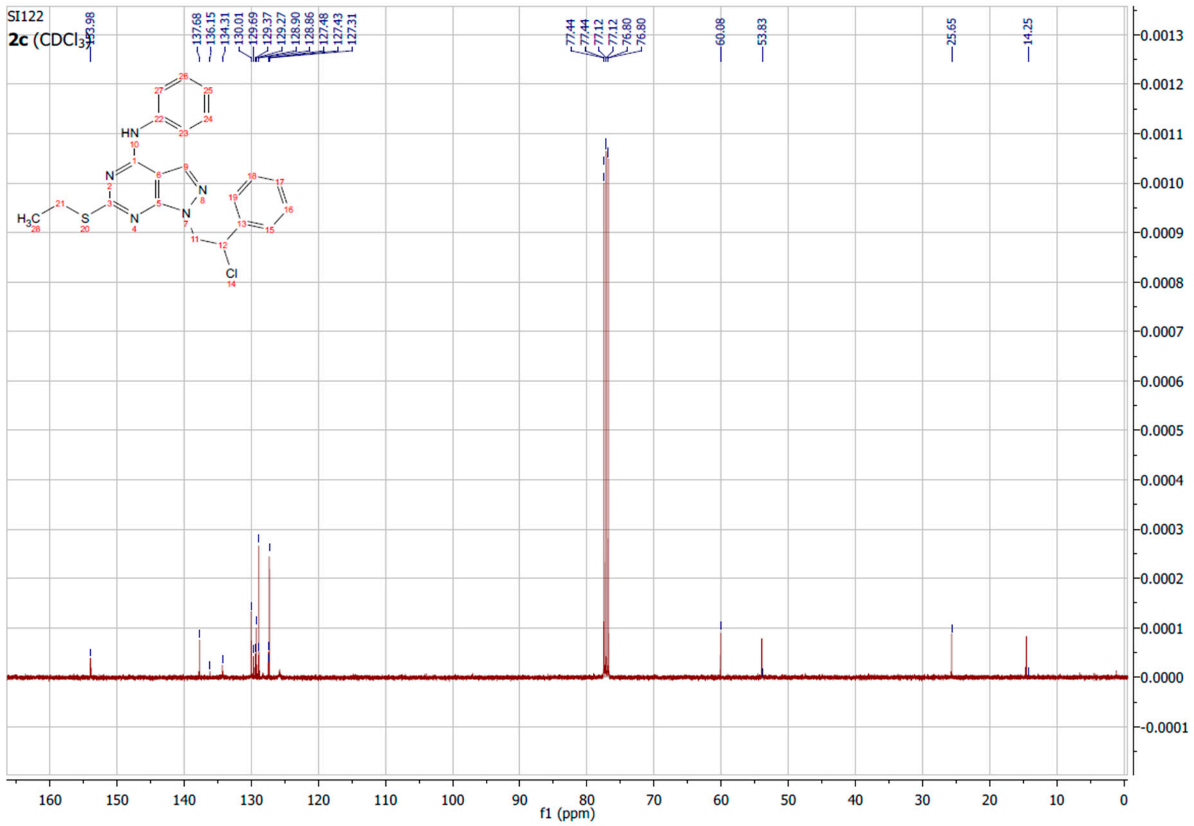
SI122
2c (CDCl3)
153.98
137.68
136.15
134.31
130.01
129.69
129.37
129.27
128.90
128.86
127.48
127.43
127.31
77.44
77.44
77.12
77.12
76.80
76.80
60.08
53.83
25.65
14.25
HN
N
S
N
N
N
Cl
H3C
f1 (ppm)

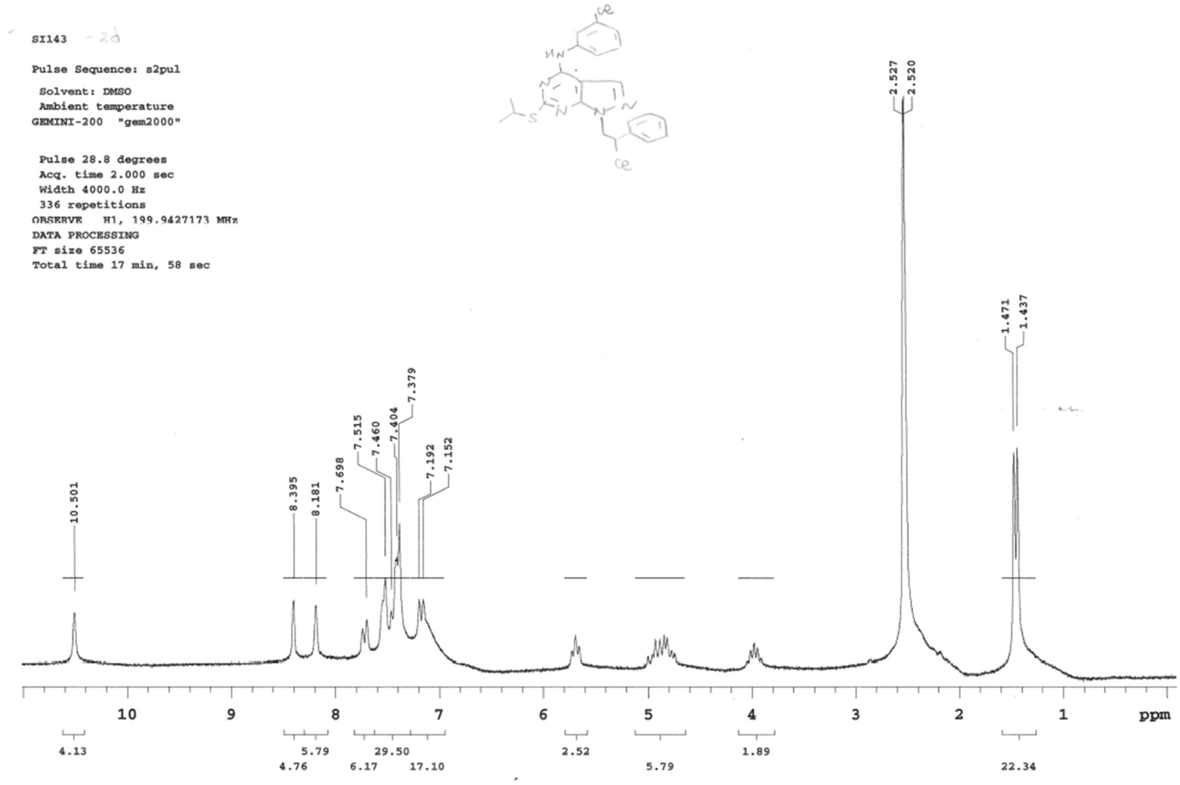
SI143
Pulse Sequence: s2pul
Solvent: DMSO
Ambient temperature
GEMINI-200 "gem2000"
Pulse 28.8 degrees
Acq. time 2.000 sec
Width 4000.0 Hz
336 repetitions
OBSERVE H1, 199.9427173 MHz
DATA PROCESSING
FT size 65536
Total time 17 min, 58 sec
10.501
8.395
8.181
7.698
7.515
7.460
7.404
7.379
7.192
7.152
2.527
2.520
1.471
1.437
ppm
4.13
5.79
4.76
29.50
6.17
17.10
2.52
5.79
1.89
22.34

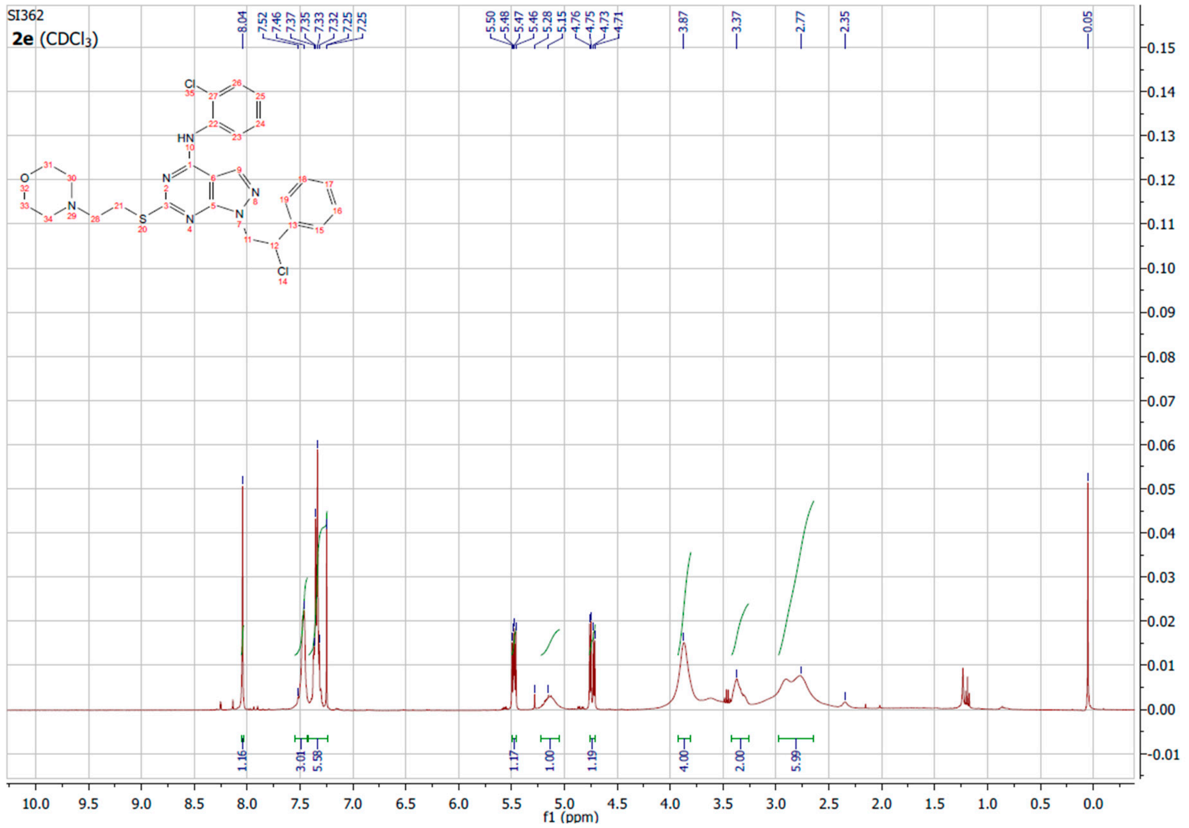
SI362
2e (CDCl3)
8.04
7.52
7.46
7.37
7.35
7.33
7.32
7.25
7.25
5.50
5.48
5.47
5.46
5.28
5.15
4.76
4.75
4.73
4.71
3.87
3.37
2.77
2.35
0.05
1.16
3.01
5.58
1.17
1.00
1.19
4.00
2.00
5.99
f1 (ppm)

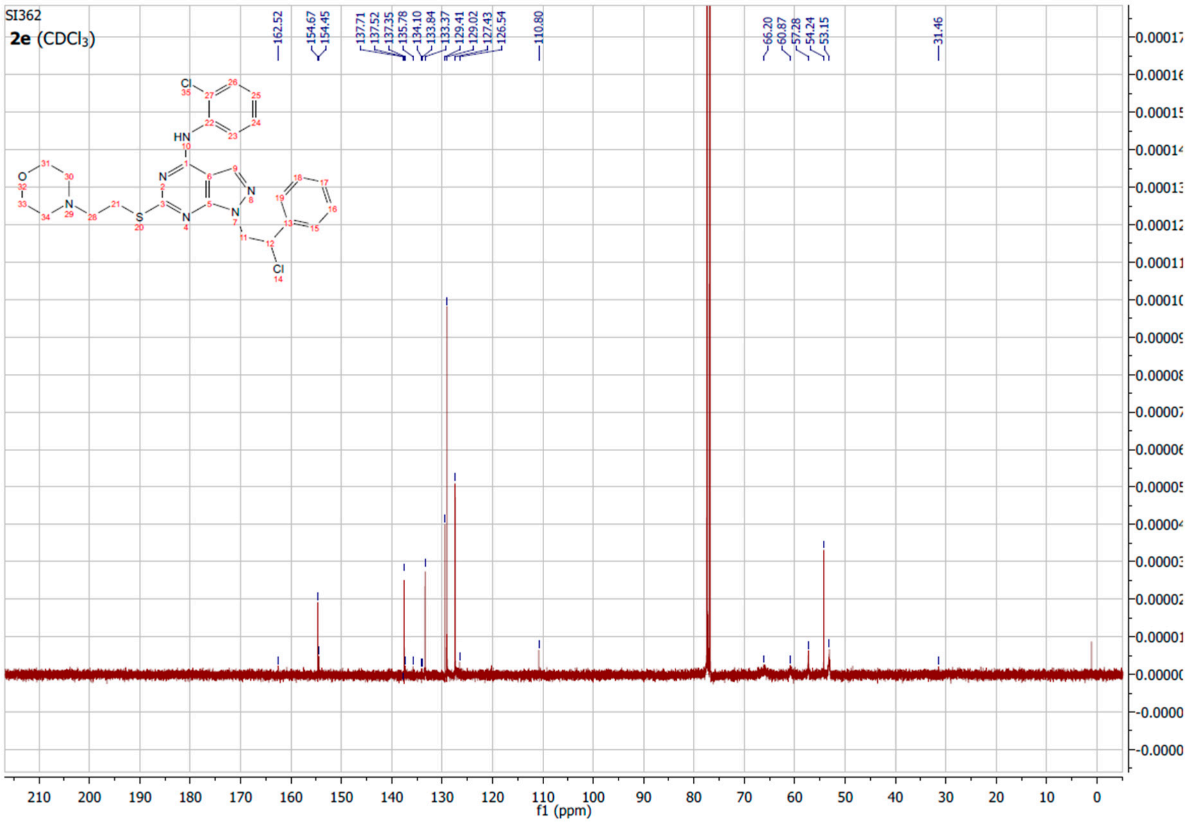
SI362
2e (CDCl3)
162.52
154.67
154.45
137.71
137.52
137.35
135.78
134.10
133.84
133.37
129.41
129.02
127.43
126.54
110.80
66.20
60.87
57.28
54.24
53.15
31.46
f1 (ppm)

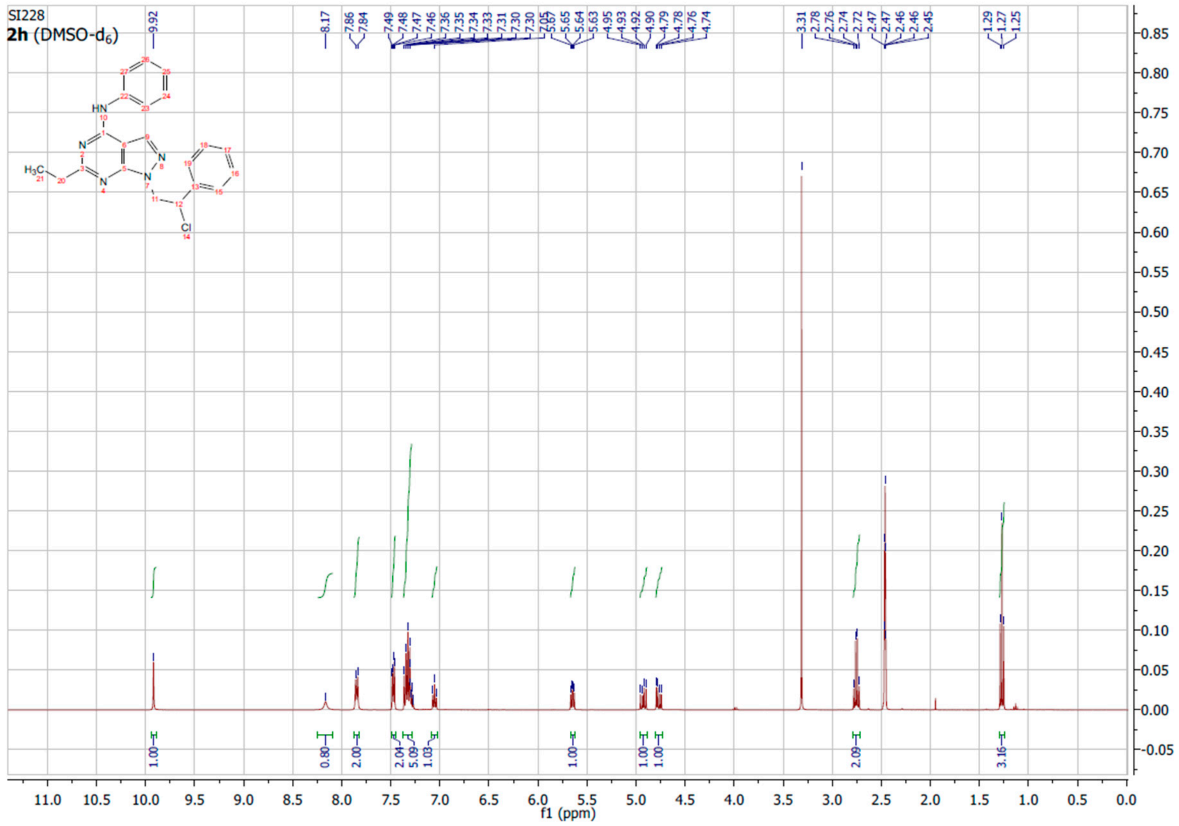
SI228
2h (DMSO-d6)
9.92
8.17
7.86
7.84
7.49
7.48
7.47
7.46
7.36
7.35
7.34
7.33
7.31
7.30
7.30
7.05
5.67
5.65
5.64
5.63
4.95
4.93
4.92
4.90
4.79
4.78
4.76
4.74
3.31
2.78
2.76
2.74
2.72
2.47
2.46
2.46
2.45
1.29
1.27
1.25
1.00
0.80
2.00
2.04
5.09
1.03
1.00
1.00
1.00
2.09
3.16
f1 (ppm)

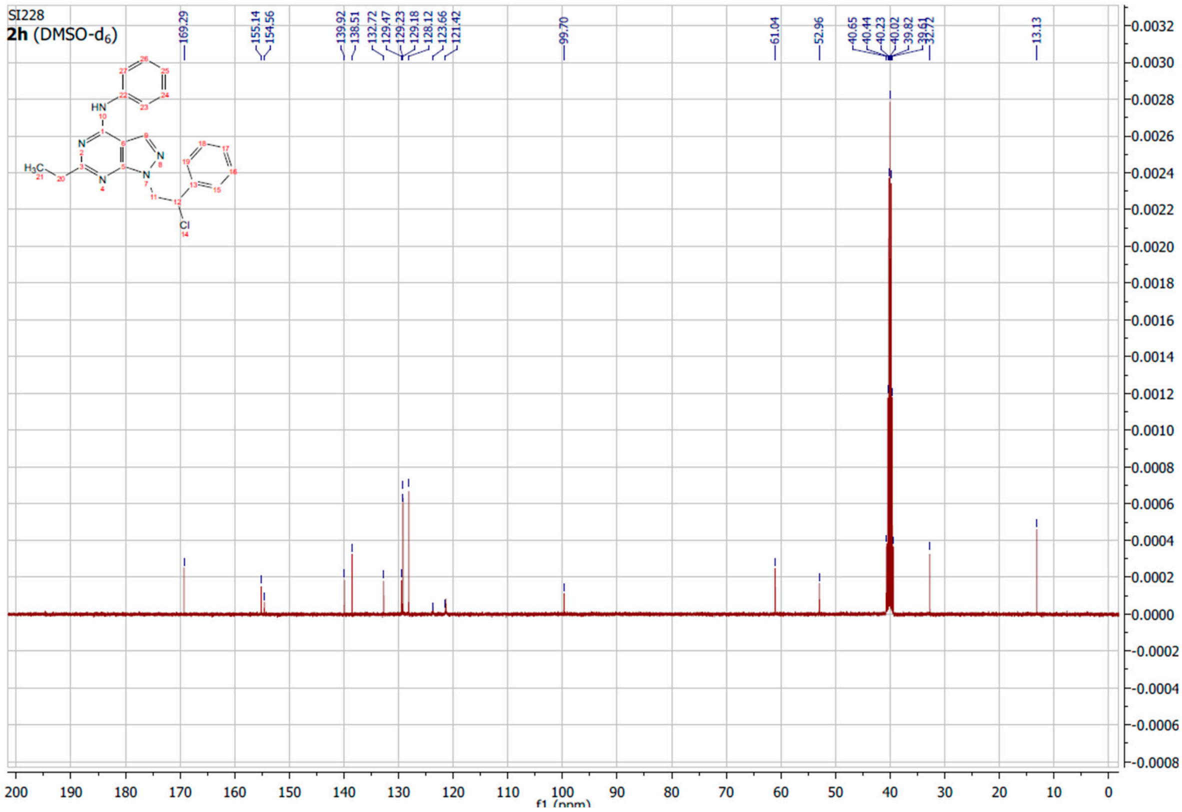
SI228
2h (DMSO-d6)
169.29
155.14
154.56
139.92
138.51
132.72
129.47
129.23
129.18
128.12
123.66
121.42
99.70
61.04
52.96
40.65
40.44
40.23
40.02
39.82
39.61
32.92
13.13

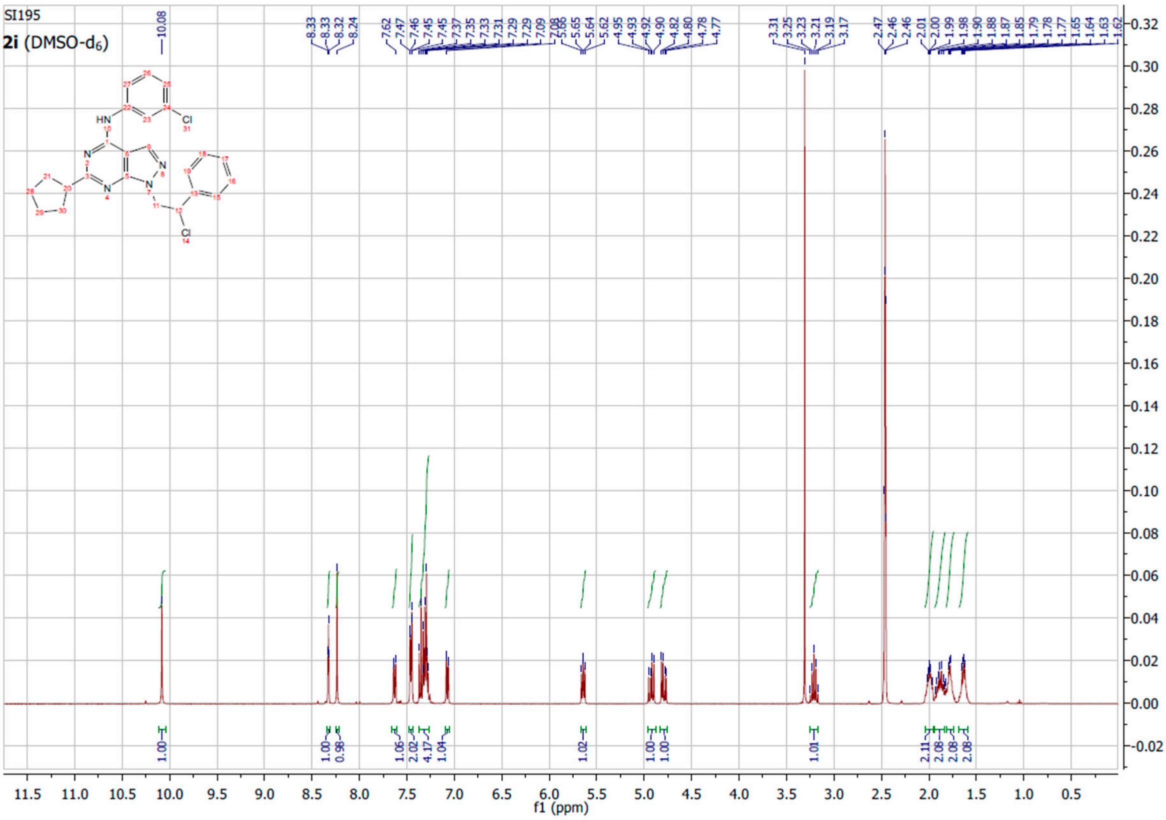
SI195
2i (DMSO-d6)
10.08
8.33 8.33 8.32 8.24
7.62 7.47 7.46 7.45 7.45 7.37 7.35 7.33 7.33 7.31 7.29 7.29 7.09 7.08
5.66 5.65 5.64 5.62
4.95 4.93 4.92 4.90 4.82 4.80 4.78 4.77
3.31 3.25 3.23 3.21 3.19 3.17
2.47 2.46 2.46
2.01 2.00 1.99 1.98 1.90 1.88 1.87 1.85 1.79 1.78 1.77 1.65 1.64 1.63 1.62
1.00 1.00 0.98 1.06 2.02 4.17 1.04 1.02 1.00 1.00 1.01 2.11 2.08 2.08 2.08
f1 (ppm)

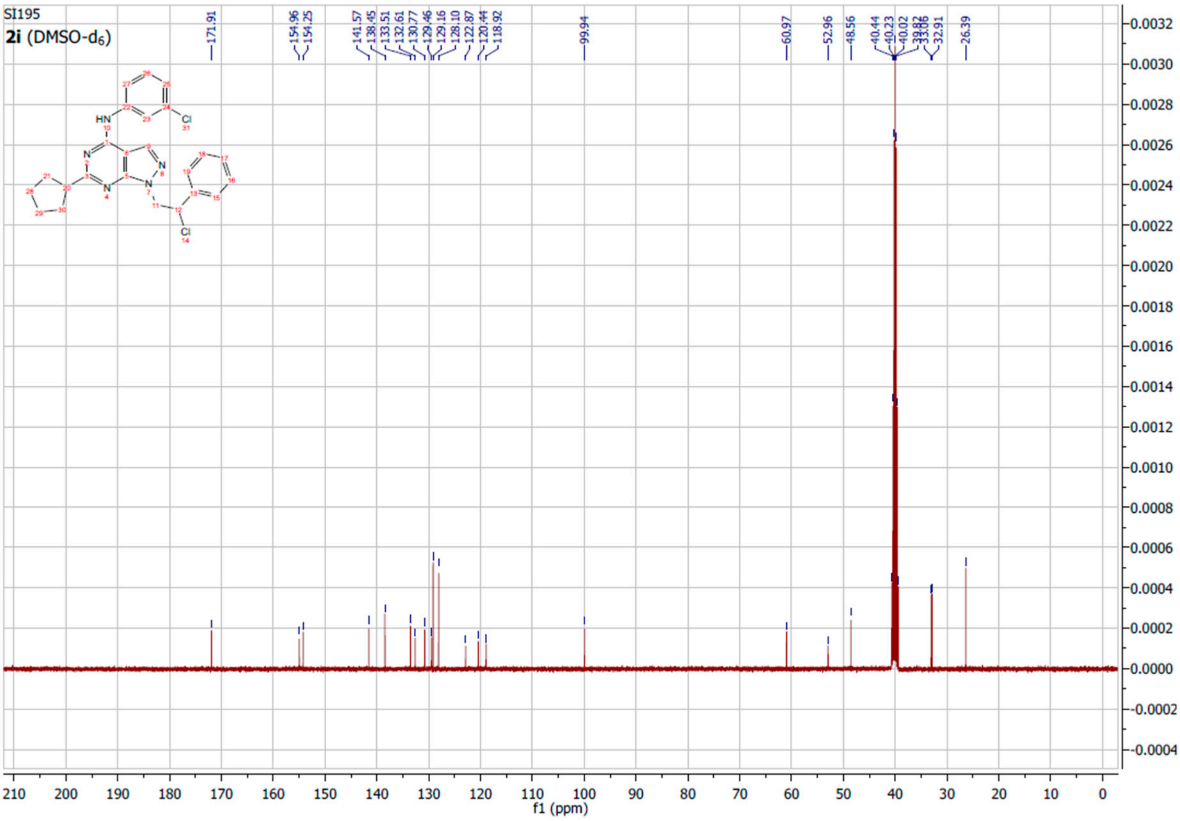
SI195
2i (DMSO-d6)
171.91
154.96 154.25
141.57 138.45 133.51 132.61 130.77 129.46 129.16 128.10 122.87 120.44 118.92
99.94
60.97
52.96
48.56
40.44 40.23 40.02
32.91
26.39
f1 (ppm)

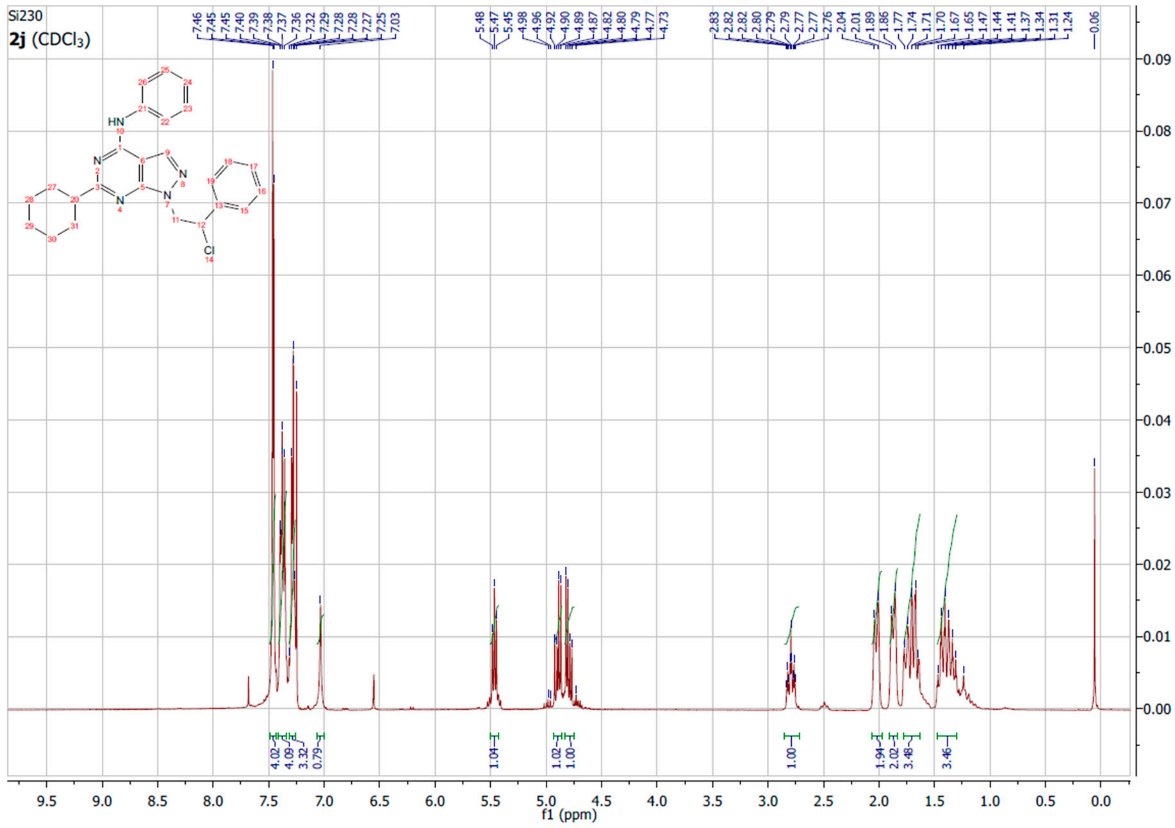
Si230
2j (CDCl3)
7.46 7.45 7.45 7.40 7.39 7.38 7.37 7.36 7.32 7.29 7.28 7.28 7.27 7.25 7.03
5.48 5.47 5.45 4.98 4.96 4.92 4.90 4.89 4.87 4.82 4.80 4.79 4.77 4.73
2.83 2.82 2.82 2.80 2.79 2.79 2.77 2.77 2.76 2.04 2.01 1.89 1.86 1.77 1.74 1.71 1.70 1.67 1.65 1.47 1.44 1.41 1.37 1.34 1.31 1.24
0.06
4.02 4.09 3.32 0.79 1.04 1.02 1.00 1.00 1.94 2.02 3.48 3.46
f1 (ppm)

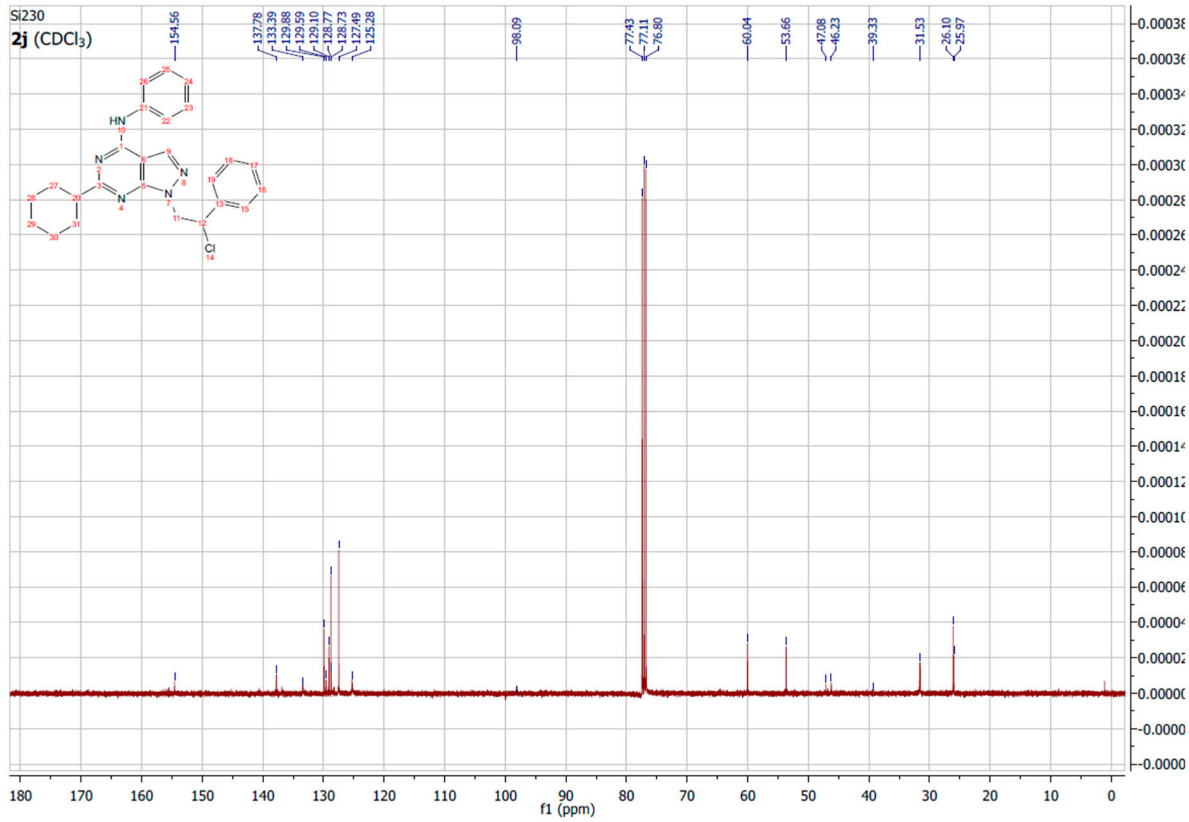
Si230
2j (CDCl3)
154.56
137.78 133.39 129.88 129.59 129.10 128.77 128.73 127.49 125.28
98.09
77.43 77.11 76.80
60.04
53.66
47.08 46.23
39.33
31.53
26.10 25.97
f1 (ppm)

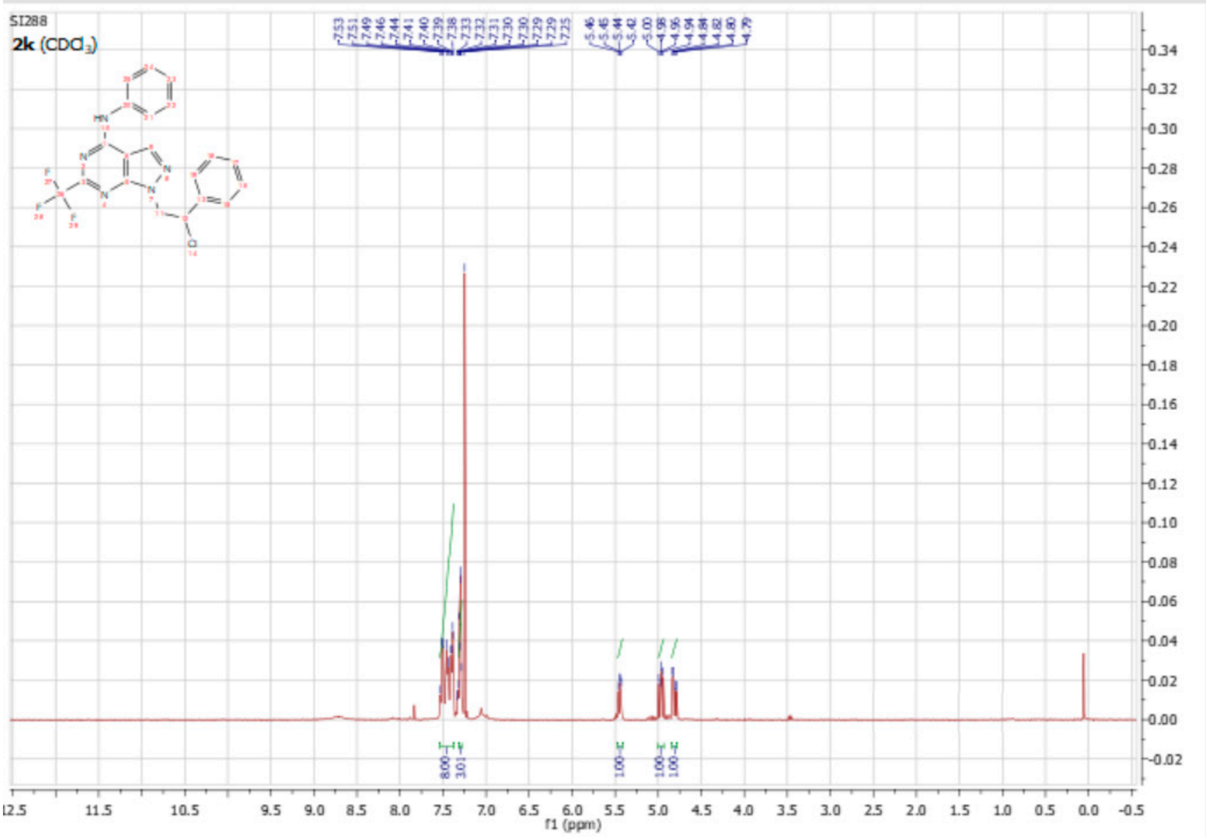
SI288
2k (CDCl3)
7.53 7.51 7.49 7.46 7.44 7.41 7.40 7.39 7.38 7.33 7.32 7.31 7.30 7.30 7.29 7.29 7.25
5.46 5.45 5.44 5.42 5.00 4.98 4.96 4.94 4.84 4.82 4.80 4.79
8.00 3.01 1.00 1.00 1.00
f1 (ppm)

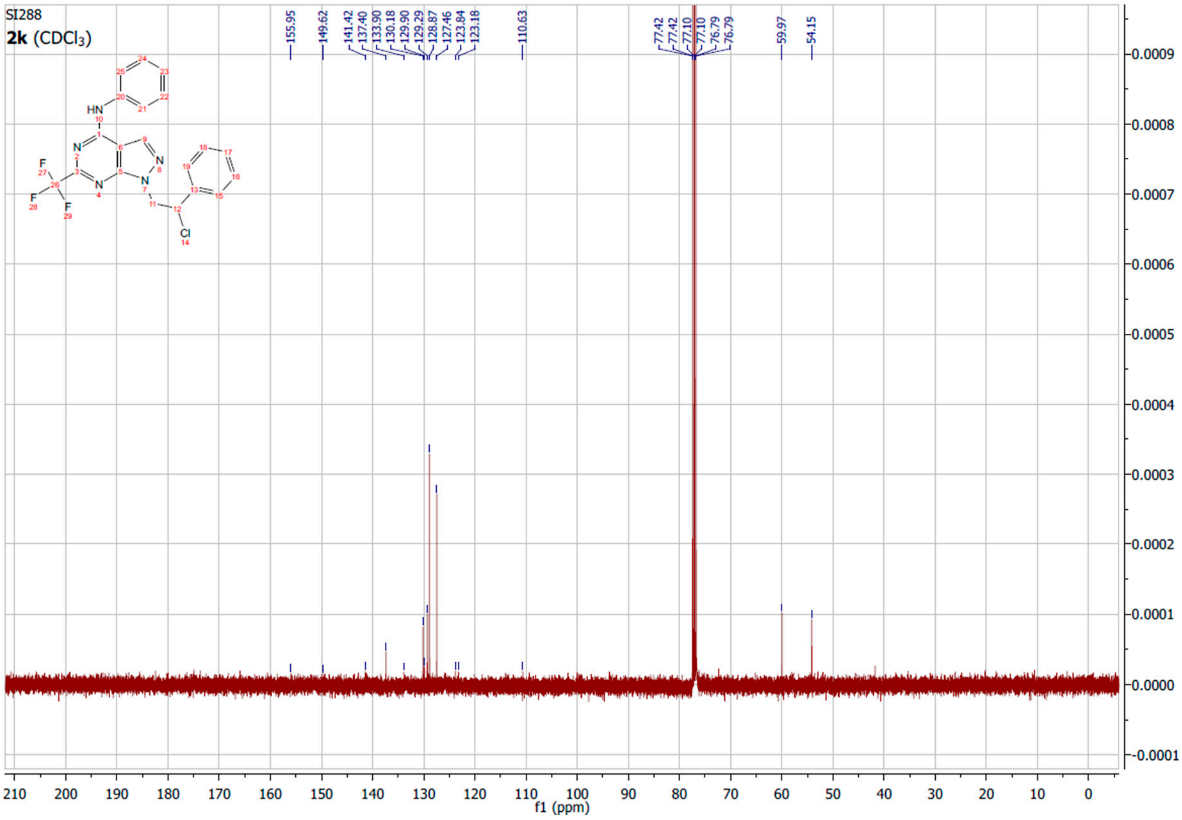
SI288
2k (CDCl3)
155.95 149.62 141.42 137.40 133.90 130.18 129.90 129.29 128.87 127.46 123.84 123.18 110.63
77.42 77.42 77.10 77.10 76.79 76.79 59.97 54.15
f1 (ppm)

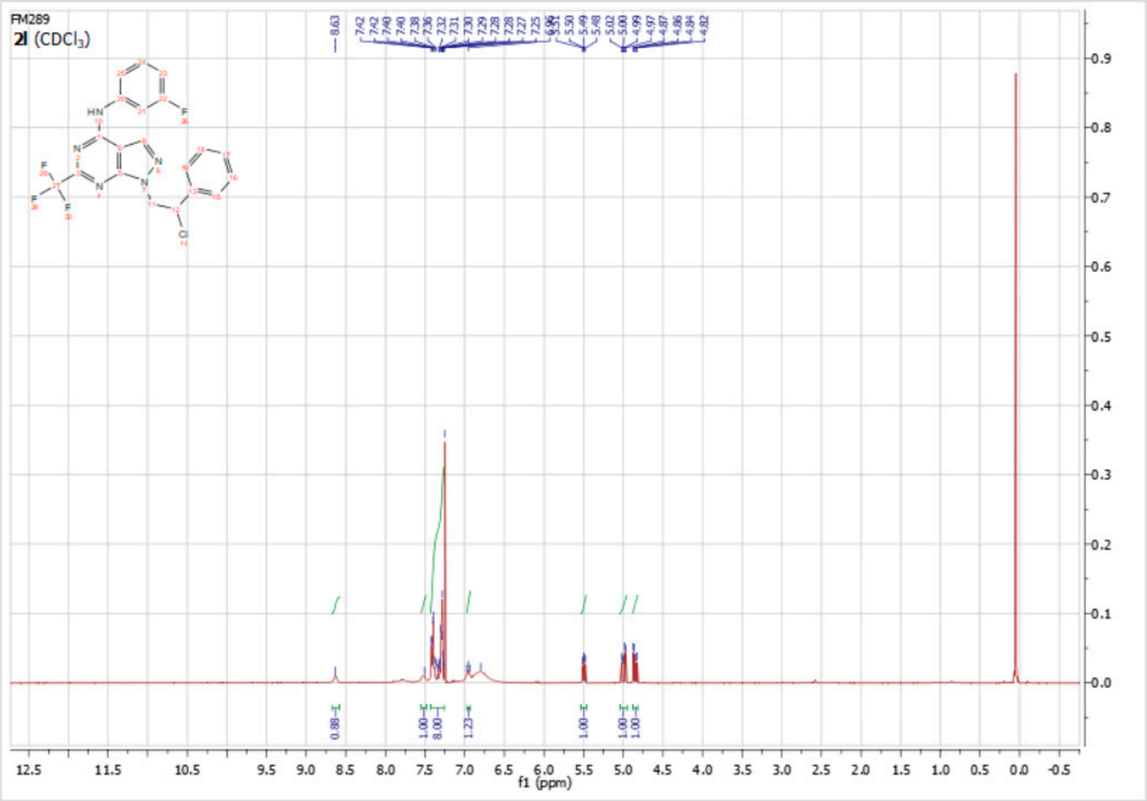
FM289
2l (CDCl3)
f1 (ppm)

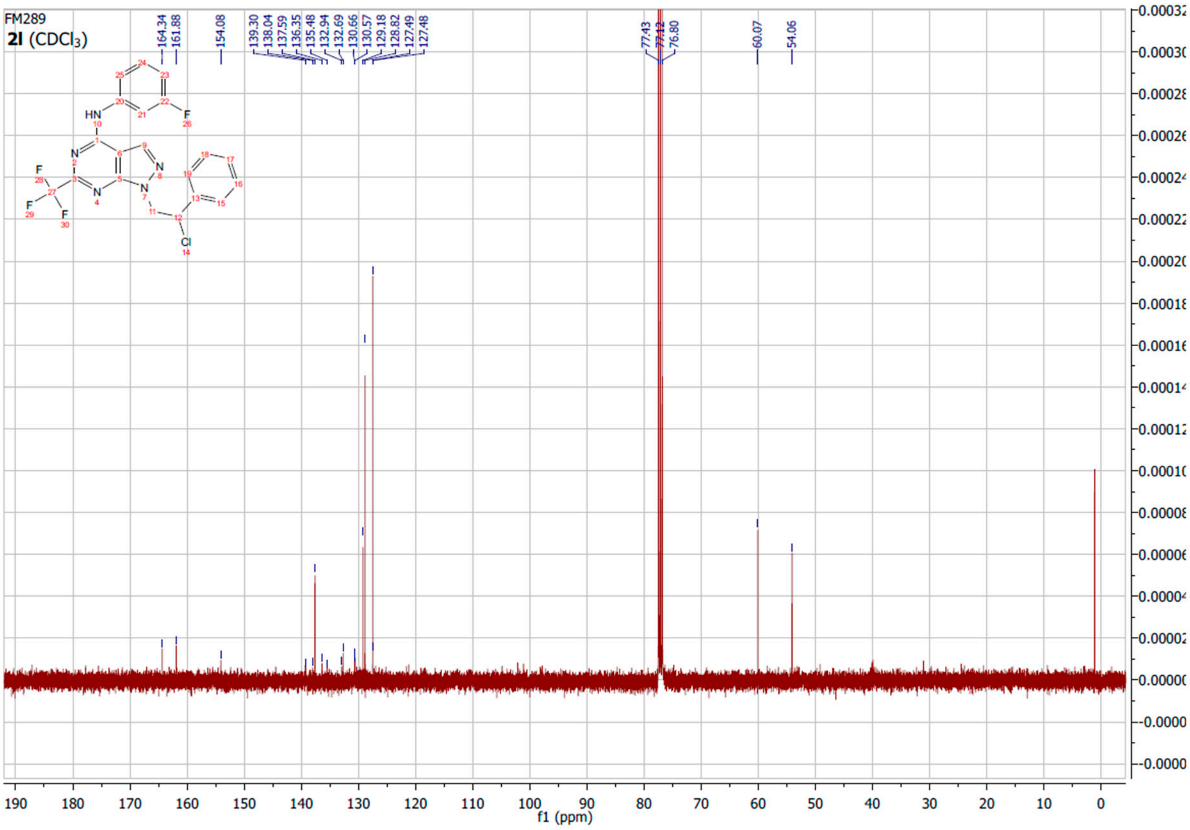
FM289
2l (CDCl3)
164.34
161.88
154.08
77.43
77.12
76.80
60.07
54.06
f1 (ppm)

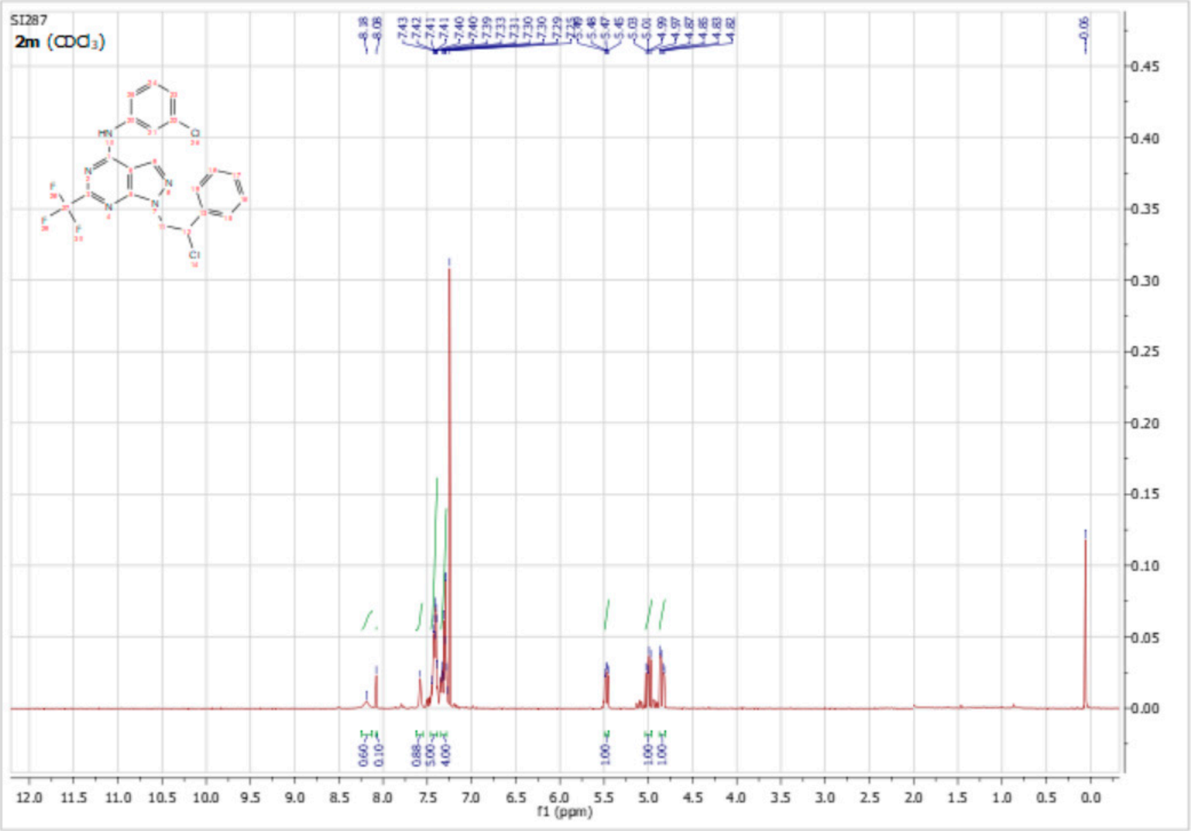
SI287
2m (CDCl3)
8.18
8.08
7.43
7.42
7.41
7.41
7.40
7.40
7.39
7.33
7.31
7.30
7.30
7.29
5.48
5.47
5.46
5.03
5.01
4.99
4.97
4.87
4.85
4.83
4.82
0.06
0.60
0.10
0.88
5.00
4.00
1.00
1.00
1.00
f1 (ppm)

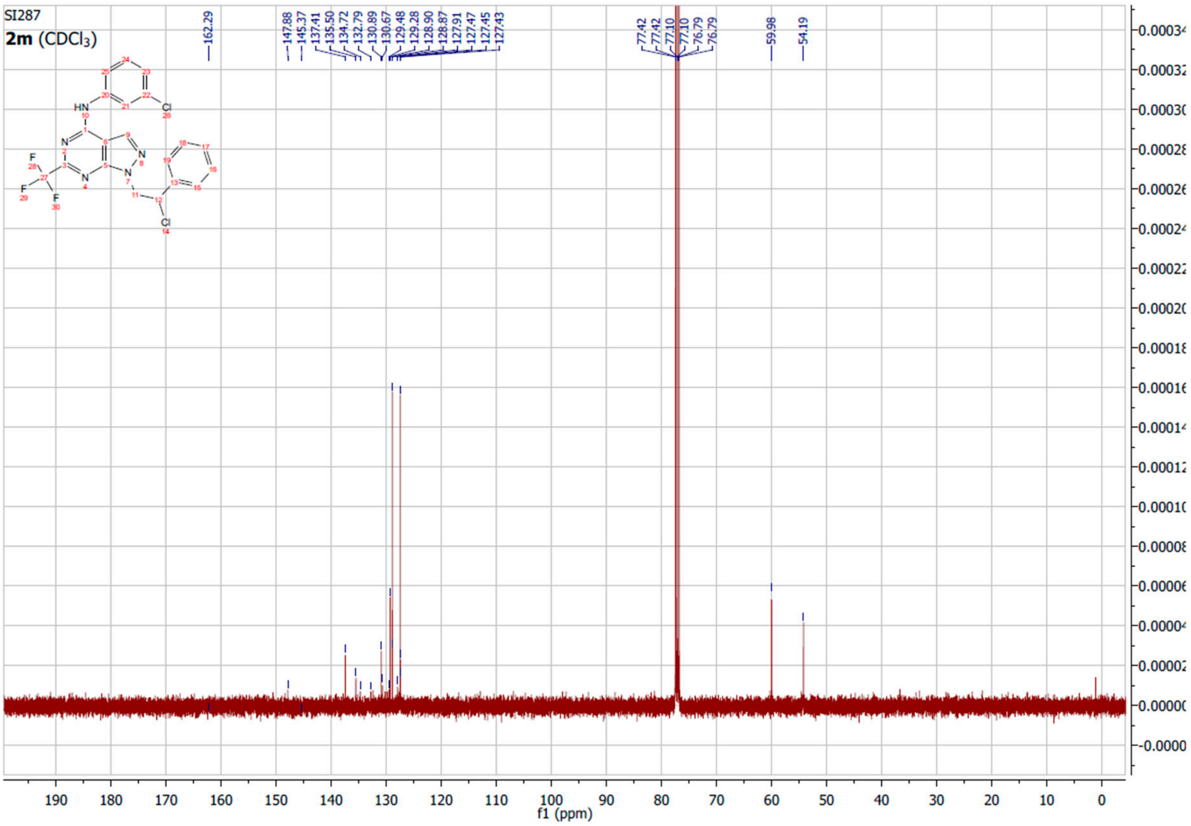
SI287
2m (CDCl3)
162.29
147.88
145.37
137.41
135.50
134.72
132.79
130.89
130.67
129.48
129.28
128.90
128.87
127.91
127.47
127.45
127.43
77.42
77.42
77.10
76.79
76.79
59.98
54.19
f1 (ppm)

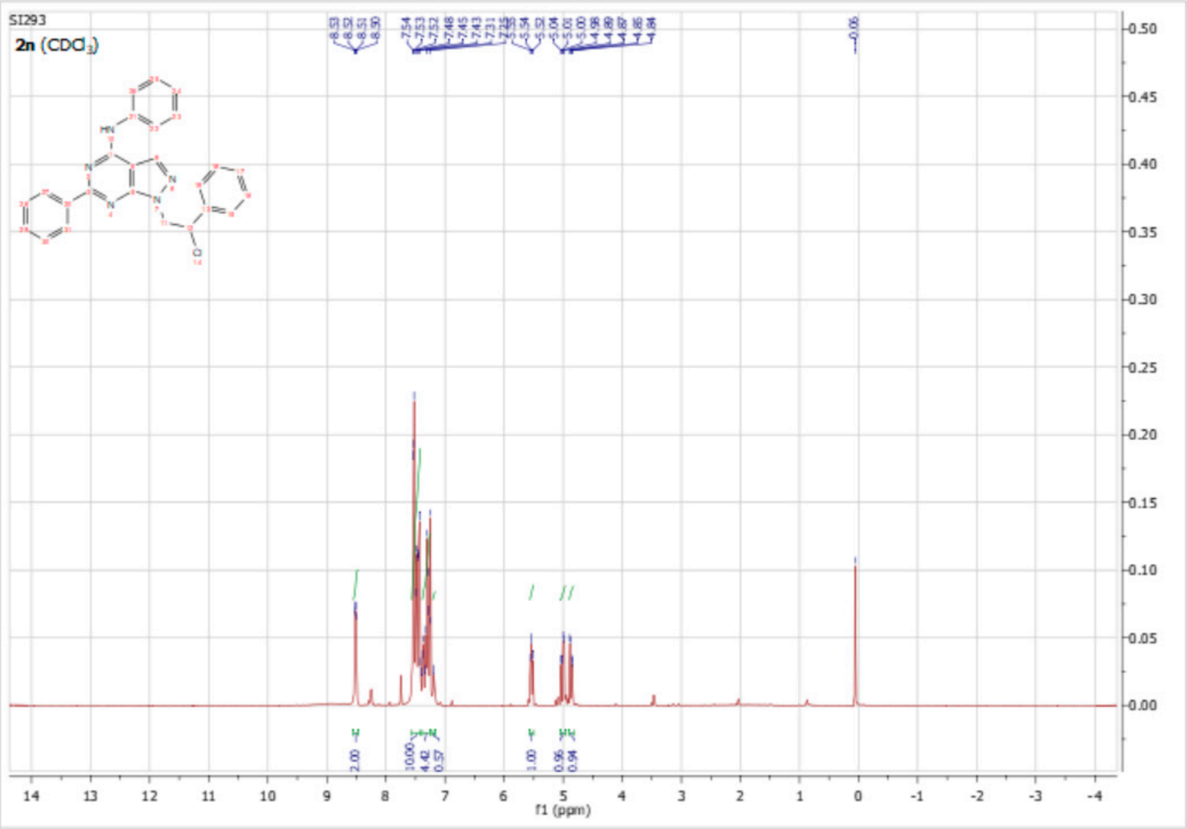
SI293
2n (CDCl3)
f1 (ppm)

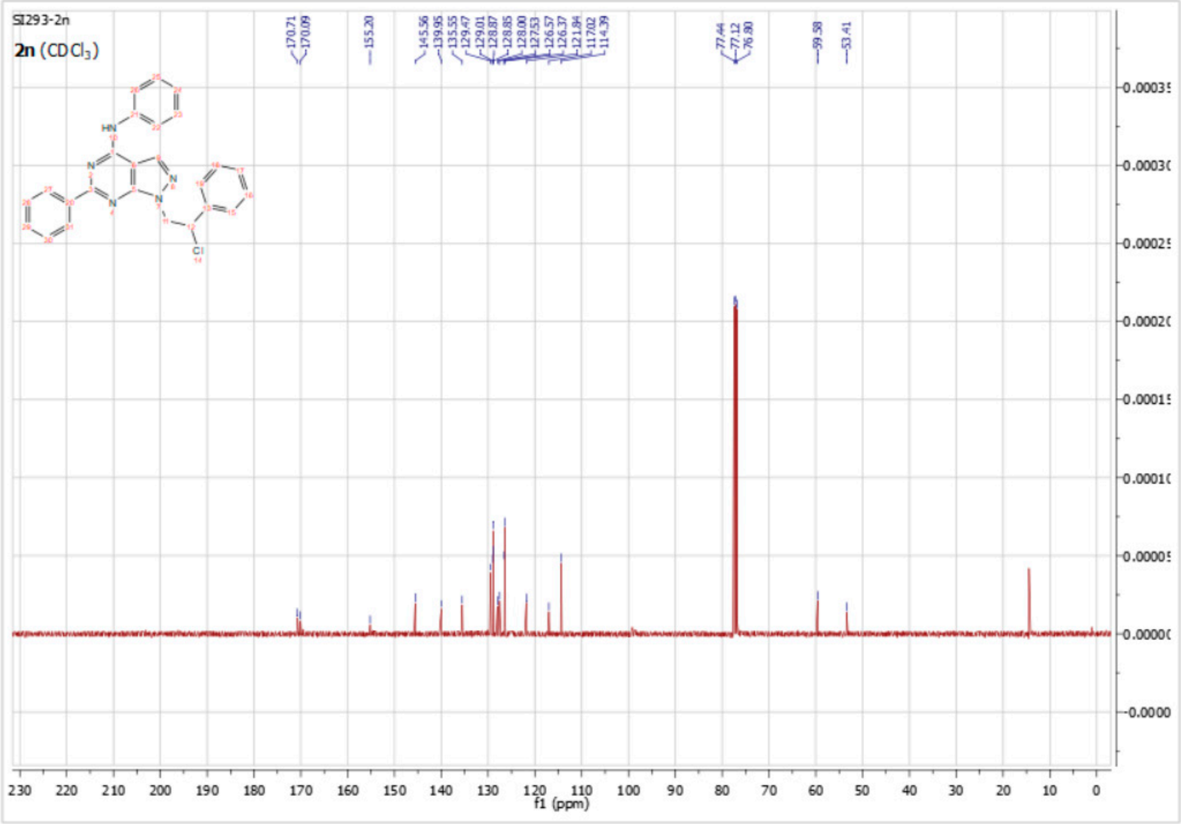
SI293-2n
2n (CDCl3)
f1 (ppm)

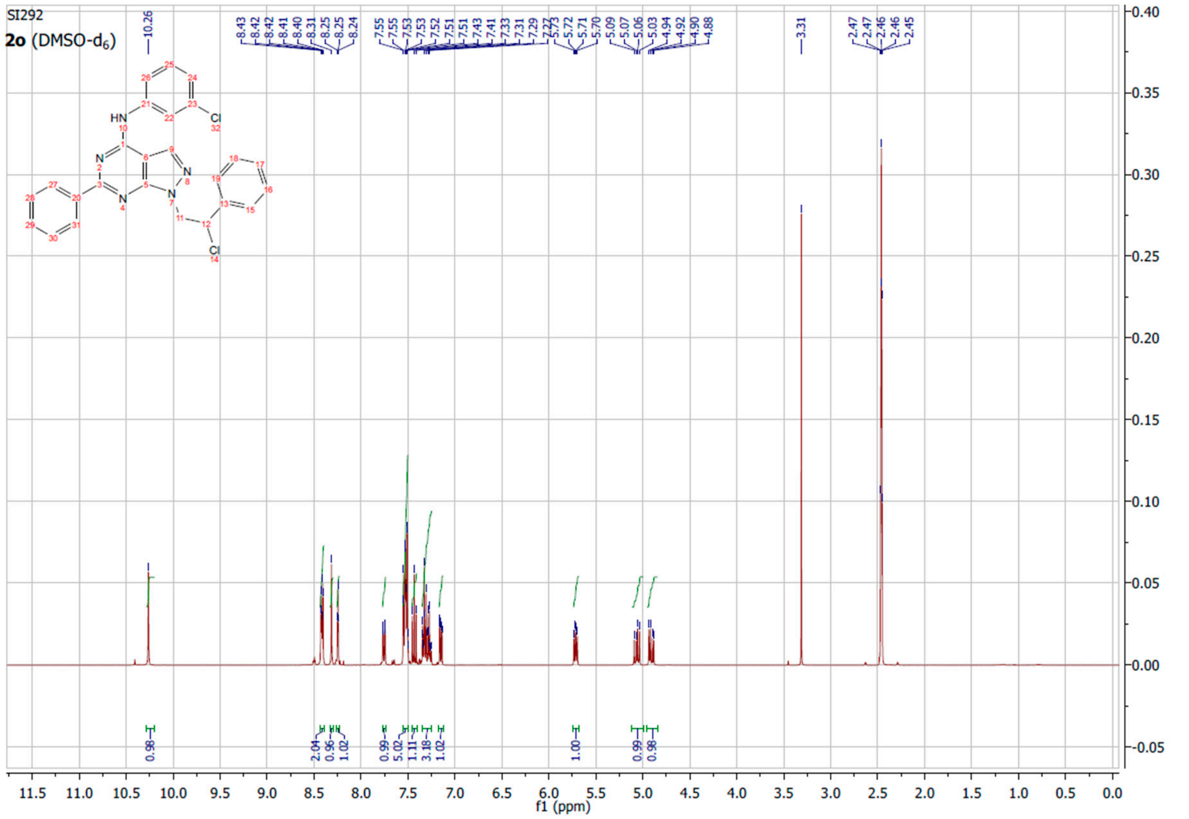
SI292
2o (DMSO-d6)
f1 (ppm)

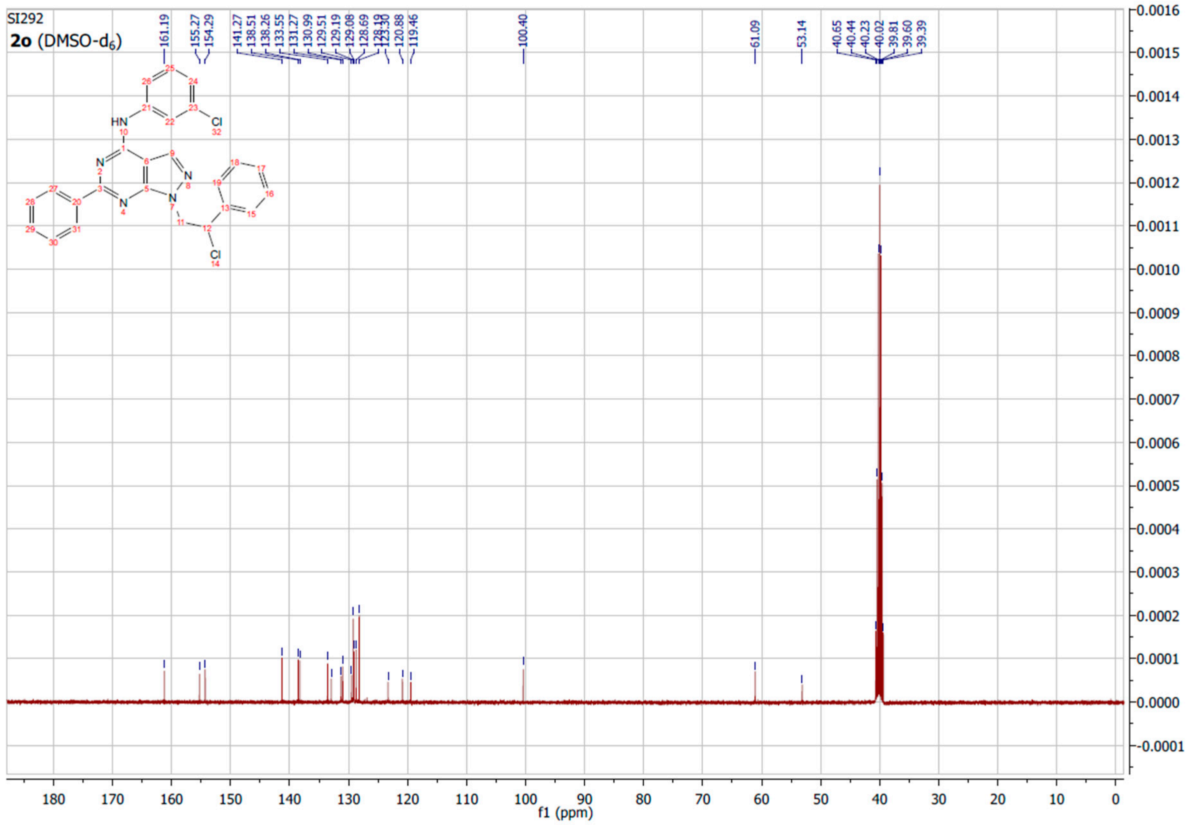
SI292
2o (DMSO-d6)
f1 (ppm)

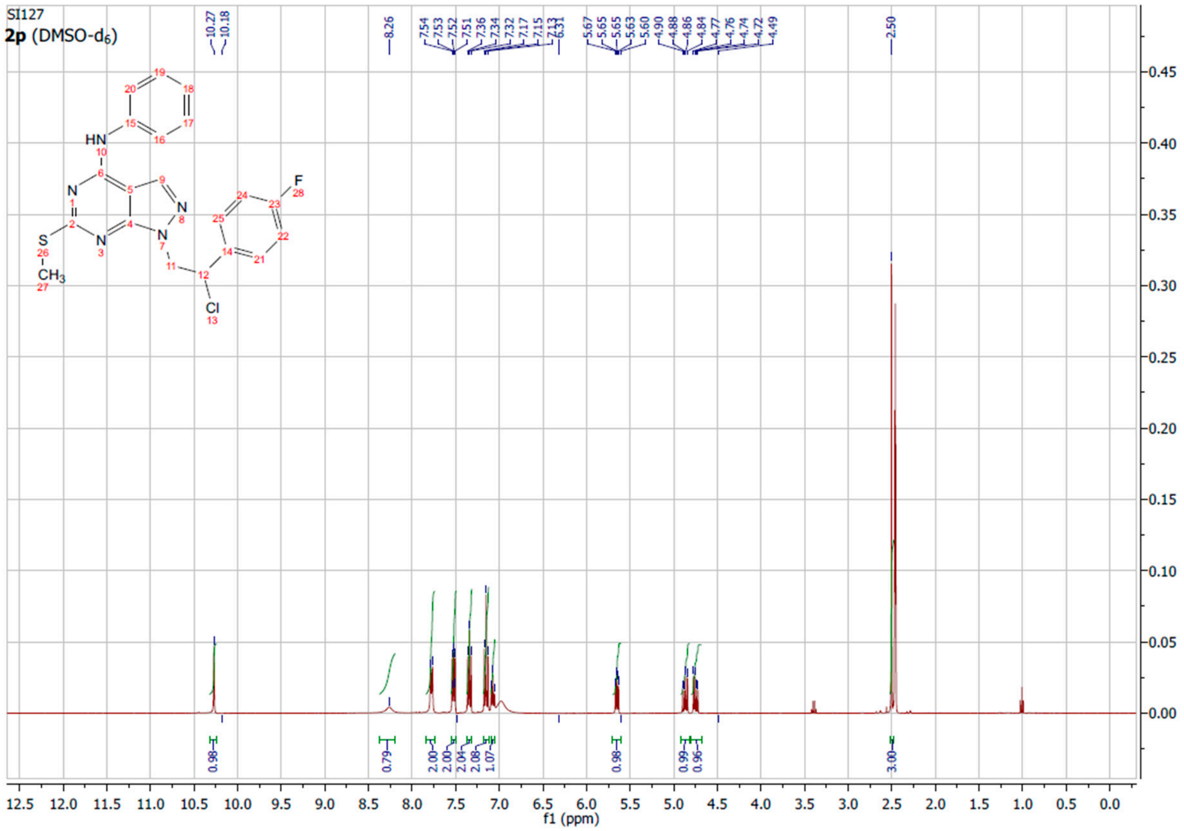
SI127
2p (DMSO-d6)
10.27
10.18
8.26
7.54
7.53
7.52
7.51
7.36
7.34
7.32
7.17
7.15
7.13
6.31
5.67
5.65
5.65
5.63
5.60
4.90
4.88
4.86
4.84
4.77
4.76
4.74
4.72
4.49
2.50
HN
N
N
N
N
S
CH3
F
Cl
0.98
0.79
2.00
2.00
2.04
2.08
1.07
0.98
0.99
0.96
3.00
f1 (ppm)

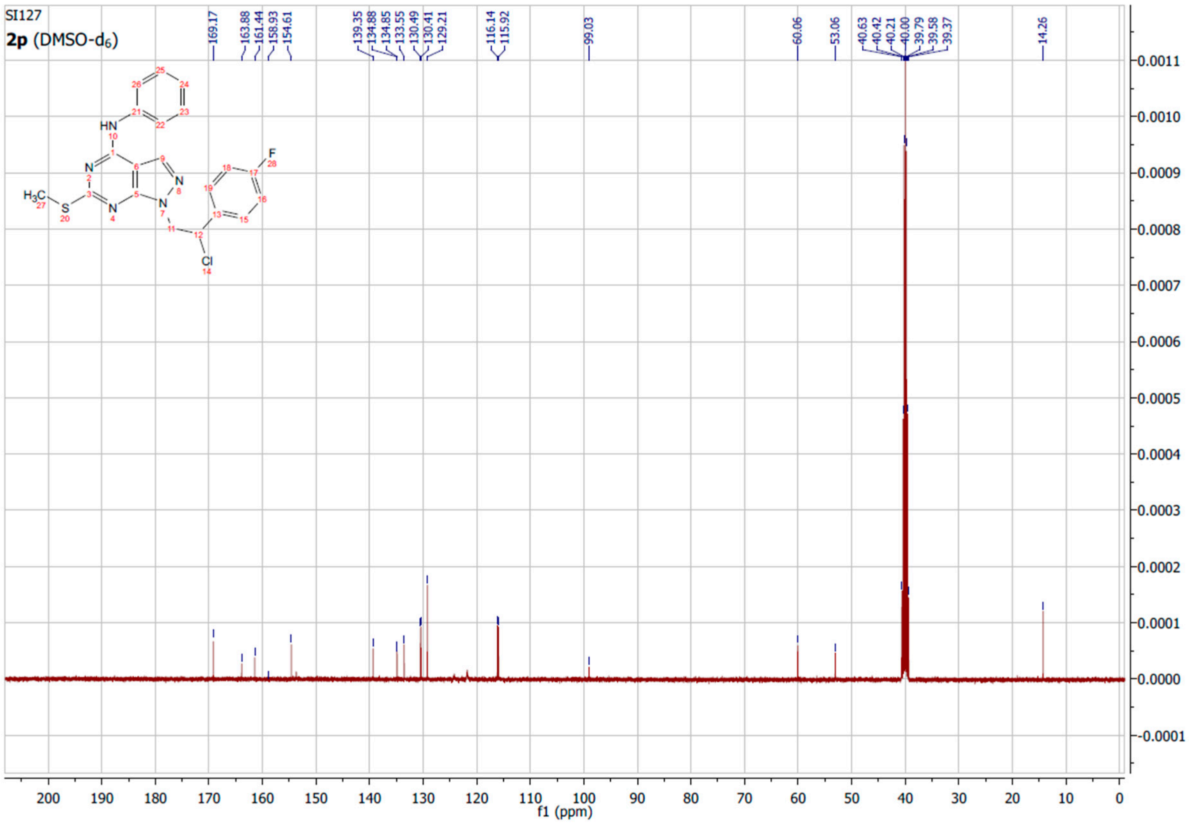
SI127
2p (DMSO-d6)
169.17
163.88
161.44
158.93
154.61
139.35
134.88
134.85
133.55
130.49
130.41
129.21
116.14
115.92
99.03
60.06
53.06
40.63
40.42
40.21
40.00
39.79
39.58
39.37
14.26
HN
N
N
N
N
H3C
S
F
Cl
f1 (ppm)

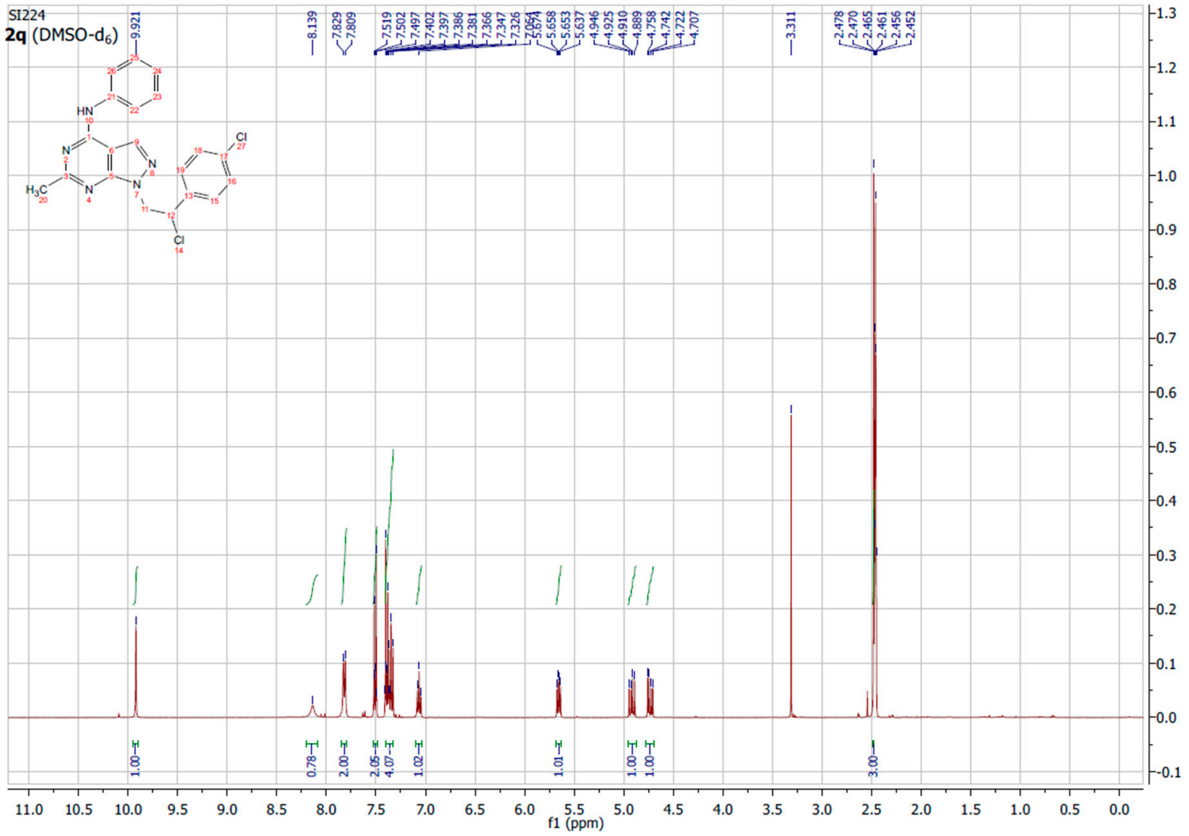

SI224
2q (DMSO-d6)
9.921
8.139
7.829
7.809
7.519
7.502
7.497
7.402
7.397
7.386
7.381
7.366
7.347
7.326
7.054
5.674
5.658
5.653
5.637
4.946
4.925
4.910
4.889
4.758
4.742
4.722
4.707
3.311
2.478
2.470
2.465
2.461
2.456
2.452
HN
N
N
N
N
H3C
Cl
Cl
1.00
0.78
2.00
2.05
4.07
1.02
1.01
1.00
1.00
3.00
f1 (ppm)

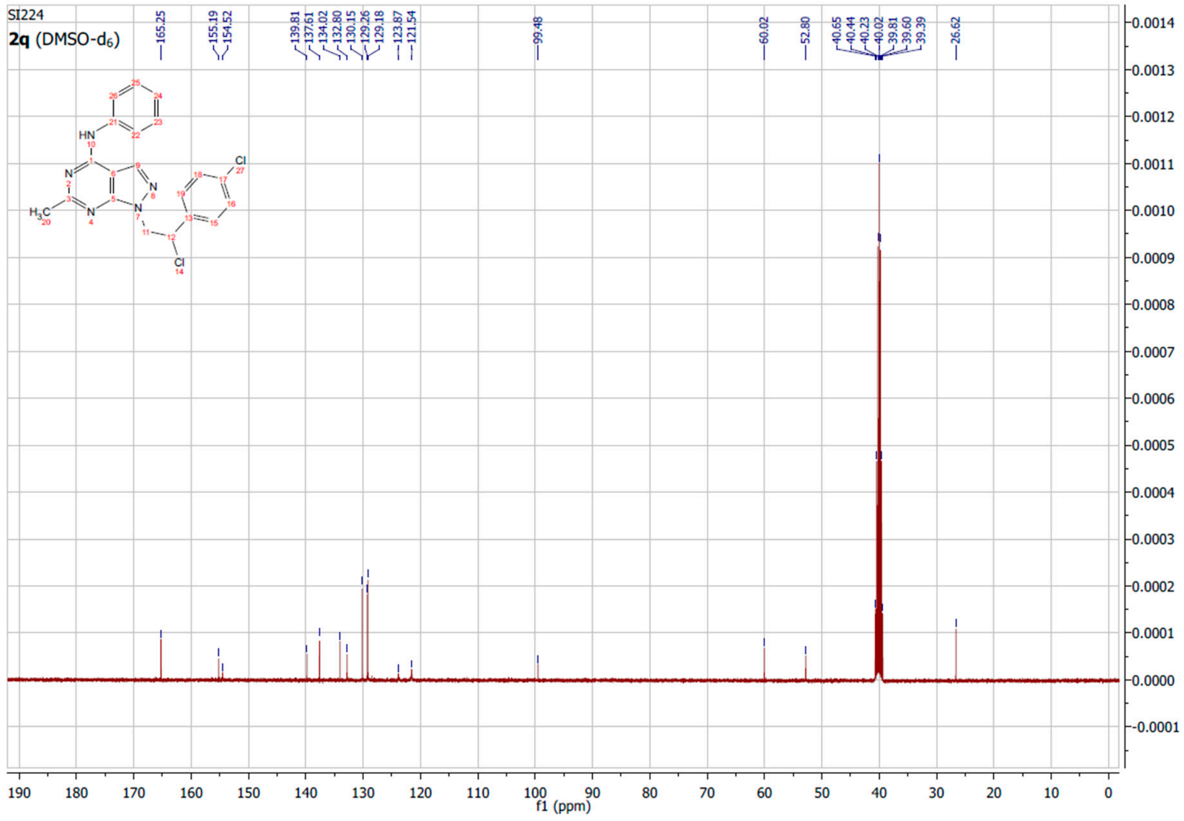

SI224
2q (DMSO-d6)
165.25
155.19
154.52
139.81
137.61
134.02
132.80
130.15
129.26
129.18
123.87
121.54
99.48
60.02
52.80
40.65
40.44
40.23
40.02
39.81
39.60
39.39
26.62
HN
N
N
N
N
H3C
Cl
Cl
f1 (ppm)

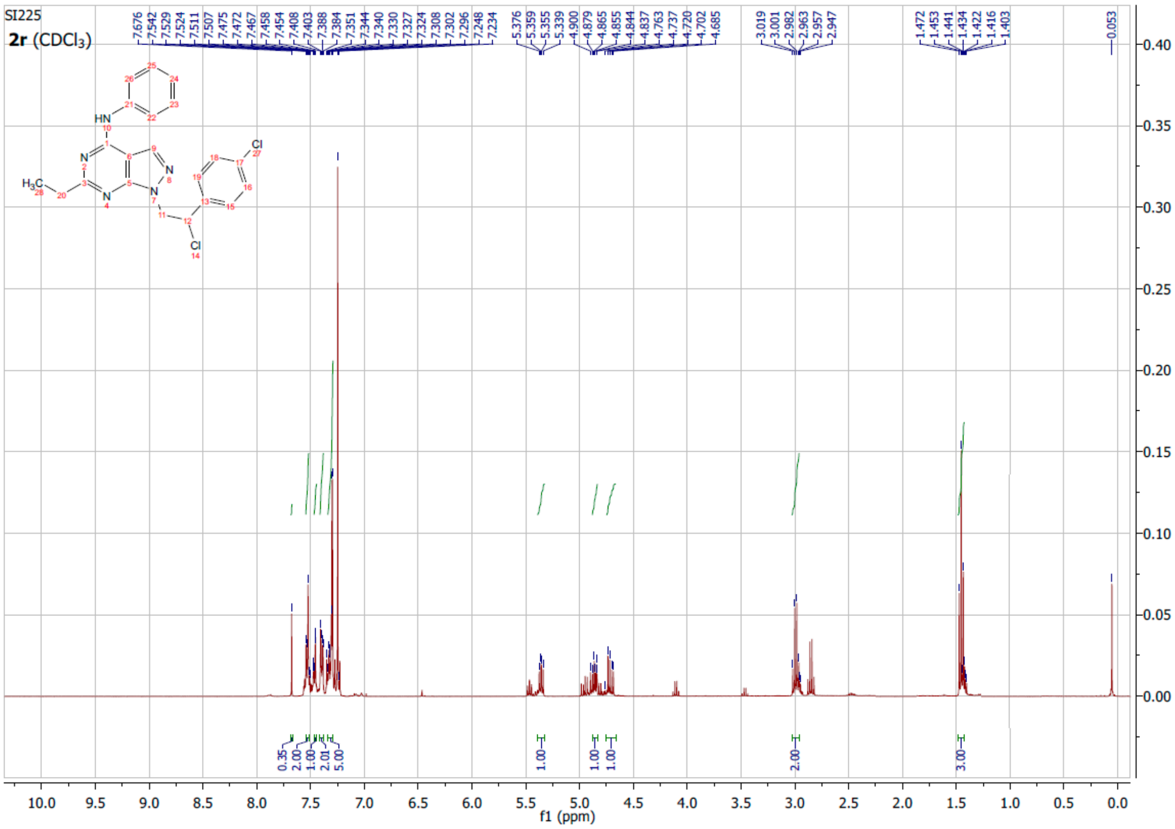
SI225
2r (CDCl3)
f1 (ppm)

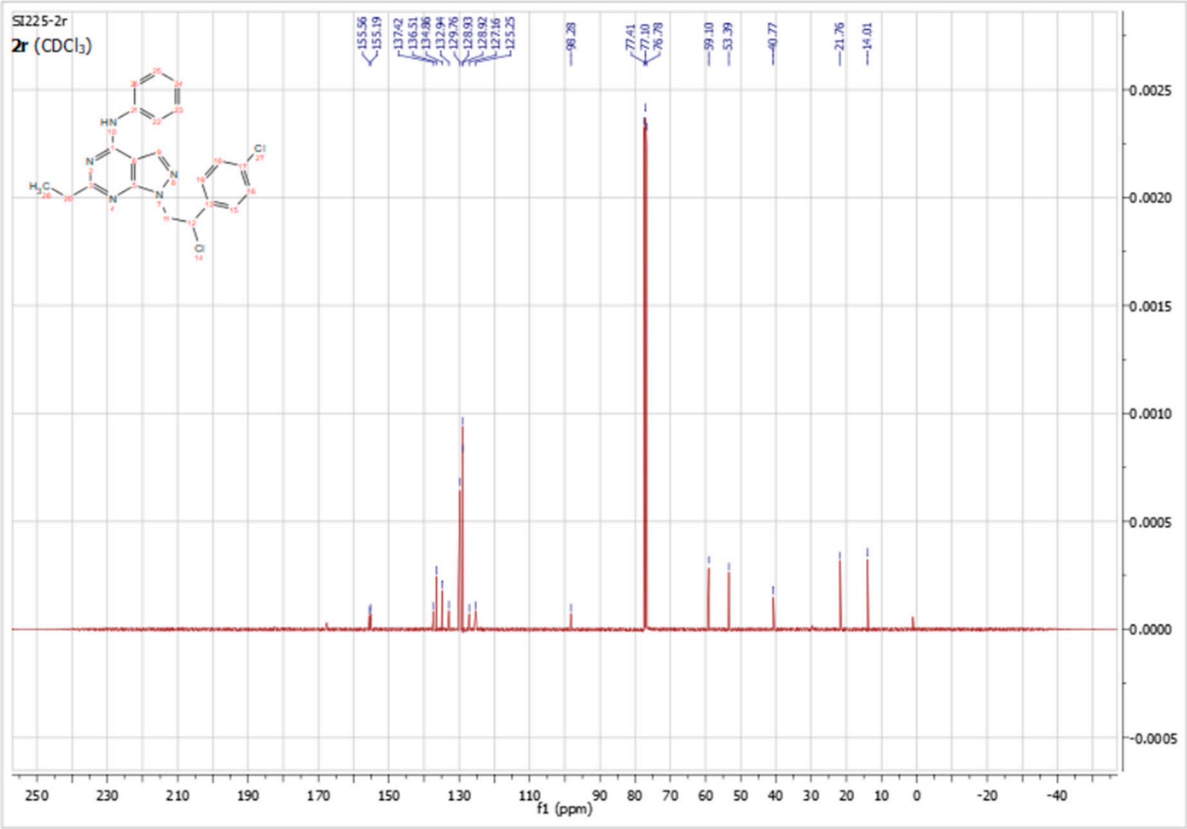
SI225-2r
2r (CDCl3)
f1 (ppm)

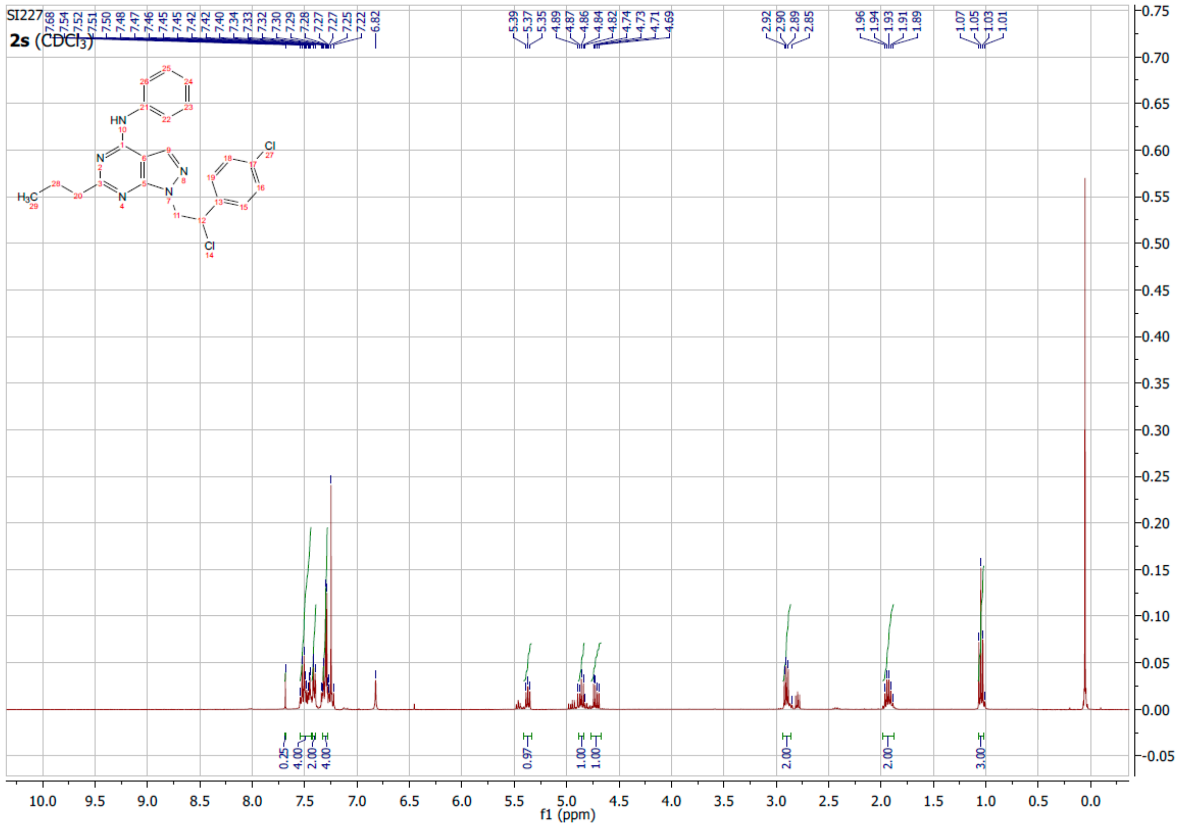
SI227
2s (CDCl3)
f1 (ppm)

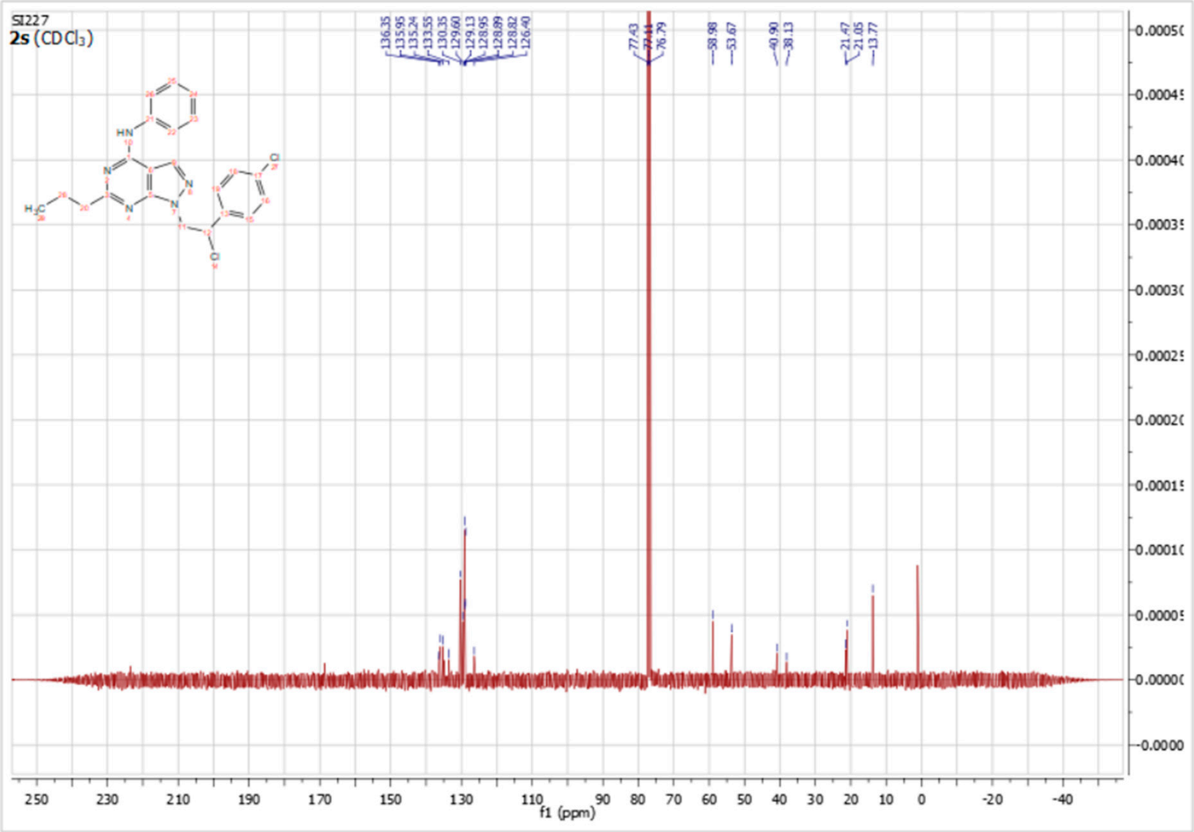
SI227
2s (CDCl3)
f1 (ppm)

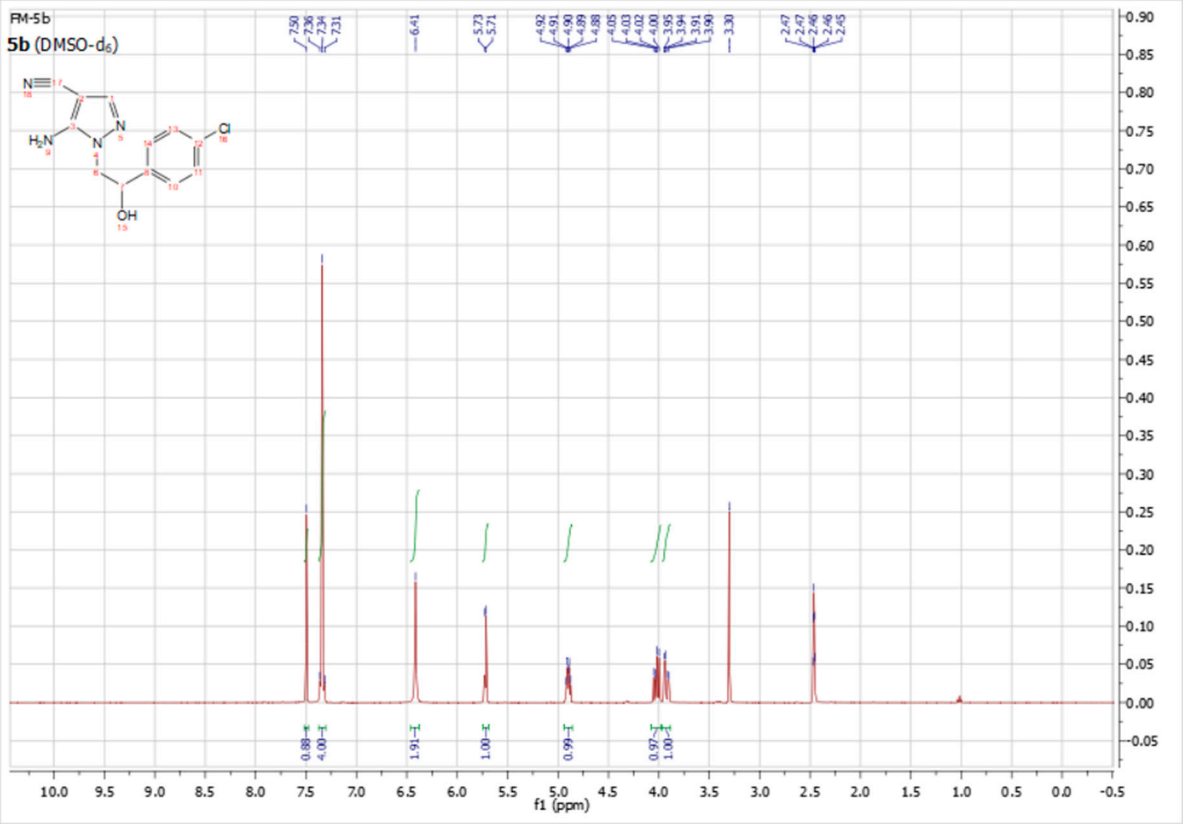
FM-5b
5b (DMSO-d6)
7.50
7.36
7.34
7.31
6.41
5.73
5.71
4.92
4.91
4.90
4.89
4.88
4.05
4.03
4.02
4.00
3.95
3.94
3.91
3.90
3.30
2.47
2.47
2.46
2.46
2.45
0.88
4.00
1.91
1.00
0.99
0.97
1.00
f1 (ppm)

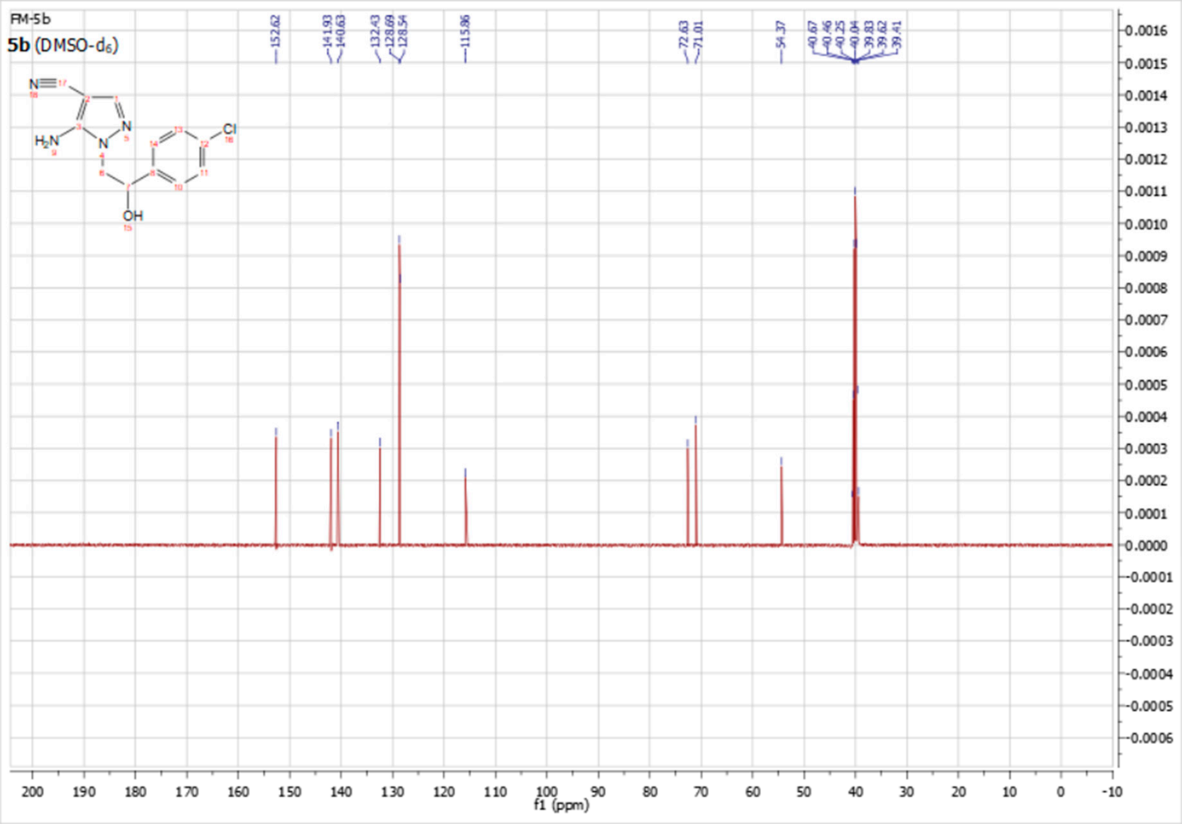
FM-5b
5b (DMSO-d6)
152.62
141.93
140.63
132.43
128.69
128.54
115.86
72.63
71.01
54.37
40.67
40.46
40.25
40.04
39.83
39.62
39.41
f1 (ppm)

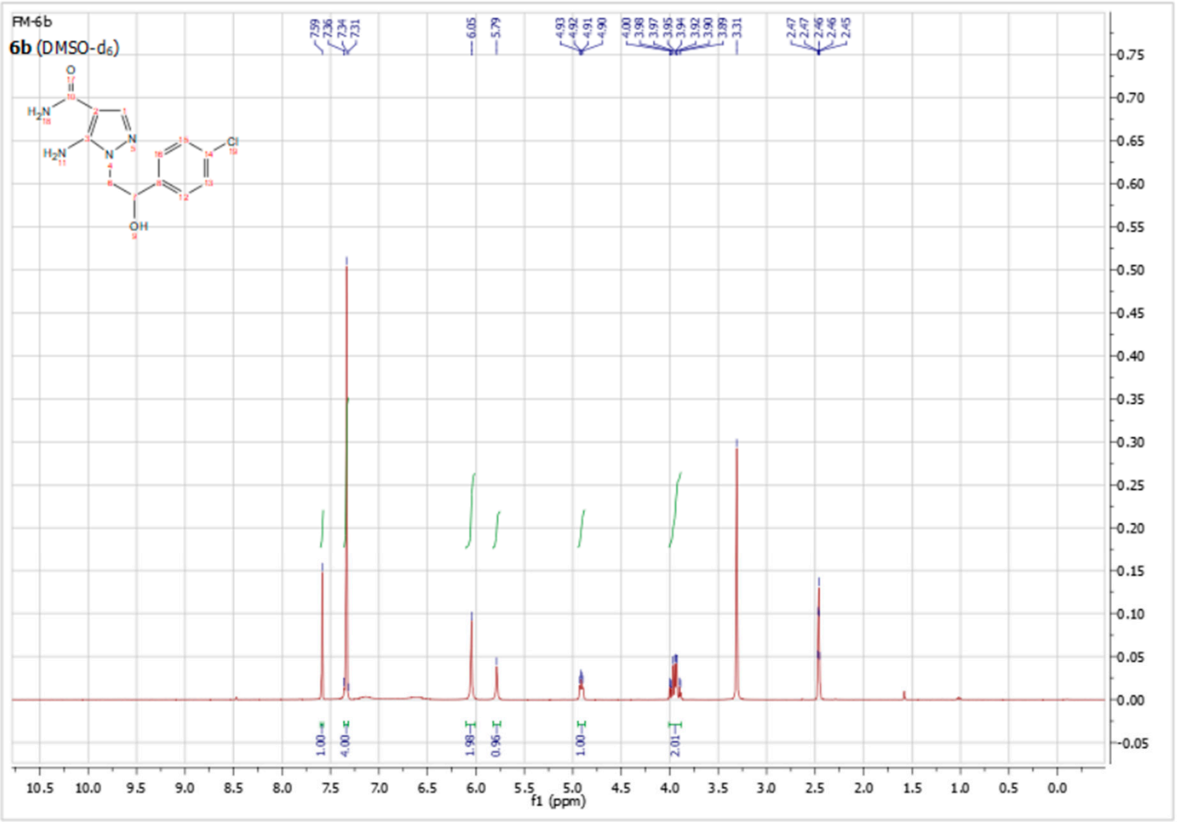
FM-6b
6b (DMSO-d6)
7.59
7.36
7.34
7.31
6.05
5.79
4.93
4.92
4.91
4.90
4.00
3.98
3.97
3.95
3.94
3.92
3.90
3.89
3.31
2.47
2.47
2.46
2.46
2.45
1.00
4.00
1.98
0.96
1.00
2.01
f1 (ppm)

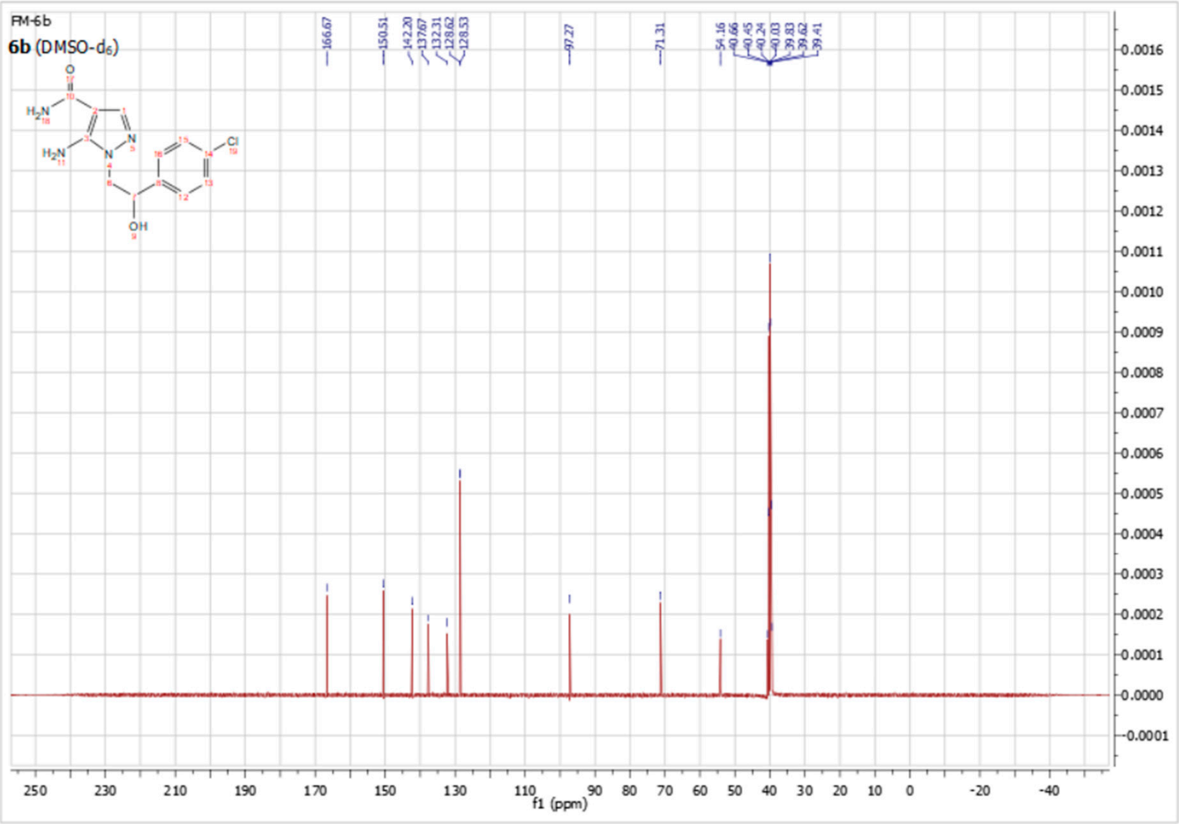
FM-6b
6b (DMSO-d6)
166.67
150.51
142.20
137.67
132.31
128.62
128.53
97.27
71.31
54.16
40.66
40.45
40.24
40.03
39.83
39.62
39.41
f1 (ppm)

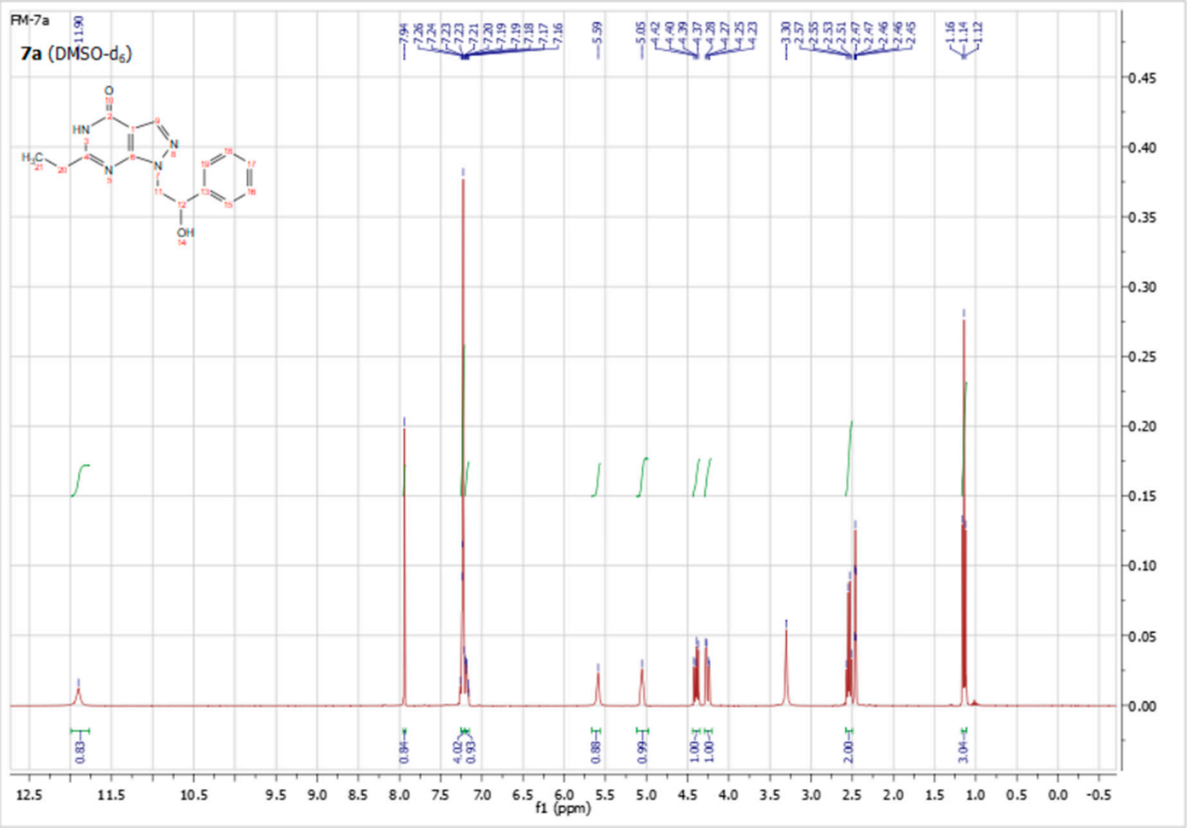
FM-7a
7a (DMSO-d6)
11.90
7.94
7.26
7.24
7.23
7.23
7.21
7.20
7.19
7.19
7.18
7.17
7.16
5.59
5.05
4.42
4.40
4.39
4.37
4.28
4.27
4.25
4.23
3.30
2.57
2.55
2.53
2.51
2.47
2.47
2.46
2.45
1.16
1.14
1.12
0.83
0.84
4.02
0.93
0.88
0.99
1.00
1.00
2.00
3.04
f1 (ppm)

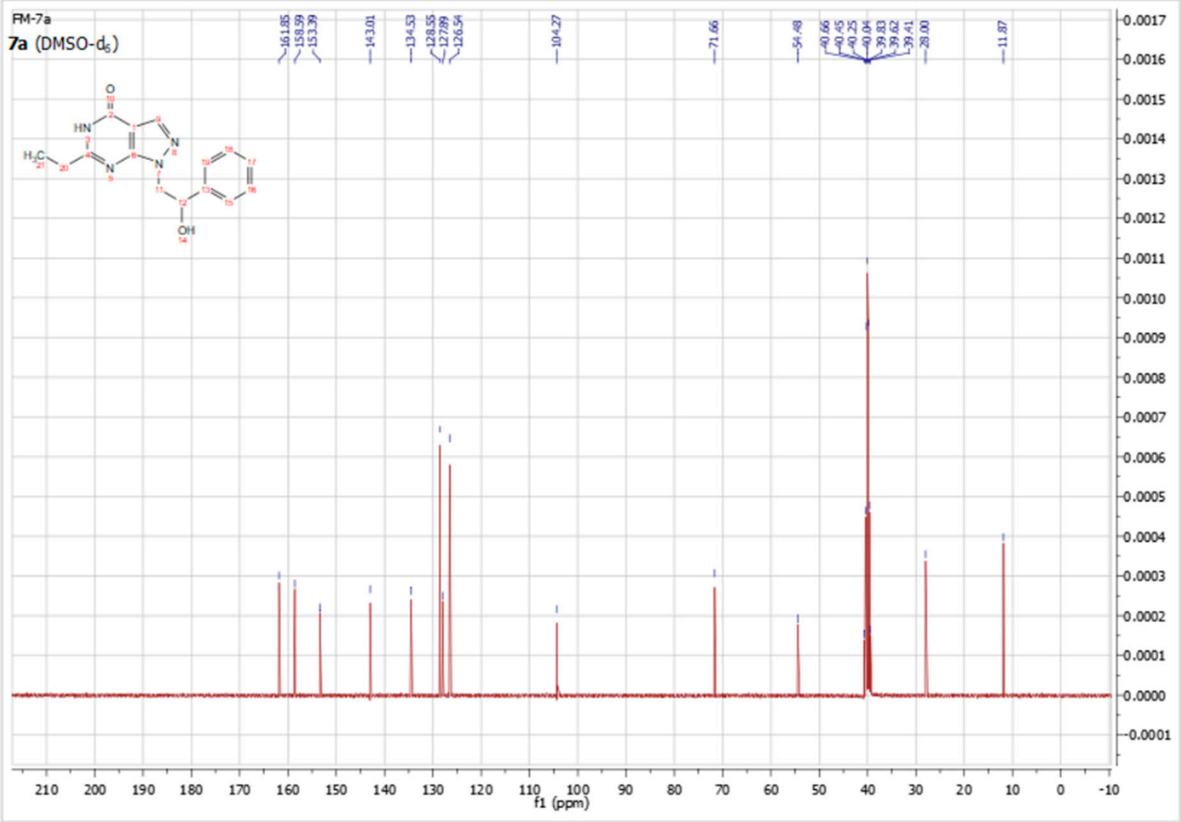
FM-7a
7a (DMSO-d6)
161.85
158.59
153.39
143.01
134.53
128.55
127.89
126.54
104.27
71.66
54.48
40.66
40.45
40.25
40.04
39.83
39.62
39.41
28.00
11.87
f1 (ppm)

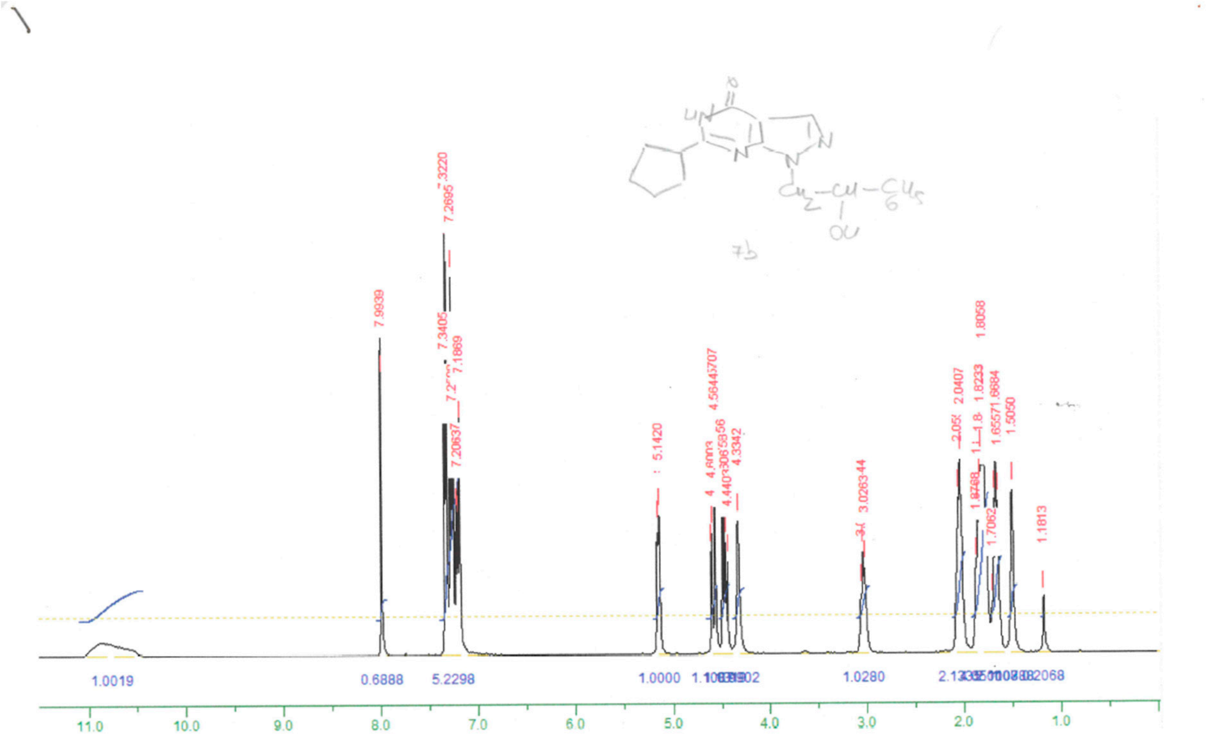
7b
7.9939
5.1420
1.0019
0.6888
5.2298
1.0000
1.0280
1.1813

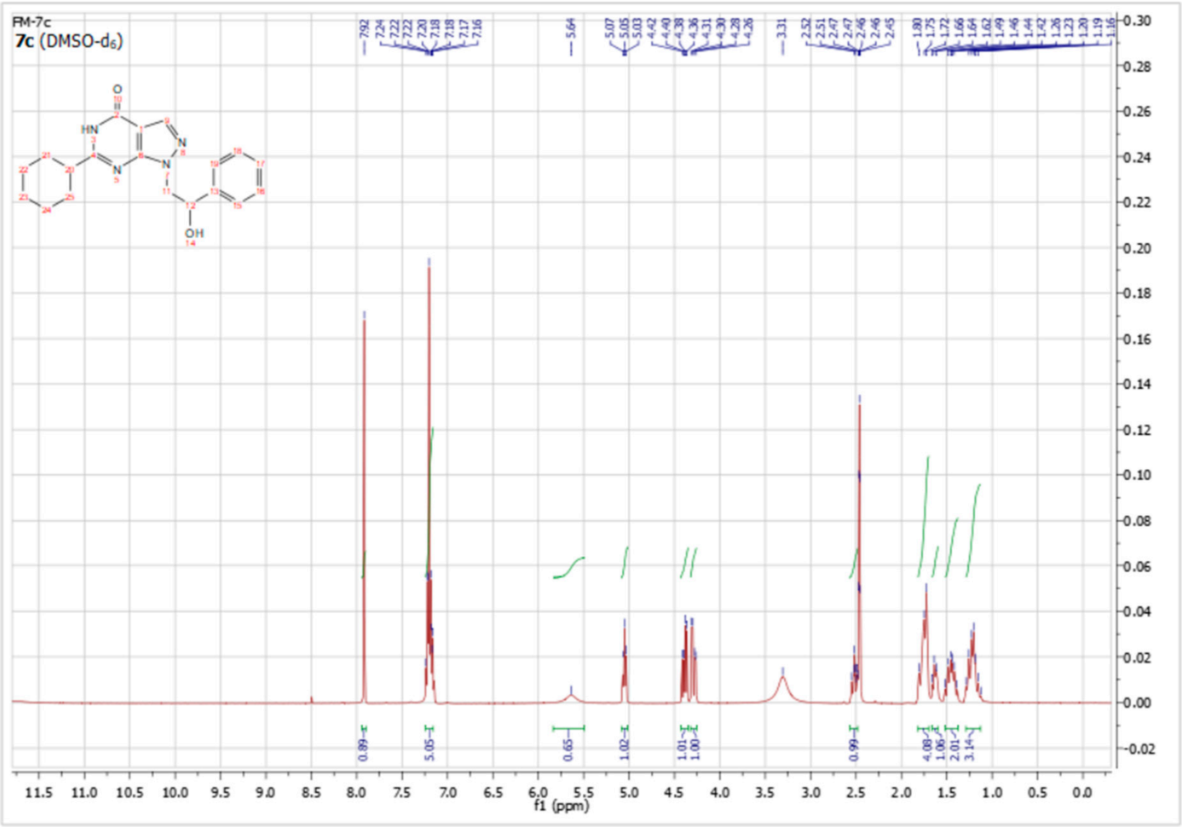
FM-7c
7c (DMSO-d6)
f1 (ppm)

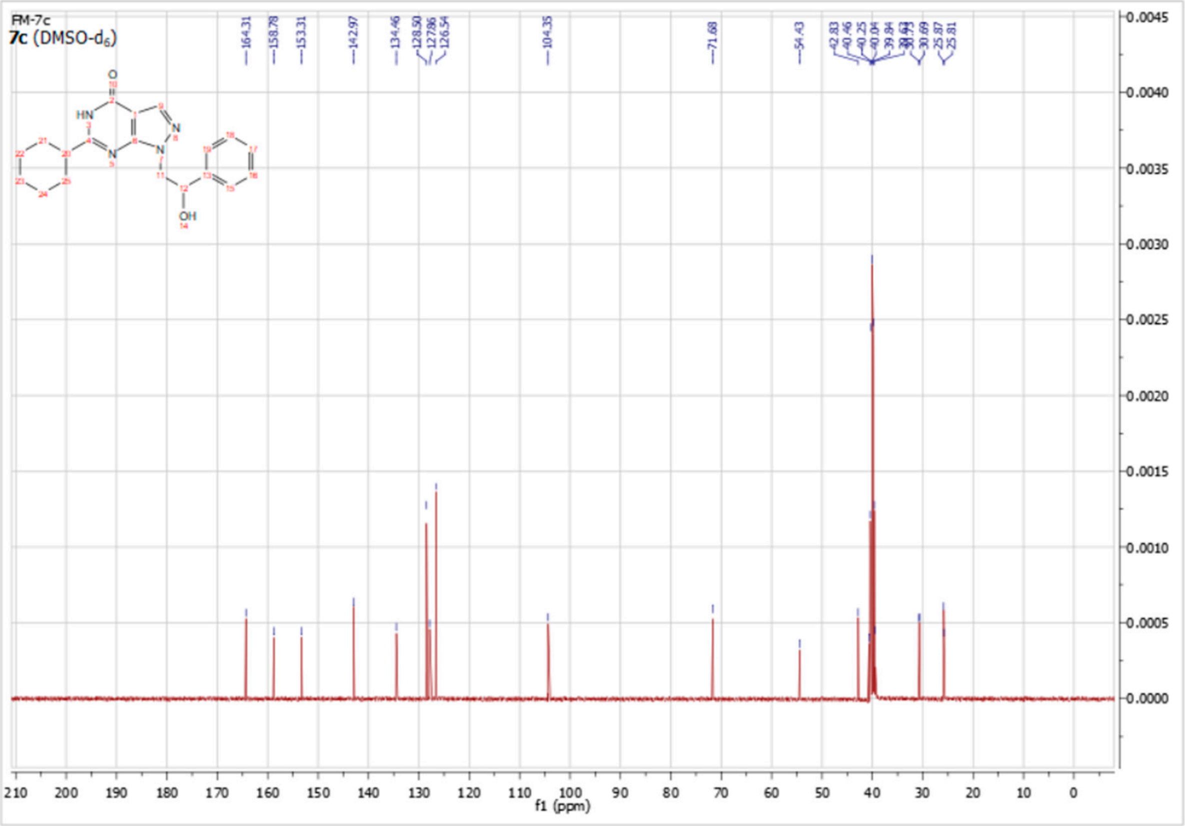
FM-7c
7c (DMSO-d6)
164.31
158.78
153.31
142.97
134.46
128.50
127.86
126.54
104.35
71.68
54.43
42.83
40.46
40.25
40.04
39.84
25.87
25.81
f1 (ppm)

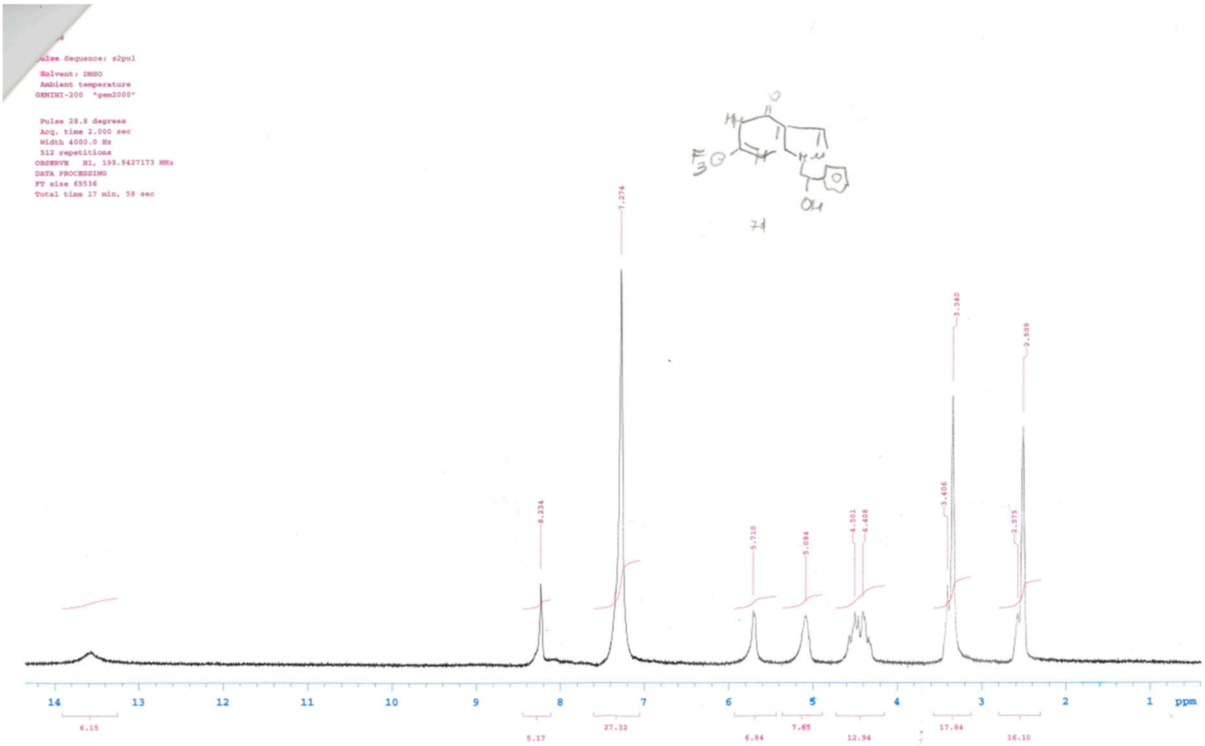
Pulse Sequence: s2pul
Solvent: DMSO
Ambient temperature
GEMINI-200 "gem2000"
Pulse 28.8 degrees
Acq. time 2.000 sec
Width 4000.0 Hz
512 repetitions
OBSERVE H1, 199.9427173 MHz
DATA PROCESSING
FT size 65536
Total time 17 min, 58 sec
7.274
3.340
2.509
8.234
5.710
5.084
4.501
4.408
3.406
2.578
ppm
6.15
5.17
27.32
6.84
7.65
12.94
17.84
16.10

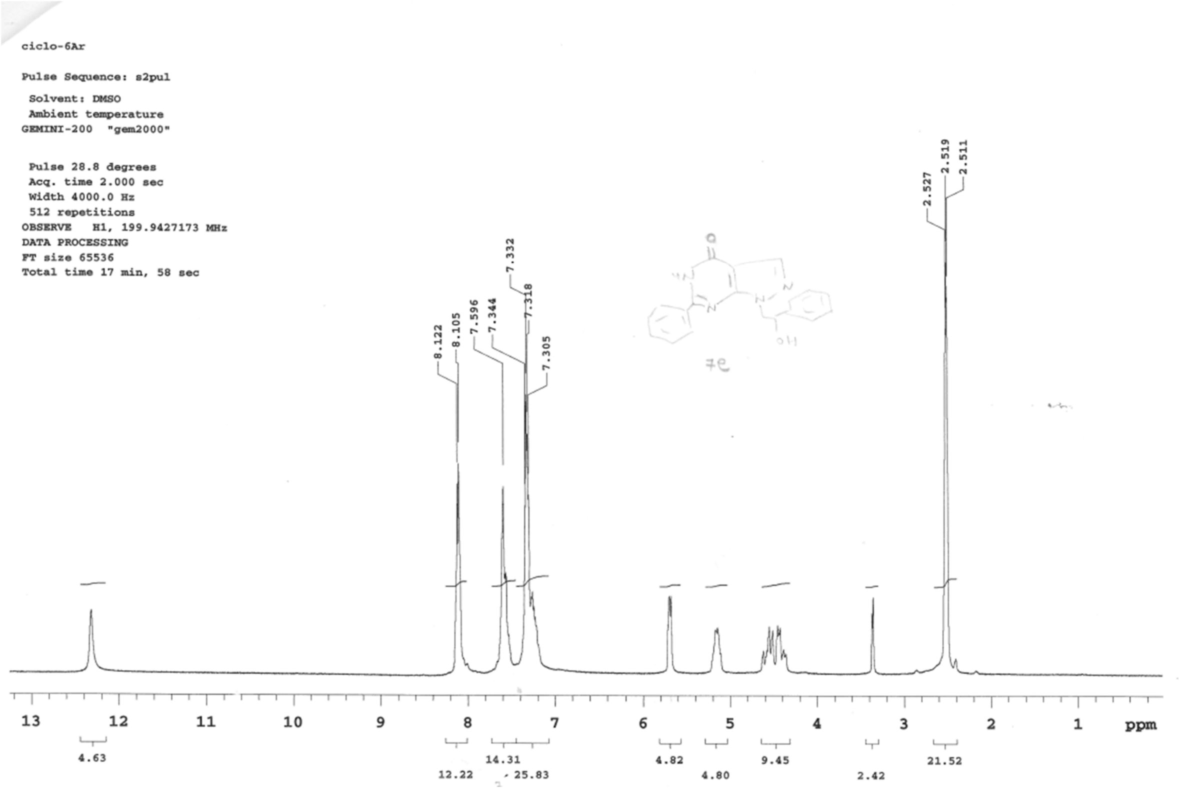
ciclo-6Ar
Pulse Sequence: s2pul
Solvent: DMSO
Ambient temperature
GEMINI-200 "gem2000"
Pulse 28.8 degrees
Acq. time 2.000 sec
Width 4000.0 Hz
512 repetitions
OBSERVE H1, 199.9427173 MHz
DATA PROCESSING
FT size 65536
Total time 17 min, 58 sec
8.122
8.105
7.596
7.344
7.332
7.318
7.305
2.527
2.519
2.511
7e
ppm
4.63
12.22
14.31
25.83
4.82
4.80
9.45
2.42
21.52

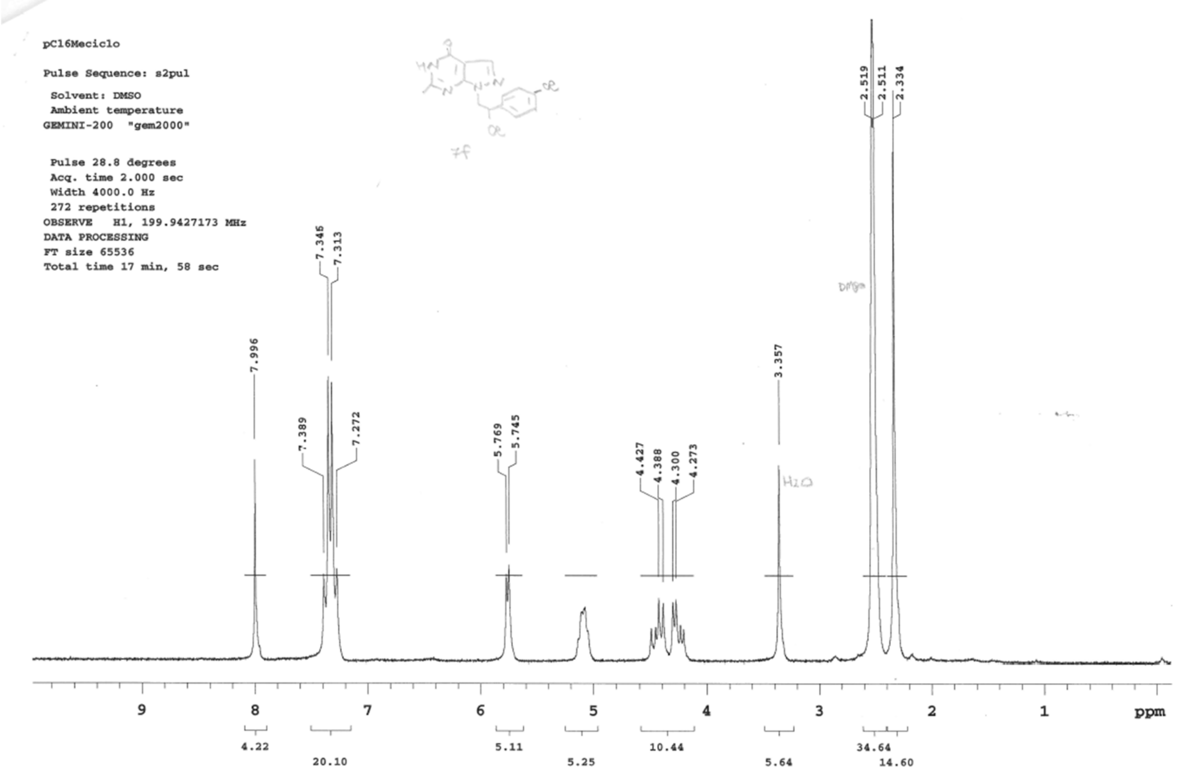
pCl6Meciclo
Pulse Sequence: s2pul
Solvent: DMSO
Ambient temperature
GEMINI-200 "gem2000"
Pulse 28.8 degrees
Acq. time 2.000 sec
Width 4000.0 Hz
272 repetitions
OBSERVE H1, 199.9427173 MHz
DATA PROCESSING
FT size 65536
Total time 17 min, 58 sec
7f
7.996
7.389
7.346
7.313
7.272
5.769
5.745
4.427
4.388
4.300
4.273
3.357
H2O
2.519
2.511
2.334
ppm
4.22
20.10
5.11
5.25
10.44
5.64
34.64
14.60

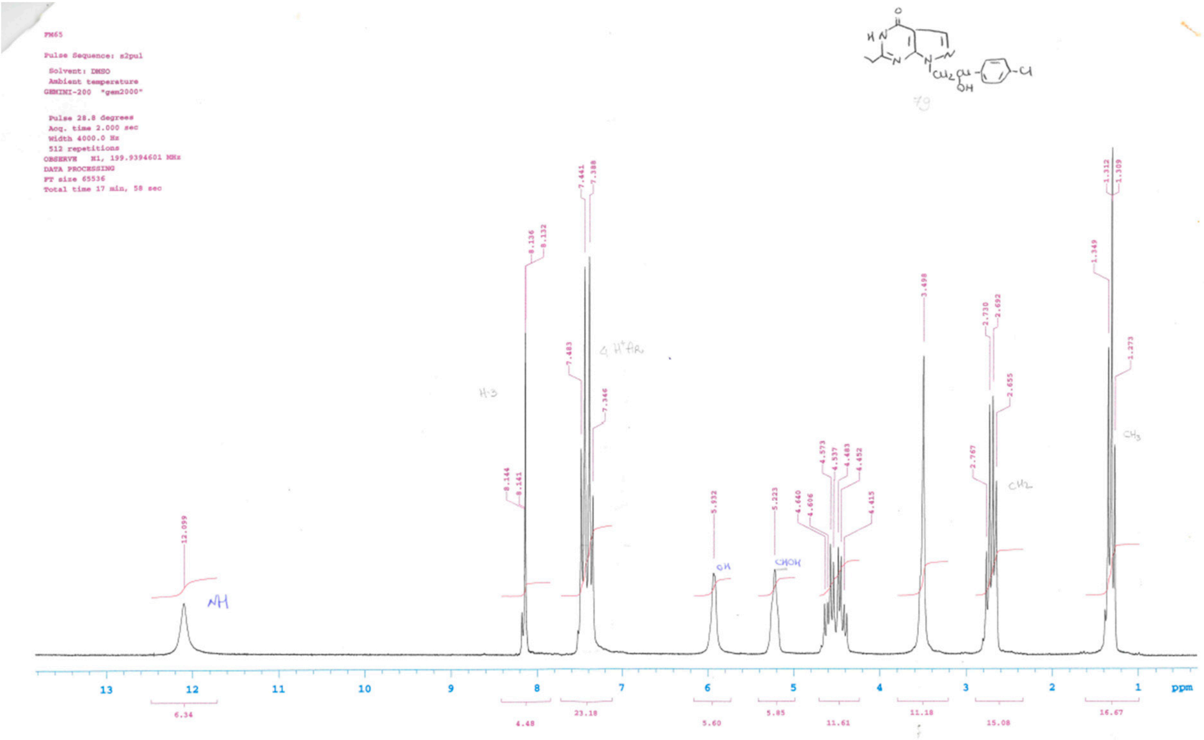
FM65
Pulse Sequence: s2pul
Solvent: DMSO
Ambient temperature
GEMINI-200 "gem2000"
Pulse 28.8 degrees
Acq. time 2.000 sec
Width 4000.0 Hz
512 repetitions
OBSERVE H1, 199.9394601 MHz
DATA PROCESSING
FT size 65536
Total time 17 min, 58 sec
12.099
8.144
8.141
8.136
8.132
7.483
7.441
7.388
7.346
5.932
5.223
4.640
4.606
4.573
4.537
4.483
4.452
4.415
3.498
2.767
2.730
2.692
2.655
1.349
1.312
1.309
1.273
NH
OH
CHOH
CH2
CH3
13 12 11 10 9 8 7 6 5 4 3 2 1 ppm
6.34
4.48
23.18
5.60
5.85
11.61
11.18
15.08
16.67

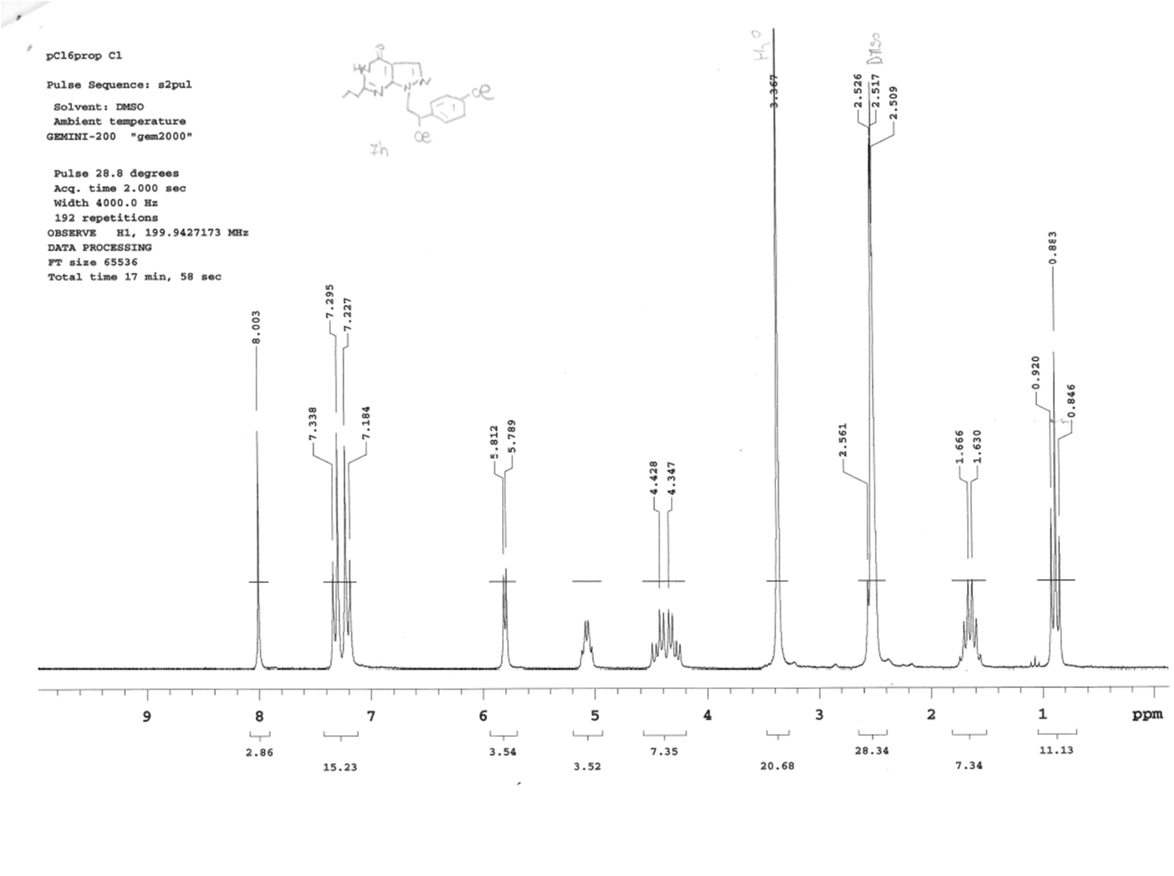
pC16prop Cl
Pulse Sequence: s2pul
Solvent: DMSO
Ambient temperature
GEMINI-200 "gem2000"
Pulse 28.8 degrees
Acq. time 2.000 sec
Width 4000.0 Hz
192 repetitions
OBSERVE H1, 199.9427173 MHz
DATA PROCESSING
FT size 65536
Total time 17 min, 58 sec
8.003
7.338
7.295
7.227
7.184
5.812
5.789
4.428
4.347
3.367
2.561
2.526
2.517
2.509
1.666
1.630
0.920
0.883
0.846
DMSO
9 8 7 6 5 4 3 2 1 ppm
2.86
15.23
3.54
3.52
7.35
20.68
28.34
7.34
11.13

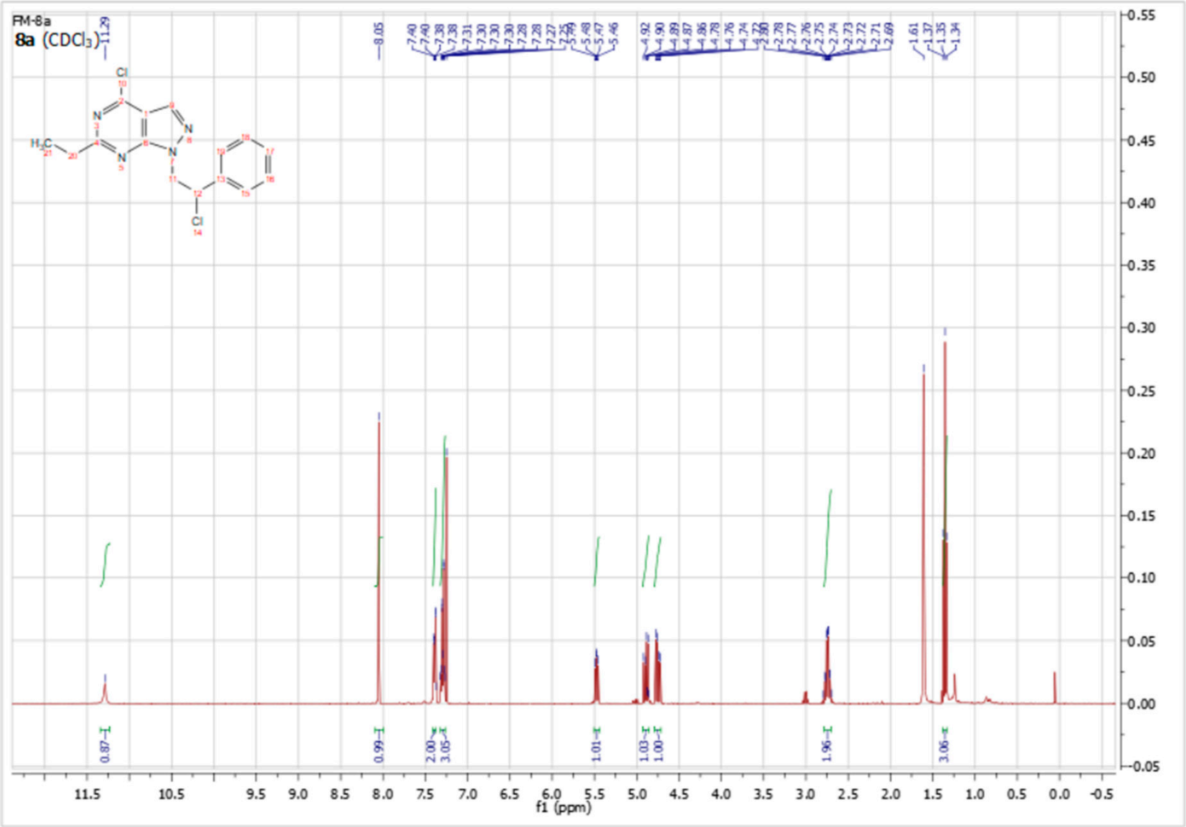
FM-8a
8a (CDCl$_3$)
f1 (ppm)

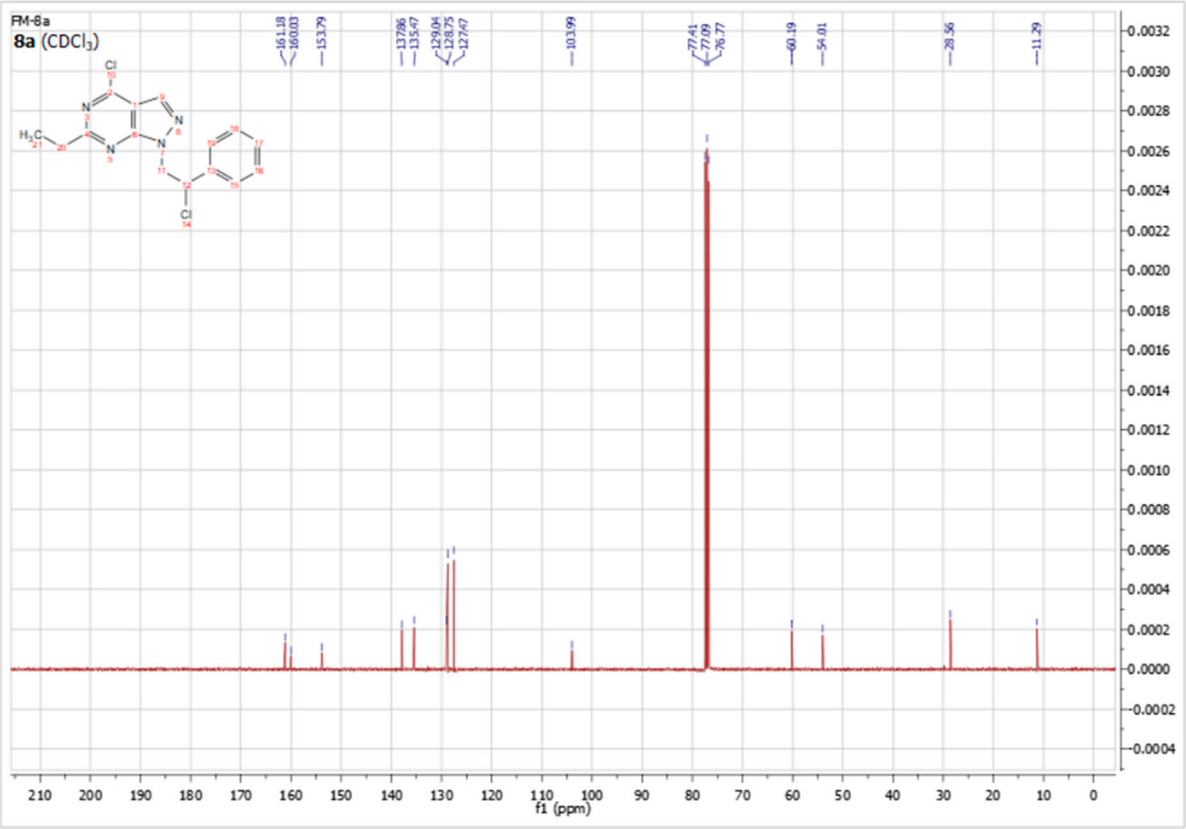
FM-8a
8a (CDCl$_3$)
161.18
160.03
153.79
137.86
135.47
129.04
128.75
127.47
103.99
77.41
77.09
76.77
60.19
54.01
28.56
11.29
f1 (ppm)

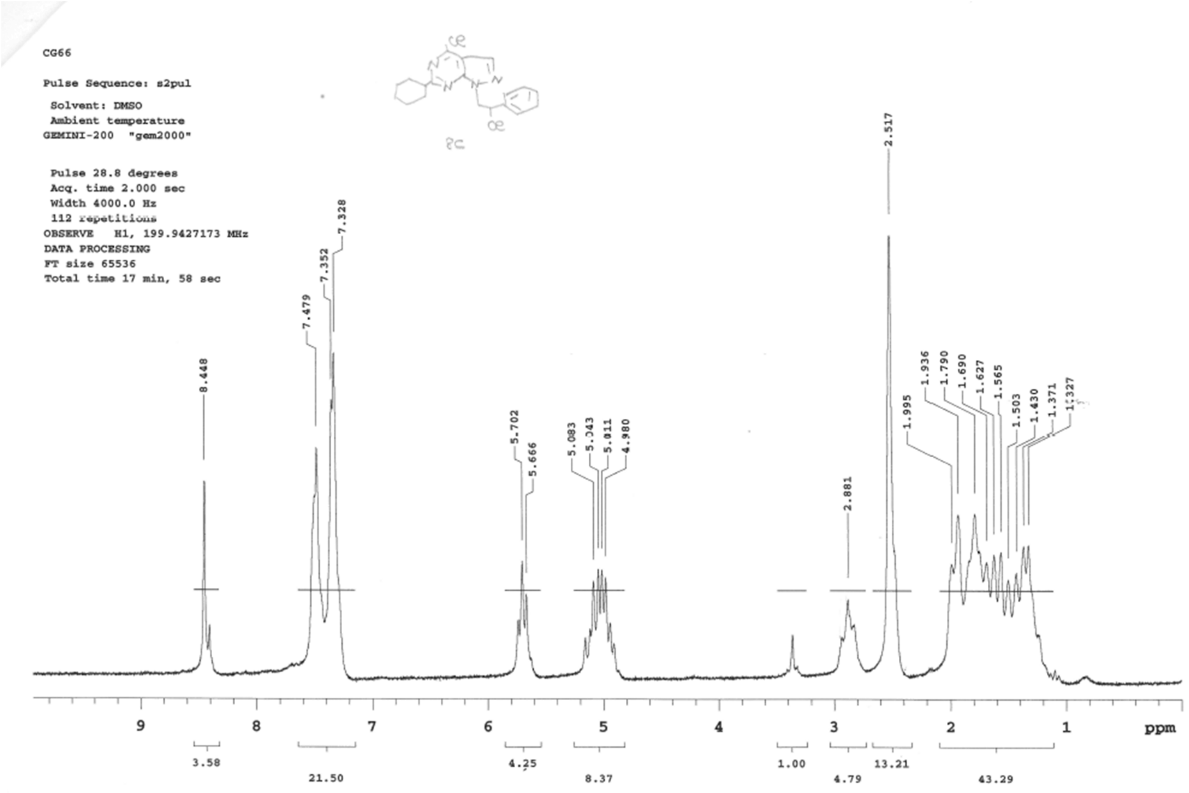
CG66
Pulse Sequence: s2pul
Solvent: DMSO
Ambient temperature
GEMINI-200 "gem2000"
Pulse 28.8 degrees
Acq. time 2.000 sec
Width 4000.0 Hz
112 repetitions
OBSERVE H1, 199.9427173 MHz
DATA PROCESSING
FT size 65536
Total time 17 min, 58 sec
8.448
7.479
7.352
7.328
5.702
5.666
5.083
5.043
5.011
4.980
2.881
2.517
1.995
1.936
1.790
1.690
1.627
1.565
1.503
1.430
1.371
1.327
ppm
3.58
21.50
4.25
8.37
1.00
4.79
13.21
43.29

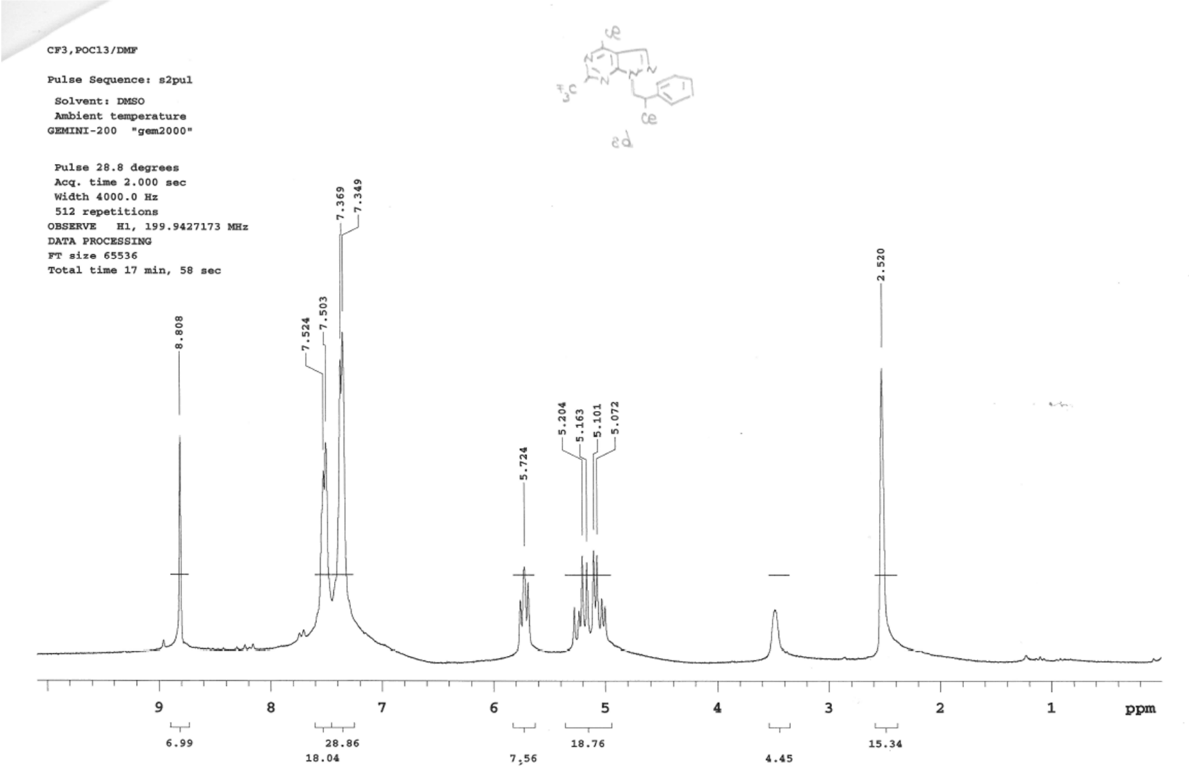
CF3,POCl3/DMF
Pulse Sequence: s2pul
Solvent: DMSO
Ambient temperature
GEMINI-200 "gem2000"
Pulse 28.8 degrees
Acq. time 2.000 sec
Width 4000.0 Hz
512 repetitions
OBSERVE H1, 199.9427173 MHz
DATA PROCESSING
FT size 65536
Total time 17 min, 58 sec
8.808
7.524
7.503
7.369
7.349
5.724
5.204
5.163
5.101
5.072
2.520
ppm
6.99
28.86
18.04
7,56
18.76
4.45
15.34

6Ar Cl

Pulse Sequence: s2pul

Solvent: CDCl3
Ambient temperature
GEMINI-200 "gem2000"

Pulse 28.8 degrees
Acq. time 2.000 sec
Width 4000.0 Hz
48 repetitions
OBSERVE H1, 199.9417676 MHz
DATA PROCESSING
FT size 65536
Total time 17 min, 58 sec

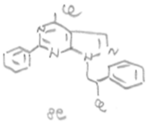

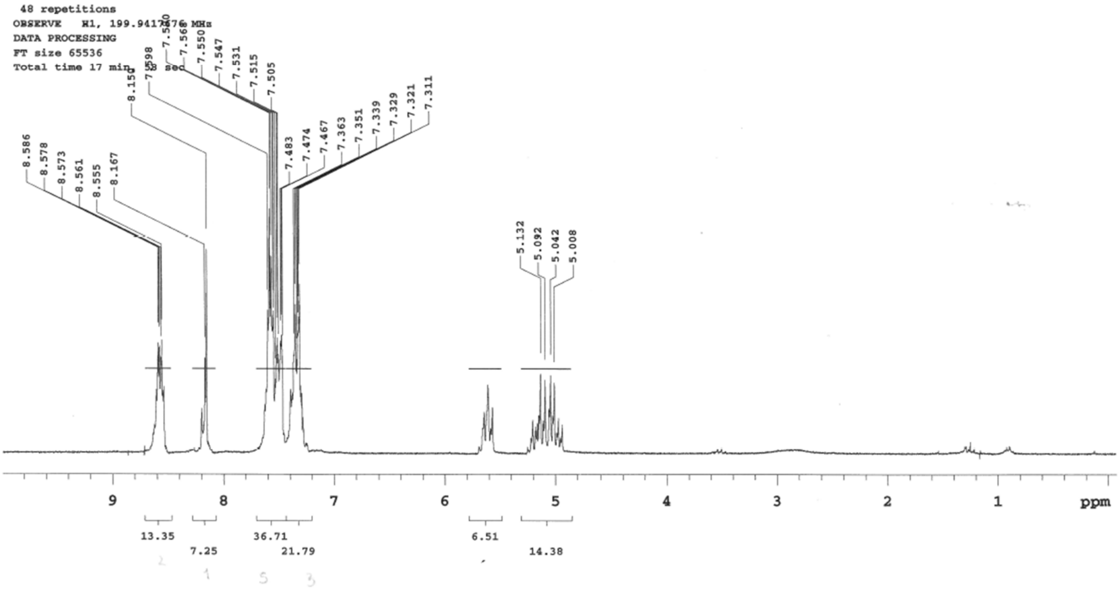

CG63

Pulse Sequence: s2pul

Solvent: CDCl3
Ambient temperature
GEMINI-200 "gem2000"

Pulse 28.8 degrees
Acq. time 2.000 sec
Width 4000.0 Hz
144 repetitions
OBSERVE H1, 199.9417676 MHz
DATA PROCESSING
FT size 65536
Total time 17 min, 58 sec

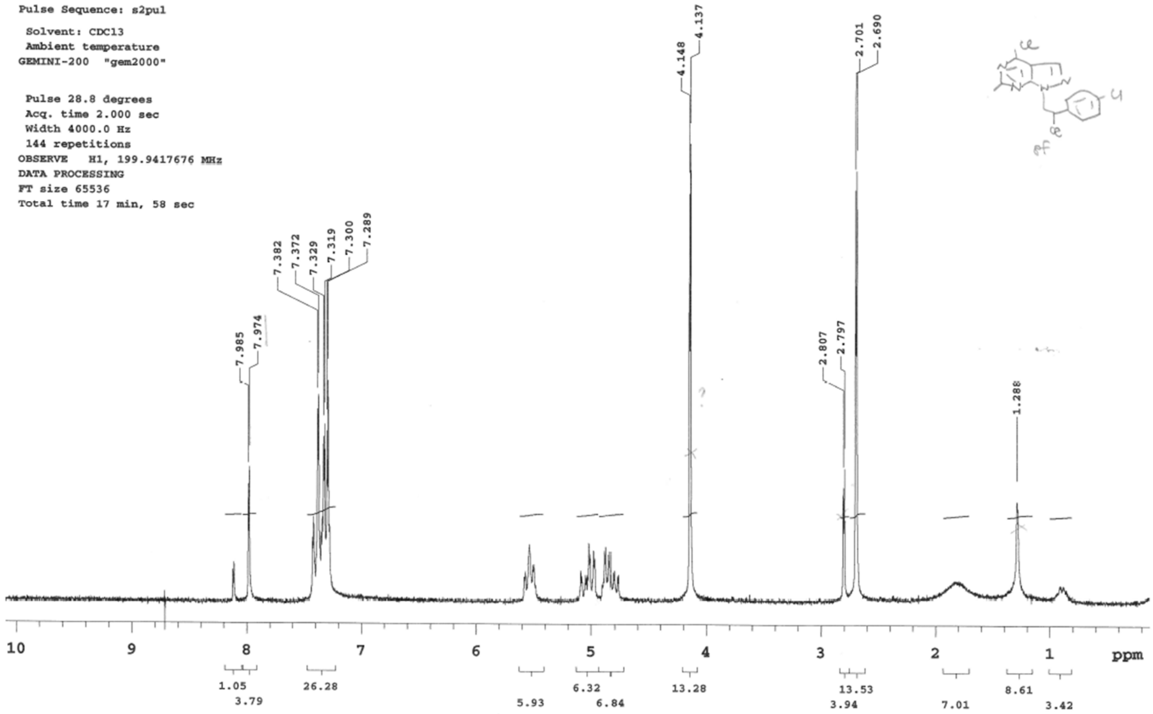

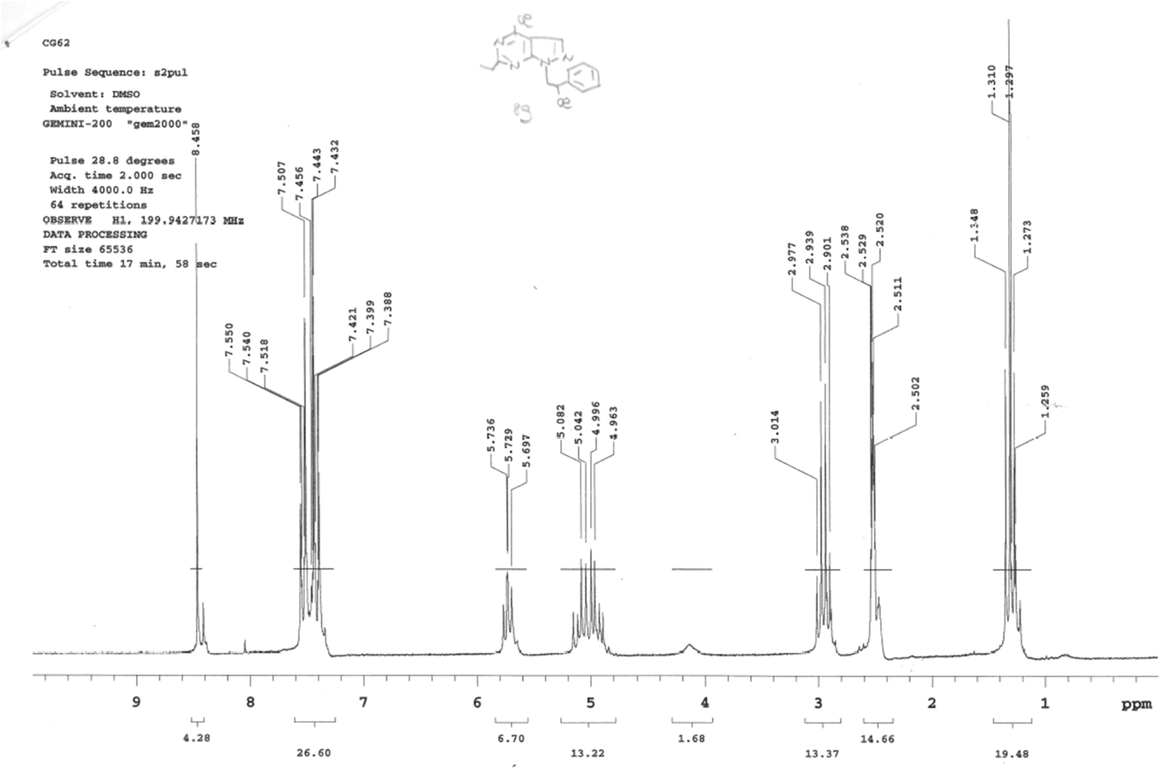
CG62
Pulse Sequence: s2pul
Solvent: DMSO
Ambient temperature
GEMINI-200 "gem2000"
Pulse 28.8 degrees
Acq. time 2.000 sec
Width 4000.0 Hz
64 repetitions
OBSERVE H1, 199.9427173 MHz
DATA PROCESSING
FT size 65536
Total time 17 min, 58 sec
8.458
7.550
7.540
7.518
7.507
7.456
7.443
7.432
7.421
7.399
7.388
5.736
5.729
5.697
5.082
5.042
4.996
4.963
3.014
2.977
2.939
2.901
2.538
2.529
2.520
2.511
2.502
1.348
1.310
1.297
1.273
1.259
9 8 7 6 5 4 3 2 1 ppm
4.28
26.60
6.70
13.22
1.68
13.37
14.66
19.48
